# Synthesis and Antibacterial Activity of New Azole, Diazole and Triazole Derivatives Based on *p*-Aminobenzoic Acid

**DOI:** 10.3390/molecules26092597

**Published:** 2021-04-29

**Authors:** Birutė Sapijanskaitė-Banevič, Vykintas Palskys, Rita Vaickelionienė, Jūratė Šiugždaitė, Povilas Kavaliauskas, Birutė Grybaitė, Vytautas Mickevičius

**Affiliations:** 1 Department of Organic Chemistry, Kaunas University of Technology, Radvilėnų Rd. 19, LT-50254 Kaunas, Lithuania; birute.sapijanskaite@ktu.lt (B.S.-B.); birute.grybaite@ktu.lt (B.G.); vytautas.mickevicius@ktu.lt (V.M.); 2 Thermo Fisher Scientific, V. A. Graičiūno st. 8, LT-02241 Vilnius, Lithuania; vykintas.palskys@themofisher.com; 3 Department of Veterinary Pathobiology, Veterinary Academy, Lithuanian University of Health Sciences, Tilžės Str. 18, LT-47181 Kaunas, Lithuania; siujur@gmail.com; 4 Weill Cornell Medicine of Cornell University, 527 East 68th Street, New York, NY 10065, USA; pok4001@med.cornell.edu; 5 Institute for Genome Sciences, School of Medicine, University of Maryland, 655 W. Baltimore Street, Baltimore, MD 21201, USA; 6 Biological Research Center, Veterinary Academy, Lithuanian University of Health Sciences, Tilžės Str. 18, LT-47181 Kaunas, Lithuania

**Keywords:** hydrazides, 2-pyrrolidinone, azoles, benzimidazole, antimicrobial activity

## Abstract

The *p-*aminobenzoic acid was applied for the synthesis of substituted 1-phenyl-5-oxopyrrolidine derivatives containing benzimidazole, azole, oxadiazole, triazole, dihydrazone, and dithiosemicarbazide moieties in the structure. All the obtained compounds were evaluated for their in vitro antimicrobial activity against *Staphylococcus aureus*, *Bacillus cereus*, *Listeria monocytogenes*, *Salmonella enteritidis, Escherichia coli*, and *Pseudomonas aeruginosa* by using MIC and MBC assays. This study showed a good bactericidal activity of *γ*-amino acid and benzimidazoles derivatives. The antimicrobial activity of the most promising compounds was higher than ampicillin. Furthermore, two benzimidazoles demonstrated good antimicrobial activity against *L. monocytogenes* (MIC 15.62 µg/mL) that was four times more potent than ampicillin (MIC 65 µg/mL). Further studies are needed to better understand the mechanism of the antimicrobial activity as well as to generate antimicrobial compounds based on the 1-phenyl-5-oxopyrrolidine scaffold.

## 1. Introduction

Rapidly growing antimicrobial resistance (AMR) has become a major source of morbidity and mortality worldwide [1]. Increasing AMR among various pathogens has led to fewer treatment options for patients suffering from severe infections caused by drug-resistant (DR) pathogens. Moreover, infections caused by DR microorganisms require more extensive treatment, therefore resulting in a longer course of illness and prolonged hospitalization duration [2,3].

The extensive use of various antimicrobials in agriculture and veterinary sectors played a pivotal role in the development of AMR and the selection of highly virulent bacterial strains [4,5,6,7]. The DR pathogens of veterinary origin can further colonize the environment and can be transferred to humans [8,9]. In addition, the genetic determinants encoding AMR phenotypes can be further disseminated via horizontal gene transfer and accumulate in various bacterial species [10,11]. These processes created a vicious cycle that gave rise to multidrug-resistant (MDR) pathogens harboring multiple resistance mechanisms, resulting in bacterial resistance to two and more antimicrobial drugs [12]. To overcome this problem, it is important to develop novel compounds targeting MDR pathogens.

The ESKAPE group of pathogens (*Enterococcus faecium*, *Staphylococcus aureus*, *Klebsiella pneumoniae*, *Acinetobacter baumannii*, *Pseudomonas aeruginosa*, and *Enterobacter* species complex) is the leading cause of hospital-acquired infections worldwide [13,14,15]. This group of Gram-positive and Gram-negative bacteria causes life-threatening infections amongst critically and chronically ill or immunocompromised individuals [15]. The growing antimicrobial resistance among ESKAPE pathogens creates a significant burden on healthcare systems and has important global economic costs. Therefore, it is important to develop novel compounds targeting clinically relevant multidrug-resistant ESKAPE group of pathogens.

*p-*Aminobenzoic acid (*p*ABA) and its derivatives are well-known for their chemical properties and the broad spectrum of biological activity, and they have attracted considerable pharmacological and industrial interest. *p*ABA is widely distributed in nature and is abundant in various plant and animal tissues. Moreover, *p*ABA is found in various animal and plant-based sources such as grains, eggs, milk, and meat. Furthermore, *p*ABA is frequently found as a structural moiety in drugs and plays an important role as a pharmacophore. In a voluminous database of commercial pharmaceuticals, 1.5% were found to contain the *p*ABA moiety [16].

Various compounds bearing a *p*ABA nucleus exert strong antimicrobial [17,18], antimutagenic [19], antioxidant, cytoprotective [20], and immunomodulatory [21,22] properties. Moreover, various *p*ABA derivatives have antineoplastic, anesthetic, antiarrhythmic, anticonvulsant, antiemetic, and gastrokinetic [16,23] properties. *p*ABA is also involved in the biosynthesis of coenzyme Q [24,25]; it also increases the thermotolerance [26] in plants and plays an important role as a signaling molecule in the recognition of the plant pathogens [27]. Various *p*ABA derivatives were previously explored as promising immunomodulatory agents. *p*ABA was shown to induce the transcriptional activation of interferons in various cell types [22]. In addition to this, compounds bearing the *p*ABA nucleus were found to be a promising cyclophilin inhibitors [28]. Furthermore, *p*ABA derivatives were shown to prevent steel corrosion [29] and increase dye adsorption during textile dyeing [30]. The wide spectrum of biological activity of *p*ABA derivatives could be potentially exploited for the development of novel antimicrobial and immunomodulatory drugs.

The 5-oxopyrrolidine or *γ*-lactam moiety is a constituent of many natural and nonnatural biologically active compounds. The broad range of biological activity displayed by functionalized 2-pyrrolidinones makes them an attractive group with profound therapeutic [31] and antioxidant [32] use. Moreover, compounds bearing *γ*-lactam moiety were previously demonstrated to bind the CCR4 chemokine receptor, making these compounds potential therapeutics in treating T-cell neoplasms [33]. Besides immunomodulatory and antiviral activity, 5-oxopyrrolidine derivatives were shown to have promising antimicrobial [34,35], antioxidative [36], antitumor [37], and anti-inflammatory [38] properties, showing high binding affinity towards carbonic anhydrase isoforms [39,40].

The benzimidazole scaffold attracts considerable attention due to its numerous biological properties. One of the most known structures containing the benzimidazole scaffold is cyanocobalamin, also known as cobalamin (vitamin B_12_), which is involved in cellular metabolism processes.

With a great affinity displayed towards a numerous enzymes and receptors, the benzimidazoles could be potentially explored for the development of new pharmaceuticals. Numerous studies revealed that compounds derived from a benzimidazole nucleus exhibit analgesic and anti-inflammatory activity [41], as well as antiulcerative [42], anticancer [43], antiparasitic [44], antimicrobial [45,46,47], antioxidant [46], anticonvulsant [48], anticancer/antiestrogenic [49,50], antihypertensive [51], antifouling [52], and many others [53,54,55,56,57,58,59,60] activities. Therefore, the strategies of developing compounds bearing the benzimidazole scaffold should be further explored to generate pharmacologically active molecules.

1,2,4-Triazole derivatives have been reported to possess a wide range of bioactivities such as neuroprotective [61], antifungal [62], anticancer [63], antibacterial [64], antihypertensive, and cardioprotective [65], antiviral [66], and anticonvulsant [67]. The diverse pharmaceutical properties of triazoles induced a deep interest in discovering new entities for their broader applications. This fragment is a constituent of a variety of pharmaceuticals (etizolam, estazolam, trazodone, ribavirin, trapidil, rizatriptan, anastrozole) that are available in a clinical setting for the treatment of patients suffering from various diseases, including muscle tension, suppression of seizures, depression, viral diseases, antiplatelet action, migraine pains and breast cancer [68,69]. The introduction of the thione group, either in 3- or 5-position, leads to an enhancement of biological activities related to triazole moiety [70]. The triazolethione system is a cyclic analog of very important components (such as thiosemicarbazides) that have effective biological applications [71].

The profound biological activity of *p*ABA and various azoles makes them attractive building blocks for the development of novel antimicrobial compounds targeting clinically important pathogens. With this notion, in this paper we aim to synthesize a series of new azole, diazole, and triazole derivatives based on *p*-aminobenzoic acid and evaluate their in vitro antimicrobial properties against clinically important bacterial pathogens.

## 2. Results

### 2.1. Synthesis

**Scheme 1:**
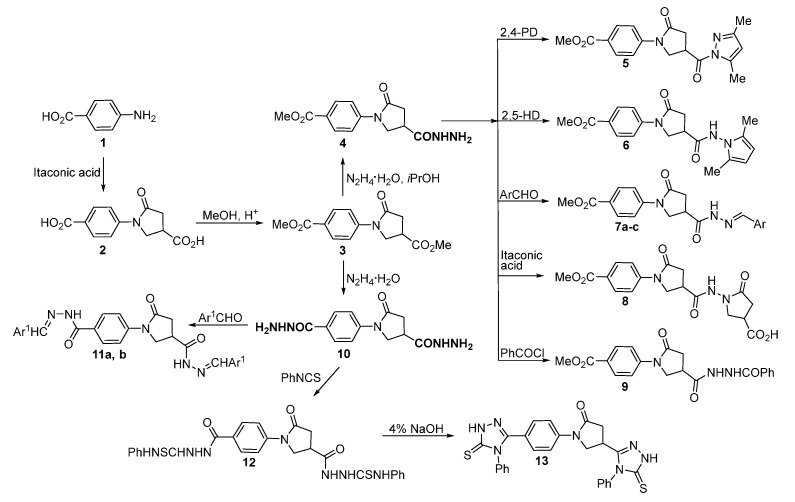
Synthesis of 5-oxopyrrolidine derivatives **2**–**13**. **7a**, Ar = C_6_H_5_; **7b**, Ar = 4-MeO-C_6_H_4_; **7c**, Ar = 4-Me_2_N-C_6_H_4_; **11a**, Ar^1^ = 4-O_2_N-C_6_H_4_; **11b**, Ar^1^ = 4-Cl-C_6_H_4_.

Considering the wide structural and biological diversity of *p*-aminobenzoic acid and its derivatives, herein we present the synthesis and antibacterial evaluation of a series of 1-(4-carboxyphenyl)-5-oxopyrrolidine-3-carboxylic acid derivatives using *p-*aminobenzoic acid as the starting compound (Scheme 1). Compound **2** was prepared from the 4-aminobenzoic and itaconic acids by the method described in [72]. The esterification of compound **2** with methanol afforded methyl ester **3**, which then under the action of hydrazine monohydrate in 2-propanol was converted into hydrazide **4**, containing only one hydrazinocarbonyl moiety.

The hydrazide functional group can undergo various chemical transformations; using this ability, we thus performed a series of chemical reactions, applying different carbonyl compounds. The reaction of hydrazide **4** with pentane-2,4-dione (2,4-PD) in refluxing ethanol produced pyrazole derivative **5**, and the Paal–Knorr pyrrole synthesis using hexane-2,5-dione (2,5-HD) afforded pyrrole **6**. A catalytic amount of glacial acetic acid was used in the reaction. In the ^1^H NMR spectrum of **6**, the intense singlets at 2.0 and 5.65 ppm were assigned to the protons of two methyl (2- and 5-positions) and two C=CH groups of the pyrrole ring, respectively. The resonances at 103.10 and 126.74 ppm in the ^13^C NMR spectrum of compound **6** finally approved the formed pyrrole cycle in the molecule. All NMR spectra of the synthesized compounds are given in the Supplementary Materials.

Hydrazones **7a**–**c** were prepared by the condensation of acid hydrazide **4** with benzaldehyde, 4-methoxybenzaldehyde, and 4-dimethylaminobenzaldehyde in refluxing ethanol (**a**,**c**) or a mixture of ethanol and 1,4-dioxane (1:2). In the reaction with itaconic acid, the hydrazide that has the amine group can readily undergo autocatalyzed intramolecular amidation–cyclization reaction to yield a stable 5-membered *N*-substituted pyrrolidinone cycle. The reaction was carried out in water at reflux for 15 h. Multiplets in the ranges of 2.50–2.90 (COCH_2_), 3.20–3.40 (CH) 3.56–3.72 (NCH_2_), and 3.87–4.18 (NCH_2_) ppm, integrated for 10 protons in total in the ^1^H NMR spectrum as well as double sets of the resonances of carbons of the CO*C*H_2_, CH, NCH_2_ fragments in the ^13^C NMR spectra of compound **8** approve the presence of two pyrrolidinone rings.

For the synthesis of the target benzoylhydrazine derivative **9**, hydrazide **4** was reacted with benzoyl chloride in dichloromethane at reflux for 10 min. The product **9** from the reaction mixture was isolated in 54% yield. The formation of the –CONHNHCOPh– fragment was approved by the presence of two singlets at 10.29 (NH) and 10.47 (NH) ppm, and the multiplet was integrated for nine protons of the two aromatic rings in the interval of 7.35–8.08 ppm.

To obtain a compound containing two hydrazinocarbonyl fragments, the reaction was carried out in hydrazine monohydrate using a 17 excess. The target product was obtained in a 54% yield.

The comparison of the spectra of compounds **4** and **10** demonstrated some differences that led to the easy identification of their specific structures. In the ^1^H NMR spectrum of compound **4**, the singlet at 3.82 ppm (^13^C, 51.99 ppm), integrated for three protons, shows the presence of the methoxy group, and the singlet at 4.35 ppm integrated for two protons proves the presence of the amino group, while in the ^1^H NMR of dihydrazide **10**, the signal of methoxy group is absent, and the broad singlet at 4.39 ppm is integrated for four protons, which proves the presence of two amino groups in the molecule. 

Hydrazones **11a**,**b** were obtained by the condensation of dihydrazide **10** with aromatic aldehydes. The reaction was carried out in the mixture of 2-propanol and 1,4-dioxane (ratio of 1:1.7) at reflux for 12 (**a**) or 11 (**b**) h, and products from the reaction mixtures were separated in 77% and 88% yields, respectively. 

The synthesized hydrazones **7** and **11** possess amide and azomethine groups in their structures. Based on the experimental and theoretical studies presented in literature [73], it can be stated that due to the presence of the amide fragment and the restricted rotation around the CO−NH bond, the hydrazones exist in DMSO solutions as a mixture of *Z/E* rotamers in which the *Z* rotamer predominates. The clearest proof of the existence of conformers produced due to the presence of the CONH fragment was the discovery of the two sets of resonances of the NH group in the low-field region of the ^1^H NMR spectra recorded in DMSO-*d*_6_, where a stronger-field side signal was related to the resonance of the rotamer with the *Z* structure.

The existence of the mixtures of stereoisomers relative to the CH=N structural fragment of the molecules was also found in the ^1^H NMR spectra of the monosubstituted hydrazones **7** and **11**. Based on the studies described in the academic literature [73], as well as the data of the spectra of these compounds, we can conclude that the produced mixtures of stereoisomers with the *Z-*isomer predominated.

The interaction of dihydrazide **10** with phenyl isothiocyanate in refluxing methanol led to the formation of thiosemicarbazide **12**, which then under the action of 4% sodium hydroxide at reflux for 6 h and subsequent acidification of the mixture with dilute hydrochloric acid (1:1) to pH 2 afforded heterocyclic compound **13** with two 4-phenyl-5-thioxo-1,2,4-triazole moieties in the structure. Resonances in the interval of 9.39–9.75 (4H, 2N*H*N*H*CO) as well as 10.12 and 10.40 (2H, 2NH) ppm in the ^1^H NMR spectrum of compound **12** is clear evidence for the formation of the –CONHNHCSNH– moiety. Cyclodehydration of this fragment in the presence of a strong base led to the 1,2,4-triazole **13** formation, which was confirmed by the absence of thiosemicarbazide-specific spectral lines and the observation of a decrease and downfield shift of the NH resonances (^1^H, 13.87 ppm). 

**Scheme 2:**
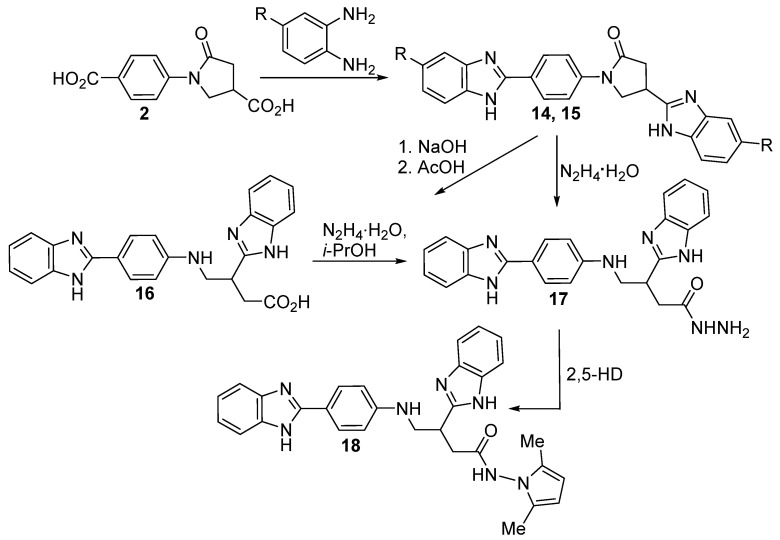
Synthesis of benzimidazole derivatives **14**–**18**. **14**, R = H; **15**, R = CH_3_.

Knowing the wide range of applications of the biological properties of benzimidazole derivatives in various fields, including medicine, pharmacy, optics, and others, we decided to synthesize compound **14** to have two benzimidazole moieties in its structure (Scheme 2). Five methods to achieve this goal were used. The reaction conditions and yields of the obtained product are given in Table 1.

The melting of carboxylic acid **2** with benzene-1,2-diamine at 170 °C and then at 230 °C gave the target compound a 51% yield. The condensation of benzene-1,2-diamine with acid **2** in 15% hydrochloric acid (by the Phillips method), at reflux yielded the desired product **14**, but the process took 96 h, and the product obtained in only a 8% yield. For this reason, we tried a more modern method using microwaves, where the reaction mixture was exposed to microwave irradiation (140 W) for 15 min under solvent-free conditions. Benzimidazole **14** was obtained in a 40% yield. The reaction in 2-propanol with ammonium chloride as a catalyst did not produce the expected yield of the benzimidazole. The yield of bisbenzimidazole **14** was found to be only 12%. The most efficient method for the preparation of compound **14** appeared to be condensation of dicarboxylic acid **2** with *o*-phenylenediamine in polyphosphoric acid (PPA) at 120 °C for 6 h. The product was separated in 97% yield. The last-mentioned method was used to obtain methylbenzimidazole derivative **15**.

The pyrrolidinone ring of compound **14** was readily decyclized under alkaline hydrolysis conditions by refluxing it in an aqueous 20% sodium hydroxide solution for 2 h. The NMR spectra of the obtained product **16** showed chemical shifts characteristic to the open-chain structure in comparison with the initial compound **14**.

The esterification of amino acid **16** with methanol in the presence of a catalytic amount of sulfuric acid was unsuccessful when the action of a strong acid led to the cyclization of the butanoic fragment to the initial pyrrolidinone ring. Therefore, to prepare acid hydrazide, we had to choose another synthesis route. Butanoic acid hydrazide **17** was obtained directly from pyrrolidinone derivatives **14** and its decyclized product—butanoic acid **16.** The interaction of butanoic acid **16** with hydrazine monohydrate proceeded successfully under mild conditions, i.e., heating the reaction mixture in 2-propanol at reflux for 20 h afforded acid hydrazide **17**. However, to obtain it from the pyrrolidinone derivative **14**, tightened reaction conditions were needed. Therefore, the reaction was carried out in excess hydrazine monohydrate at reflux for 6 h. The ^1^H and ^13^C NMR spectra of **17** confirmed the open-chain compound. In the NMR spectra of **17,** the triplet at 6.34 ppm was ascribed to the NH proton, the singlets at 4.43 (NH_2_, ^1^H) and 9.12 (N*H*NH_2_, ^1^H), and the spectral line at 169.90 (C=O, ^13^C) approved the formed acid hydrazide moiety. 

The condensation of hydrazide **17** with hexane-2,5-dione was investigated. As expected, the reaction in 2-propanol at reflux, with the presence of a catalytic amount of hydrochloric acid, afforded 2,5-dimethylpyrrole derivative **18**. The singlets at 1.62 and 1.95 (CH_3_) and the doublets at 5.52, 5.57 (C=CH) ppm in the ^1^H NMR spectrum, in addition to resonances at 10.50, 11.00, and 102.76, 102.81 ppm of the corresponding groups in the ^13^C NMR spectrum, prove the presence of the 2,5-dimethylpyrrole fragment.

Benzimidazoles play an important role in modern medicinal chemistry by being an important pharmacophore. With this notion, it is important to develop a large amount of structurally diverse benzimidazoles with potential medicinal properties.

**Scheme 3:**
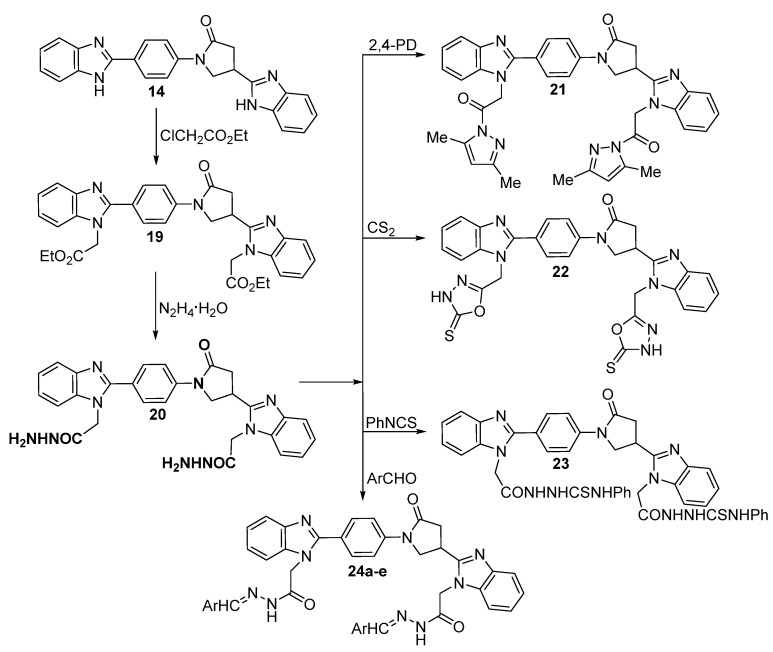
Synthesis of bisbenzimidazole derivatives **19**–**24**. **24 a**, Ar = C_6_H_5_; **b**, Ar = 4-O_2_N-C_6_H_4_; **c**, Ar = 4-F-C_6_H_4_; **d**, Ar = 3-Cl-C_6_H_4_; **e**, Ar = 2,3-di(H_3_CO)-C_6_H_3_.

For this purpose, the functionalization of benzimidazole **14** was performed (Scheme 3). The functionalization at nitrogen is perhaps the most common, and therefore it was chosen for the investigation. Initially, N-substituted benzimidazole derivative **19** was synthesized by the alkylation of bisbenzimidazole **14** with ethyl chloroacetate in acetone in the presence of potassium carbonate and a catalytic amount of TBAI (tetrabutylammonium iodide). The reaction was carried out at reflux for 20 h. The obtained ethyl ester **19** further was applied for the preparation of hydrazide **20**. The structures of the synthesized compounds **19** and **20** were determined by spectral methods and chemical transformations.

The refluxing of **19** with hydrazine monohydrate in 1,4-dioxane for 18 h led to the formation of compound **20** containing two 2-hydrazinyl-2-oxoethyl moieties, whose presence is confirmed by the singlets at 4.49 (2NH_2_, ^1^H), 4.87, 4.95, 5.24, and 5.32 (2NCH_2_CO, ^1^H), 8.83, 8.88, 9.60, and 9.65 (2NH, ^1^H), as well as by the resonance lines at 166.01 and 166.34 (2CONH, ^13^C) ppm.

Carbonyl compounds are frequently used as derivatization agents. The condensation of acid hydrazide with diketone pentane-2,4-dione gave 3,5-dimethylpyrazole derivatives **21** a 82% yield. The reaction of **21** was performed for 5 h in refluxing 2-propanol and in the presence of a catalytic amount of hydrochloric acid. The reaction product was isolated from the reaction mixture by diluting it with water. The data of the ^1^H and ^13^C NMR spectroscopic techniques and elemental analysis confirmed the proposed structures of the synthesized pyrazole **21**.

Oxadiazole derivative **22** was obtained by the ring closure reaction of the hydrazide **20** with carbon disulfide in alkaline medium obtained by using potassium hydroxide, which was dissolved in methanol, and then CS_2_ was added dropwise to the cooled solution. After a thorough stirring for 15 min, the required amount of hydrazide was added, and the obtained reaction mixture was refluxed for 12 h. The acidifying of the aqueous solution of the reaction mixture with hydrochloric acid to pH 1 afforded the desired derivative **22** with two 1,3,4-oxadiazole moieties in the molecule. The signals in the NMR spectra of the compound were an exact match of the protons and carbon atoms of the obtained structure.

Derivative **23** with two thiosemicarbazide moieties in the molecule was obtained in the reaction of hydrazide **20** with phenyl isothiocyanate. The reaction was performed in methanol at reflux for 6 h, and the obtained hydrazinecarbothioamine **23** was then applied for the synthesis of triazolethione using a method of cyclization in basic conditions. Cyclization was performed in an aqueous 4% sodium hydroxide solution in order to prevent 5-oxopyrrolidine ring breakage with the subsequent acidification of the reaction mixture with dilute hydrochloric acid (1:1) to pH 2. However, the process failed, and efforts to separate the cyclized product were unsuccessful. The spectral data (NMR, IR, and elemental analysis) of thiosemicarbazide **23** were in full agreement with the proposed structure. The multiplet in the range of 9.48–10.04 (4NH) ppm and the singlets at 10.51 and 10.58 (2NH) ppm in the ^1^H NMR spectrum for **23** as well as additional peaks in the interval of 7.08–7.95 ppm prove the presence of the CONHNHCSNHPh fragment.

The hydrazones **24** were prepared by the condensation of hydrazide **20** with the corresponding aromatic aldehyde (benzaldehyde (**a**), 4-nitro- (**b**), 4-fluoro- (**c**), 3-chloro- (**d**) and 2,3-dimethoxybenzaldehyde (**e**)) in a molar ration of 1:7.5. The reactions were carried out by heating the mixtures at reflux for 5 h, except in case **b** when the reaction proceeded for 8 h. The products were separated in good to excellent yields in the range of 77%–94%.

When ^1^H-NMR spectra of compounds **24** were observed, two sets of signals of the protons at different ppm were seen. This is because of the compounds, which have an arylidene hydrazide structure. The restricted rotation around the CO−NH bond causes the formation of a mixture of *Z/E* rotamers, whereas the presence of a double bond of CH=N influences the formation of geometric isomers, which are clearly visible in the spectra of these compounds [74]. The ratio in each case was calculated by using ^1^H-NMR data, and they are as follows: 0.75:0.25 for **a**–**c**, **e** and 0.8:0.2 for **d**.

### 2.2. The Antimicrobial Activity of the Synthesized Compounds

In this study, we aimed to synthesize a series of novel benzimidazole derivates bearing ethyl ester, hydrazide, 3,5-dimethylpyrazole, 2,5-dimethylpyrrole, oxadiazole, thiosemicarbazide, and hydrazone moieties and to investigate their in vitro antimicrobial properties against a series of Gram-positive and Gram-negative bacterial pathogens.

The antimicrobial properties of synthesized compounds **3**–**24** were investigated against *Staphylococcus aureus, Listeria monocytogenes, Bacillus cereus, Escherichia coli, Pseudomonas aeruginosa*, and *Salmonella enteritidis* (Supplementary Table S1). The minimal inhibitory concentration (MIC) was evaluated by the broth dilution method, while the minimal bactericidal concentration (MBC) was determined by plating.

Compounds **3***–***24** demonstrated acceptable antimicrobial activity against Gram-positive microorganisms, suggesting the possible existence of Gram-positive bacteria-selective targets for compounds **3**, **5**, **14***–***17**, **19**, and **20**, which bear dimethyl ester, 3,5-dimethylpyrazole, bisbenzimidazole, bis 5(6)-methylbenzimidazole, *γ*-amino acid and its hydrazide, diethyl ester, and dihydrazide fragments, respectively. The antimicrobial activity was highly structure-depended on and was mostly bactericidal at near-MIC concentration (Table 2). The most promising compounds demonstrated near-MIC bactericidal activity, therefore further showing importance of the abovementioned scaffold as novel antimicrobials targeting Gram-positive bacterial pathogens.

The results obtained in this study showed that all tested methyl derivatives **3**–**9**, dihydrazide **10,** and its derivatives **12** and **13** demonstrated moderate antimicrobial activity on all tested bacteria strains (Table 2). Moreover, hydrazones **11a**,**b** did not demonstrate antimicrobial activity on neither Gram-positive nor Gram-negative bacteria. Hydrazone **11b** harboring 4-chlorobenzylidene fragment demonstrated weak but selective bactericidal activity on *S. aureus* (250 µg/mL) but not on other Gram-positive or Gram-negative organisms (Table 2).

Diester **3** and pyrazole derivative **5** exhibited good bactericidal activity (MIC and MBC of 31.25 µg/mL) against *B. cereus*. Interestingly, compounds **3** and **5** did not show a good antimicrobial activity against other Gram-positive organisms, suggesting the possible presence of *B. cereus*-specific targets of compound **5** (Table 2).

Benzimidazole **14**, methylbenzimidazole **15**, *γ*-amino acid **16**, and oxadiazole **22** demonstrated broad-spectrum antimicrobial activity that targets both Gram-negative and Gram-positive microorganisms (Table 2). Compound **16** demonstrated the highest antimicrobial activity against all tested strains, suggesting the important role of *γ*-amino acid moiety for biological activity.

Furthermore, in this study, the bactericidal activity of hydrazide **17** and diester **19** bactericidal activity on *S. aureus* was comparable to ampicillin (62.5 µg/mL). The incorporation of *γ*-amino acid moiety in compound **16** and 4-(nitrobenzylidene)hydrazinyl fragment in **24b** resulted in a compound with good activity against Gram-negative organisms (MIC and MBC at 31.25 µg/mL). In this study, compound **16** bearing *γ*-amino acid moiety demonstrated the most potent antimicrobial activity against a broad spectrum of microorganisms, demonstrating the importance of the abovementioned fragment as an antibacterial pharmacophore.

Interestingly, benzimidazoles **14** and **15** were an exception in this assay and had an exclusive activity against *L. monocytogenes*, although no activity was observed when tested against *S. aureus* or *B. cereus.* Compound **14** demonstrated slightly better, near-MIC bactericidal activity (MBC 15.62 µg/mL) (Table 2). Benzimidazole **15** bearing 5(6)-methyl moiety showed one dilution higher MBC (31.25 µg/mL), suggesting that the 5(6)-methyl moiety is important for bactericidal activity against *L. monocytogenes.*

The investigations of structure-activity based relationships revealed some evident facts that changes in the 1-phenyl-5-oxopyrrolidine backbone by an incorporation of benzimidazole moieties greatly affect the biological properties of the compounds. The data presented in Table 2 demonstrate that benzimidazole **14** shows broad-spectrum antimicrobial activity, which was most evident when tested against *L. monocytogenes*. The antimicrobial activity of compound bearing a nitro group (**24b**) in the benzene ring was confirmed in this study when stronger antibacterial properties against the *E. coli* and *P. aeruginosa* strains were seen in comparison to other hydrazones **24**.

The results generated during this study are expected to be a foundation for the development of novel 1-phenyl-5-oxopyrrolidine-based antimicrobials. Further studies are needed to better understand the mechanism of antimicrobial activity as well as to generate more potent antimicrobial compounds based on the 1-phenyl-5-oxopyrrolidine nucleus. 

## 3. Materials and Methods

### 3.1. Synthesis

Reagents and solvents were obtained from Sigma-Aldrich (St. Louis, MO, USA) and used without further purification. The reaction course and purity of the synthesized compounds were monitored by TLC using aluminum plates precoated with Silica gel with F254 nm (Merck KGaA, Darmstadt, Germany). Melting points were determined with a B-540 melting point analyzer (Büchi Corporation, New Castle, DE, USA) and were uncorrected. NMR spectra were recorded on a Brucker Avance III (400, 101 MHz) spectrometer. Chemical shifts were reported in (δ) ppm relative to tetramethylsilane (TMS) with the residual solvent as internal reference ([D6]DMSO, δ = 2.50 ppm for ^1^H and δ = 39.5 ppm for ^13^C). Data were reported as follows: chemical shift, multiplicity, coupling constant (Hz), integration, and assignment. IR spectra (ν, cm^−1^) were recorded on a Perkin–Elmer Spectrum BX FT–IR spectrometer using KBr pellets. Mass spectra were obtained on a Bruker maXis UHRTOF mass spectrometer with ESI ionization. Elemental analyses (C, H, N) were conducted using the Elemental Analyzer CE-440; their results were found to be in good agreement (±0.3%) with the calculated values.

*1-(4-Carboxyphenyl)-5-oxopyrrolidine-3-carboxylic acid* (**2**)**:** A mixture of itaconic acid (65.05 g, 0.5 mol) and *p*-aminobenzoic acid **1** (34.3 g, 0.25 mol) was refluxed in water (150 mL) for 6 h, then cooled down; the formed crystalline precipitate was filtered off, washed with water, 2-propanol, and recrystallized from 2-propanol to give the title compound **2** (white solid, yield 45 g, 72%, m. p. 290 °C (decomp.)).

^1^H-NMR (400 MHz, DMSO-*d*_6_): δ = 2.69–2.87 (m, 2H, COCH_2_), 3.30–3.44 (m, 1H, CH), 3.96–4.11 (m, 2H, NCH_2_), 7.78 (d, *J* = 8.7 Hz, 2H, H_ar_), 7.94 (d, *J* = 8.7 Hz, 2H_ar_), 12.83 (br s, 2H, 2OH) ppm.

^13^C-NMR (101 MHz, DMSO-*d*_6_): δ = 35.08 (CO*C*H_2_), 35.38 (CH), 49.85 (NCH_2_), 118.49, 125.76, 130.18, 142.91 (C_ar_), 166.89, 172.54, 174.10 (3C=O) ppm. 

IR (KBr): ν_max_ = 3121 (OH), 1706, 1671 (3C=O) cm^−1^.

MS (APCI+, 25 V) *m*/*z*, %: 272 (100) [M + Na]^+^.

Calcd. for C_12_H_11_O_5_N, %: C 57.83; H 4.45; N 5.62. Found, %: C 57.98; H 4.52; N 5.68.

*Methyl 1-[4-(methoxycarbonyl)phenyl]-5-oxopyrrolidine-3-carboxylate* (**3**)**:** To a solution of carboxylic acid **2** (31.13 g, 0.125 mol) in methanol (350 mL), concentrated sulfuric acid (12.5 mL) was added dropwise, and the mixture was heated at reflux for 4 h. The solvent was then evaporated under reduced pressure, and the residue neutralized with 10% sodium carbonate solution to pH 6–7. After cooling, the obtained solid was filtered off, washed with plenty of water and 2-propanol, and recrystallized from 2-propanol to give the title compound **3** (white solid, yield 23.5 g, 68%, m. p. 142–143 °C).

^1^H-NMR (400 MHz, DMSO-*d*_6_): δ = 2.72–2.91 (m, 2H, COCH_2_), 3.42–3.55 (m, 1H, CH), 3.69 (s, 3H, OCH_3_), 3.84 (s, 3H, OCH_3_), 3.99–4.16 (m, 2H, NCH_2_), 7.82 (d, *J* = 8.8 Hz, 2H, H_ar_), 7.97 (d, *J* = 8.8 Hz, 2H, H_ar_) ppm**.**

^13^C-NMR (101 MHz, DMSO-*d*_6_): δ = 34.80 (CO*C*H_2_), 35.14 (CH), 49.56 (NCH_2_), 51.96 (OCH_3_), 52.19 (OCH_3_), 118.52, 124.54, 130.00, 143.11 (C_ar_), 165.72, 172.32, 172.91 (3C=O) ppm. 

IR (KBr): ν_max_ = 1735, 1707 (3C=O) cm^−1^.

MS (APCI+, 25 V) *m*/*z*, %: 300 (100) [M + Na]^+^.

Calcd. for C_14_H_15_O_5_N, %: C 60.64; H 5.45; N 5.05. Found, %: C 60.88; H 5.50; N 5.14.

*Methyl 4-[3-(hydrazinocarbonyl)-5-oxopyrrolidin-1-yl]benzoate* (**4**)**:** To a boiling mixture of methyl ester **3** (13.86 g, 0.05 mol) and 2-propanol (50 mL), hydrazine monohydrate (7.5 g, 0.15 mol) was added, and the mixture was heated at reflux for 1 h. After completion of the reaction (TLC), the mixture was cooled to room temperature; the formed precipitate was filtered off, washed with water, 2-propanol, and hexane, and recrystallized from 2-propanol to give the title compound **4** (white solid, yield 12.06 g, 87%, m. p. 198–199 °C).

^1^H-NMR (400 MHz, DMSO-*d*_6_): δ = 2.61–2.82 (m, 2H, COCH_2_), 3.09–3.26 (m, 1H, CH), 3.82 (s, 3H, OCH_3_), 3.84–3.91 (m, 1H, NCH_2_), 3.98–4.07 (m, 1H, NCH_2_), 4.35 (s, 2H, NH_2_), 7.81 (d, *J* = 8.4 Hz, 2H, H_ar_), 7.95 (d, *J* = 8.4 Hz, 2H, H_ar_), 9.30 (s, 1H, NH) ppm.

^13^C-NMR (101 MHz, DMSO-*d*_6_): δ = 33.94 (CO*C*H_2_), 35.93 (CH), 50.58 (NCH_2_), 51.99 (OCH_3_), 118.41, 124.40, 130.02, 143.25 (C_ar_), 165.77, 171.44, 172.91 (3C=O) ppm. 

IR (KBr): ν_max_ = 3280, 3308 (NH, NH_2_), 1724, 1685, 1637 (3C=O), 1282 (OCH_3_) cm^−1^.

MS (APCI+, 25 V) *m*/*z*, %: 300 (100) [M + Na]^+^.

Calcd. for C_13_H_15_O_4_N_3_, %: C 56.32; H 5.42; N 15.16. Found, %: C 56.48; H 5.52; N 15.22.

*Methyl 4-(4-(3,5-dimethyl-1H-pyrazole-1-carbonyl)-2-oxopyrrolidin-1-yl)benzoate* (**5**)**:** To a solution of hydrazide **4** (1.11 g, 4 mmol) in ethanol (8 mL), 2,4-pentanedione (1 g, 10 mmol) was added, and the mixture was heated at reflux for 2 h; it then cooled down, and the formed precipitate was filtered off, washed with ethanol, and recrystallized from methanol to give the title compound **5** (white solid, yield 0.56 g, 41%, m. p. 161–162 °C). 

^1^H-NMR (400 MHz, DMSO-*d*_6_): δ = 2.21, 2.48 (2s, 6H, 2CH_3_), 2.84–2.99 (m, 2H, COCH_2_), 3.82 (s, 3H, OCH_3_), 4.04–4.28 (m, 2H, NCH_2_), 4.44–4.54 (m, 1H, CH), 6.23 (s, 1H, C=CH−C), 7.82 (d, *J* = 8.7 Hz, 2H, H_ar_), 7.96 (d, *J* = 8.7 Hz, 2H, H_ar_) ppm. 

^13^C-NMR (101 MHz, DMSO-*d*_6_): δ = 13.58, 14.03 (2CH_3_), 35.27 (CH + CO*C*H_2_), 49.96 (NCH_2_), 51.97 (OCH_3_), 111.61, 118.58, 124.53, 130.01, 143.12, 143.91, 152.19 (C_ar_), 165.73, 172.39, 172.43 (3C=O) ppm. 

IR (KBr): ν_max_ = 1729 (3C=O), 1276 (OCH_3_) cm^−1^.

MS (APCI+, 25 V) *m*/*z*, %: 364 (100) [M + Na]^+^. 

Calcd. for C_18_H_19_N_3_O_4_, %: C 63.33; H 5.61; 12.31. Found, %: C 63.49; H 5.75; N 12.47. 

*Methyl 4-(4-((2,5-dimethyl-1H-pyrrol-1-yl)carbamoyl)-2-oxopyrrolidin-1-yl)benzoate* (**6**)**:** To a solution of hydrazide **4** (3,48 g, 12.5 mmol) in ethanol (15 mL), 2,5-hexanedione (1.85 mL, 15.8 mmol) and glacial acetic acid (2 mL) were added by stirring, and the mixture was heated at reflux for 1,5 h. Then the reaction mixture was cooled down, and the formed crystalline solid was filtered off, washed with ethanol and ether, and recrystallized from methanol to give the title compound **6** (yellowish solid, yield 2.11 g, 47.5%, m. p. 147–148 °C).

^1^H-NMR (400 MHz, DMSO-*d*_6_): δ = 2.00 (s, 6H, 2CH_3_), 2.74–3.01 (m, 2H, COCH_2_), 3.46–3.54 (m, 1H, CH), 3.84 (s, 3H, OCH_3_), 3.98–4.22 (m, 2H, NCH_2_), 5.65 (s, 2H, 2C=CH), 7.85 (d, *J* = 8.8 Hz, 2H, H_ar_), 7.97 (d, *J* = 8.8 Hz, 2H, H_ar_), 10.94 (s, 1H, NH) ppm.

^13^C-NMR (101 MHz, DMSO-*d*_6_): δ = 10.94, 10.96 (2CH_3_), 33.98 (CO*C*H_2_), 35.87 (CH), 50.21 (NCH_2_), 51.99 (OCH_3_), 103.10, 118.53, 124.55, 126.74, 130.05, 143.16 (C_ar_), 165.75, 171.72, 172.41 (3C=O) ppm. 

IR (KBr): ν_max_ = 3264 (NH), 1714, 1702, 1671 (3C=O), 1286 (OCH_3_) cm^−1^.

MS (APCI+, 25 V) *m*/*z*, %: 378 (100) [M + Na]^+^.

Calcd. for C_19_H_21_N_3_O_4_, %: C 64.21; H 5.96; N 11.82. Found, %: C 64.32; H 6.03; N 11.76. 

General procedure for the preparation of hydrazones **7a**–**c**: 

To the boiling solution of hydrazide **4** (1.11 g, 4 mmol) in ethanol (50 mL) (**a**,**c**) or ethanol:1,4-dioxane (5:10 mL) (**b**) the corresponding aromatic aldehyde [benzaldehyde (6 mmol), 4-methoxybenzaldehyde (5 mmol), 4-dimethylaminobenzaldehyde (6 mmol)] was added, and the reaction mixture was heated at reflux for 3.5 (**a**,**c**), 2.5 (**b**) h. Then the mixture was cooled down; the formed solid was filtered off, washed with 2-propanol, and recrystallized from 2-propanol to give the title compound **7a** (white solid, yield 0.79 g, 54%, m. p. 201–203 °C), **7b** (white solid, yield 1.33 g, 84%, m. p. 230–231 °C), and **7c** (yellowish solid, yield 1.22 g, 70%, m. p. 199–201 °C).

*Methyl 4-(4-(2-benzylidenehydrazine-1-carbonyl)-2-oxopyrrolidin-1-yl)benzoate* (**7a**)**:** ^1^H-NMR (400 MHz, DMSO-*d*_6_): δ = *Z/E* 2.78–2.95 (m, 2H, COCH_2_), 3.37–3.43 (m, 0.3H, CH, overlaps with the water peak), 3.83 (s, 3H, OCH_3_), 4.01–4.24 (m, 0.7H, CH + 2H, NCH_2_), 7.40–7.47 (m, 3H, H_ar_), 7.69–7.74 (m, 2H, H_ar_), 7.85 (d, *J* = 8.7 Hz, 2H, H_ar_), 7.93–8.00 (m, 2H, H_ar_), 8.05 (s, 0.7H, CH=N), 8.24 (s, 0.3H, CH=N), 11.62, 11.69 (2s, 1H, NH) ppm.

IR (KBr): ν_max_ = 3254 (NH), 1719, 1677 1658 (3C=O), 1289 (OCH_3_) cm^−1^.

MS (APCI+, 25 V) *m*/*z*, %: 366 (100) [M + H]^+^.

Calcd. for C_20_H_19_N_3_O_4_, %: C 65.74; H 5.24; N 11.50. Found, %: C 65.65; H 5.22; N 11.46.

*Methyl 4-(4-(2-(4-methoxybenzylidene)hydrazine-1-carbonyl)-2-oxopyrrolidin-1-yl)benzoate* (**7b**)**:** ^1^H-NMR (400 MHz, DMSO-*d*_6_): δ = *Z/E* 2.77–2.93 (m, 2H, COCH_2_), 3.78 (s, 3H, OCH_3_), 3.82 (s, 3H, OCH_3_), 3.79–4.26 (m, 1H, CH + 2H, NCH_2_), 6.96–7.01 (m, 2H, H_ar_), 7.64 (d, *J* = 8.5 Hz, 2H, H_ar_), 7.84 (d, *J* = 8.7 Hz, 2H, H_ar_), 7.93–8.03 (m, 2H, H_ar_ + 0.6H, N=CH), 8.41 (s, 0.4H, N=CH), 11.47, 11.54 (2s, 1H, NH) ppm.

IR (KBr): ν_max_ = 3240 (2NH), 1717, 1680, 1653 (3C=O) cm^−1^.

MS (APCI+, 25 V) *m*/*z*, %: 396 (100) [M + H]^+^.

Calcd. for C_21_H_21_N_3_O_5_, %: C 63.79; H 5.35; N 10.63. Found, %: C 63.69; H 5.44; N 10.67.

*Methyl 4-(4-(2-(4-(diethylamino)benzylidene)hydrazine-1-carbonyl)-2-oxopyrrolidin-1-yl)benzoate* (**7c**)**:** ^1^H-NMR (400 MHz, DMSO-*d*_6_): δ = *Z/E* 1.08 1.10 (2t, *J* = 7.0 Hz, 6H, 2CH_3_), 2.72–2.93 (m, 2H, COCH_2_), 3.34–3.41 (m, 4H, 2C*H*_2_CH_3_ + 0.4H, CH), 3.83 (s, 3H, OCH_3_), 3.96–4.22 (m, 2H, NCH_2_ + 0.6H, CH), 6.55–6.80 (m, 2H, H_ar_), 7.48 (d, *J* = 8.7 Hz, 2H, H_ar_), 7.80–8.01 (m, 4H, H_ar_ + 0.6H, N=CH), 8.05 (s, 0.4H, N=CH), 11.28, 11.32 (2s, 1H, NH) ppm.

IR (KBr): ν_max_ = 3248 (2NH), 1718, 1702, 1663 (3C=O) cm^−1^.

MS (APCI+, 25 V) *m*/*z*, %: 437 (100) [M + H]^+^.

Calcd. for C_24_H_28_N_4_O_4_, %: C 66.04; H 6.47; N 12.84. Found, %: C 66.01; H 6.48; N 12.74.

*1-(1-(4-(Methoxycarbonyl)phenyl)-5-oxopyrrolidine-3-carboxamido)-5-oxopyrrolidine-3-carboxylic acid* (**8**)**:** A mixture of hydrazide **4** (1.11 g, 4 mmol), itaconic acid (0.62 g, 4.8 mmol), and water (15 mL) was refluxed for 15 h and then cooled down; the formed precipitate was filtered off, washed with plenty of water and hexane, and recrystallized from 2-propanol to give the title compound **8** (yellowish solid, yield 1.23 g, 79%, m. p. 249–251 °C).

^1^H-NMR (400 MHz, DMSO-*d*_6_): δ = 2.50–2.90 (m, 4H, 2COCH_2_), 3.20–3.40 (m, 2H, 2CH), 3.56–3.72 (m, 2H, NCH_2_), 3.83 (s, 3H, OCH_3_), 3.87–4.18 (m, 2H, NCH_2_), 7.82 (d, *J* = 8.8 Hz, 2H, H_ar_), 7.97 (d, *J* = 8.8 Hz, 2H, H_ar_), 10.40 (s, 1H, NH), 12.82 (br. s, 1H, OH) ppm.

^13^C-NMR (101 MHz, DMSO-*d*_6_): δ = 31.26, 33.56, 34.13, 35.62, 49.64, 50.19, 50.21 (2CO*C*H_2_, 2CH, 2NCH_2_), 52.01 (OCH_3_), 118.51, 124.52, 130.06, 143.16 (C_ar_), 165.76, 170.90, 171.29, 172.52 (4C=O), 174.02 (COOH) ppm.

IR (KBr): ν_max_ = 3410 (OH), 3275 (NH), 1727, 1702, 1684, 1666 (5C=O), 1286 (OCH_3_) cm^−1^.

Calcd. for C_18_H_19_N_3_O_7_, %: C 55.53; H 4.92; N 10.79. Found, %: C 55.47; H 4.99; N 10.92.

*Methyl 4-(4-(2-benzoylhydrazine-1-carbonyl)-2-oxopyrrolidin-1-yl)benzoate* (**9**)**:** To the boiling solution of hydrazide (5 g, 1.8 mmol) **4** in dichloromethane (100 mL), benzoyl chloride was added dropwise and the reaction mixture was heated at reflux for 10 min. It was then cooled down, and the formed precipitate was filtered off, washed with dichloromethane and ethanol, and recrystallized from methanol to give the title compound **9** (yellowish solid, yield 3.7 g, 54%, m. p. 239–241 °C).

^1^H-NMR (400 MHz, DMSO-*d*_6_): δ = 2.66–2.94 (m, 2H, COCH_2_), 3.41–3.49 (m, 1H, CH), 3.83 (s, 3H, OCH_3_), 3.91–4.22 (m, 2H, NCH_2_), 7.35–8.08 (m, 9H, H_ar_), 10.29, 10.47 (2s, 2H, 2NH) ppm.

^13^C-NMR (101 MHz, DMSO-*d*_6_): δ = 33.71, 33.81, 35.51, 35.80, 50.07, 50.46 (CO*C*H_2_, CH, NCH_2_), 52.02 (OCH_3_), 118.48, 118.52, 124.49, 124.57, 127.44, 128.50, 130.07, 131.90, 132.31, 143.10, 143.22 (C_ar_), 165.55, 165.74, 165.77, 171.62, 171.91, 172.38, 172.71 (4C=O) ppm.

IR (KBr): ν_max_ = 3206 (2NH), 1718, 1682, (4C=O), 1281 (OCH_3_) cm^−1^.

MS (APCI+, 25 V) *m*/*z*, %: 382 (100) [M + H]^+^.

Calcd. for C_20_H_19_N_3_O_5_, %: C 62.99; H 5.02; N 11.02. Found, %: C 62.87; H 5.16; N 11.12.

*1-(4-(Hydrazinecarbonyl)phenyl)-5-oxopyrrolidine-3-carbohydrazide* (**10**)**:** A mixture of methyl ester **3** (13.85 g, 0.05 mol) and hydrazine monohydrate (42.6 g, 0.85 mol) was heated at reflux for 2 h. After completion of the reaction (TLC), the mixture was cooled to room temperature, and the formed precipitate filtered off, washed with plenty of water and 2-propanol, and recrystallized from water to give the title compound **10** (white solid, yield 7.5 g, 54%, m. p. 225–226 °C).

^1^H-NMR (400 MHz, DMSO-*d*_6_): δ = 2.57–2.82 (m, 2H, COCH_2_), 3.03–3.32 (m, 1H, CH), 3.82–4.10 (m, 2H, NCH_2_), 4.39 (br. s, 4H, NH_2_), 7.73 (d, *J* = 8.7 Hz, 2H, H_ar_), 7.85 (d, *J* = 8.7 Hz, 2H, H_ar_), 9.31 (s, 1H, CONH), 9.72 (s, 1H, CONH) ppm.

^13^C-NMR (101 MHz, DMSO-*d*_6_): δ = 34.04 (CO*C*H_2_), 35.90 (CH), 50.61 (NCH_2_), 118.40, 127.63, 128.32, 141.47 (C_ar_), 165.41, 171.53, 172.60 (3C=O) ppm.

IR (KBr): ν_max_ = 3307, 3279, 3193, 3167 (2NH_2_, 2NH), 1687, 1635 (3C=O) cm^−1^.

MS (APCI+, 25 V) *m*/*z*, %: 300 (100) [M + Na]^+^.

Calcd. for C_12_H_15_O_3_N_5_, %: C 51.98; H 5.45; N 25.26. Found, %: C 52.04; H 5.53; N 25.29.

General procedure for the preparation of hydrazones **11a**, **b**: 

A mixture of dihydrazide **10** (0.5 g, 1.8 mmol), the corresponding aromatic aldehyde (10.8 mmol) and 2-propanol/1,4-dioxane (1:1.7) was heated at reflux for 12 (**a**) or 11 (**b**) h. Then the mixture was cooled down, and the formed solid filtered off and washed with 2-propanol, and recrystallized from 1,4-dioxane to give the title compound **11a** (yellow solid, yield 0.76 g, 77%, m. p. 295–296 °C and **11b** (white solid, yield 0.83 g, 88%, m. p. 298–300 °C).

*N-(4-nitrobenzylidene)-1-[4-[2-(4-nitrobenzylidenehydrazinocarbonyl)]phenyl]-5-oxopyrrolidine-3-carbohydrazide* (**11a**)**:** ^1^H-NMR (400 MHz, DMSO-*d*_6_): δ = *Z/E* 2.75–2.98 (m, 2H, COCH_2_), 3.38–3.48 (m, 0.35H, CH), 4.01–4.27 (m, 0.65H, CH + 2H, NCH_2_), 7.76–8.09 (m, 8H, H_ar_), 8.14 (s, 0.65H, CH=N), 8.16–8.32 (m, 4H, H_ar_), 8.33 (s, 0.35H, CH=N), 8.54 (s, 1H, CH=N), 11.89, 11.96 (2s, 1H, NH), 12.10 (s, 1H, NH) ppm.

IR (KBr): ν_max_ = 3433 (2NH), 1667, 1603 (3C=O) cm^−1^.

MS (APCI+, 25 V) *m*/*z*, %: 544(100) [M + H]^+^.

Calcd. for C_26_H_21_N_7_O_7_, %: C 57.46; H 3.89; N 18.04. Found, %: C 57.57; H 3.97; N 17.98.

*N-(4-chlorobenzylidene)-1-[4-[2-(4-chlorobenzylidenehydrazinocarbonyl)]phenyl]-5-oxopyrrolidine-3-carbohydrazide* (**11b**)**:** ^1^H-NMR (400 MHz, DMSO-*d*_6_): δ = *Z/E* 2.73–2.97 (m, 2H, COCH_2_), 3.36–3.44 (m, 0.35H, CH), 3.98–4.26 (m, 0.65H, CH + 2H, NCH_2_), 7.38–7.62 (m, 4H, H_ar_), 7.63–8.20 (m, 8H, H_ar_ + 0.65H, CH=N), 8.23 (s, 0.35H, CH=N), 8.45 (s, 1H, CH=N), 11.66, 11.74 (2s, 1H, NH), 11.88 (s, 1H, NH) ppm.

IR (KBr): ν_max_ = 3431, 3235 (2NH), 1679, 1655 (3C=O) cm^−1^.

MS (APCI+, 25 V) *m*/*z*, %: 523 (100) [M + H]^+^.

Calcd. for C_26_H_21_Cl_2_N_5_O_3_, %: C 59.78; H 4.05; N 13.41. Found, %: C 59.68; H 4.04; N 13.48.

*1-[[5-Oxo-1-(4-[(phenylcarbamoylthioamino)carbamoyl]pyrrolidin-3-carbonyl]-3-phenylthiocarbamide* (**12**)**:** To a solution of dihydrazide **10** (5 g, 18 mmol) in methanol (50 mL), phenyl isothiocyanate (9.73 g, 72 mmol) was added, and the mixture was heated at reflux for 5 h. After completion of the reaction, the mixture was cooled down, and the obtained crystalline solid was filtered off, washed with methanol and boiling water, and recrystallized from methanol to give the title compound **12** (white solid, yield 8.97 g, 97% m. p. 235–236 °C).

^1^H-NMR (400 MHz, DMSO-*d*_6_): δ = 2.56–2.76 (m, 2H, COCH_2_), 2.89–3.41 (m, 1H, CH + NCH_2_), 6.28–6.74 (m, 4H, H_ar_), 7.09–7.77 (m, 10H, H_ar_), 9.39–9.75 (m, 4H, 4NH), 10.12, 10.40 (2s, 2H, 2NH) ppm.

^13^C-NMR (101 MHz, DMSO-*d*_6_): δ = 33.39 (CH, CO*C*H_2_), 44.37 (NCH_2_), 111.02, 113.98, 115.79, 116.81, 119.46, 123.68, 124.50, 124.84, 124.88, 124.92, 125.13, 125.23, 125.87, 125. 91, 128.10, 128.84, 129.58, 139.01, 139.34, 151.41, 166.04, 171.80, 173.00, 180.06, 180.98 (C_ar_, 3C=O, 2C=S) ppm.

IR (KBr): ν_max_ = 3312 (NH), 1611 (3C=O), 1332 (C=S) cm^−1^.

MS (APCI+, 25 V) *m*/*z*, %: 548 (100) [M + H]^+^.

Calcd. for C_26_H_25_N_7_O_3_S_2_, %: C 57.02; H 4.60; N 17.90. Found, %: C 57.10; H 4.56; N 17.93.

*4-(4-Phenyl-5-thioxo-4,5-dihydro-1H-1,2,4-triazol-3-yl)-1-(4-(4-phenyl-5-thioxo-4,5-dihydro-1H-1,2,4-triazol-3-yl)phenyl)pyrrolidin-2-one* (**13**)**:** A mixture of thiosemicarbazide **12** (1.03 g, 2 mmol) and 4% sodium hydroxide solution (20 mL) was refluxed for 6 h. The reaction mixture was then cooled down, acidified, with diluted hydrochloric acid (1:1) to pH 2. The formed solid was filtered off, washed with water, and recrystallized from methanol to give the title compound **13** (white solid, yield 0.61 g, 60%, m. p. 320–322 °C). 

^1^H-NMR (400 MHz, DMSO-*d*_6_): δ = 2.67–2.88 (m, 2H, COCH_2_), 2.89–3.25 (m, 3H, NCH_2_ + CH), 6.81 (d, *J* = 8.4 Hz, 2H, H_ar_), 7.11–7.22 (m, 4H, H_ar_), 7.31–7.50 (m, 6H, H_ar_), 7.58–7.62 (m, 2H, Har), 13.87 (s, 2H, 2NH) ppm.

^13^C-NMR (101 MHz, DMSO-*d*_6_): δ = 26.83 (CH), 32.48 (CO*C*H_2_), 45.39 (NCH_2_), 110.84, 112.53, 127.94, 128.90, 129.33, 129.39, 129.44, 133.10, 122.14, 135.16, 148.80, 150.05, 150.05, 150.93, 152.46, 167.59, 167.73, 168.16 (C_ar_, C=O, 2C=S) ppm.

Calcd. for C_26_H_21_N_7_OS_2_, %: C 61.04; H 4.14; N 19.16. Found, %: C 61.13; H 4.10; N 19.12.

General procedure for the preparation of benzimidazoles **14** and **15**.

To a mixture of dicarboxylic acid **2** (5 g, 20 mmol) and benzene-1,2-diamine (**14**) or 4-methylbenzene-1,2-diamine (**15**) (52 mmol), polyphosphoric acid (15 g) was added dropwise, and the mixture was heated at 120 °C for 6 h. It was then cooled down and neutralized with 7% Na_2_CO_3_ to pH 9. The formed precipitate was filtered off, washed with plenty of water, and recrystallized from methanol to give the title compound **14** (white solid, yield 7.6 g, 97%, m. p. 215–216 °C) or compound **15** (light yellow solid, yield 7.67 g, 91%, m. p. 257–258 °C).

*3-(1H-benzimidazol-2-yl)-1-[4-(1H-benzimidazol-2-yl)phenyl]pyrrolidin-5-one* (**14**)**:** ^1^H-NMR (400 MHz, DMSO-*d*_6_): δ = 3.01–3.20 (m, 2H, COCH_2_), 4.01–4.11 (m, 1H, CH), 4.24–4.42 (m, 2H, NCH_2_), 7.10–7.24 (m, 4H, H_ar_), 7.48–7.69 (m, 4H, H_ar_), 7.91 (d, *J* = 8.9 Hz, 2H, H_ar_), 8.20 (d, *J* = 8.8 Hz, 2H, H_ar_), 12.50, 12.87 (2s, 2H, 2NH) ppm.

^13^C-NMR (101 MHz, DMSO-*d*_6_): δ = 30.61 (CO*C*H_2_), 37.70 (CH), 52.08 (NCH_2_), 111.08, 111.19, 118.52, 118.70, 119.29, 119.46, 121.19, 121.61, 122.02, 122.37, 125.54, 126.93, 128.70, 134.57, 135.00, 140.52, 142.82, 143.87 (C_ar_), 150.93, 154.95 (N=C), 172.42 (C=O) ppm.

IR (KBr): ν_max_ = 3197 (2NH), 1680 (C=O) cm^−1^.

MS (APCI+, 25 V) *m*/*z*, %: 394 (100) [M + H]^+^.

Calcd. for C_24_H_19_N_5_O, %: C 73.27; H 4.87; N 17.80. Found, %: C 73.20; H 4.82; N 17.76.

*3-(6-Methyl-1H-benzimidazol-2-yl)-1-[4-(6-methyl-1H-benzimidazol-2-yl)phenyl]pyrrolidin-5-one* (**15**)**:** ^1^H-NMR (400 MHz, DMSO-*d*_6_): δ = 2.39 (s, 3H, CH_3_), 2.42 (s, 3H, CH_3_), 2.99–3.12 (m, 2H, CO*C*H_2_), 3.98–4.05 (m, 1H, CH), 4.24–4.37 (m, 2H, NCH_2_), 6.97–7.05 (m, 2H, H_ar_), 7.29–7.48 (m, 4H, H_ar_), 7.88 (d, *J* = 8.9 Hz, 2H, H_ar_), 8.17 (d, *J* = 8.8 Hz, 2H, H_ar_), 12.51 (br. s, 2H, 2NH) ppm.

^13^C-NMR (101 MHz, DMSO-*d*_6_): δ = 21.28 (CH_3_), 21.36 (CH_3_), 30.63 (CO*C*H_2_), 37.72 (CH), 52.12 (NCH_2_), 113.54, 117.43, 117.54, 119.26, 119.47, 119.92, 122.58, 122.97, 123.45, 125.73, 126.79, 127.62, 128.70, 130.70, 131.16, 140.37 (C_ar_), 150.41, 150.64, 154.56 (N=C), 172.42 (C=O) ppm.

IR (KBr): ν_max_ = 3411, 3243 (2NH), 1684 (C=O) cm^−1^.

MS (APCI+, 25 V) *m*/*z*, %: 422 (100) [M + H]^+^.

Calcd. for C_26_H_23_N_5_O, %: C 74.09; H 5.50; N 16.62. Found, %: C 74.17; H 5.44; N 16.54.

*3-(1H-benzimidazol-2-yl)-4-[4-(1H-benzimidazol-2-yl)anilino]butanoic acid* (**16**)**:** A mixture of benzimidazole **14** (1.97 g, 5 mmol) and aqueous 20% sodium hydroxide (30 mL) solution was heated at reflux for 2 h, and then was cooled down, diluted with water (50 mL) and filtered off. The filtrate was acidified with 10% acetic acid to pH 6. The formed solid was filtered off, washed with water, and purified by dissolving it in 5% sodium hydroxide solution, filtering and acidifying the filtrate with 10% acetic acid to pH 6 (procedure was performed twice) to give the title compound **16** (light brown solid, yield 1.03 g, 50%, m. p. 204–205 °C).

^1^H-NMR (400 MHz, DMSO-*d*_6_): δ = 2.76–2.96 (m, 2H, COCH_2_), 3.29–3.75 (m, 3H, NHC*H*_2_+CH), 6.40 (s, 1H, N*H*CH_2_), 6.78 (d, *J* = 8.5 Hz, 2H, H_ar_), 7.02–7.24 (m, 4H, H_ar_), 7.38–7.62 (m, 4H, H_ar_), 7.92 (d, *J* = 8.6 Hz, 2H, H_ar_), 12.51 (br. s, 3H, OH + 2NH) ppm.

^13^C-NMR (101 MHz, DMSO-*d*_6_): δ = 35.64 (CO*C*H_2_), 36.18 (CH), 46.49 (NHCH_2_), 111.90, 117.52, 121.24, 121.29, 127.76, 149.92 (C_ar_), 152.47, 155.98 (2N=C), 173.44 (C=O) ppm. 

MS (APCI+, 25 V) *m*/*z*, %: 412 (100) [M + H]^+^.

Calcd. for C_24_H_21_N_5_O_2_, %: C 70.06; H 5.14; N 17.02. Found, %: C 70.17; H 5.07; N 17.08.

*4-((4-(1H-benzimidazol-2-yl)phenyl)amino)-3-(1H-benzimidazol-2-yl)butanehydrazide* (**17**). Method A: A mixture of butanoic acid **16** (2.06 g, 5 mmol), hydrazine monohydrate (2.10 g, 42 mmol), and 2-propanol (20 mL) was heated at reflux for 20 h and then cooled to room temperature. The obtained solid was filtered off, washed with water, and recrystallized from water to give the title compound **17** (light brown solid, yield 1.08 g, 51%, m. p. 166–167 °C).

Method B: A mixture of pyrrolidinone **14** (1.97 g, 5 mmol) and hydrazine monohydrate (20 g, 400 mmol) was heated at reflux for 6 h and then was cooled to room temperature, diluted with 2-propanol (30 mL), and filtered off. The filtrate was evaporated under reduced pressure; the residue was poured with water and stirred for 10 min. The obtained solid was filtered off, washed with plenty of water, and recrystallized from water to give the title compound **14** (light brown solid, yield 1.34 g, 63%, m. p. 166–167 °C).

^1^H-NMR (400 MHz, DMSO-*d*_6_): δ = 2.56–2.75 (m, 2H, COCH_2_), 3.40–3.59 (m, 2H, NHC*H*_2_), 3.72–3.78 (m, 1H, CH), 4.43 (s, 2H, NH_2_), 6.34 (t, *J* = 5.9 Hz, 1H, N*H*CH_2_), 6.76 (d, *J* = 8.6 Hz, 2H, H_ar_), 7.09–7.15 (m, 4H, H_ar_), 7.42–7.58 (m, 4H, H_ar_), 7.91 (d, *J* = 8.4 Hz, 2H, H_ar_), 9.12 (s, 1H, CONH), 12.31 (s, 1H, NH), 12.47 (s, 1H, NH) ppm.

^13^C-NMR (101 MHz, DMSO-*d*_6_): δ = 35.54, (CH), 35.72 (CO*C*H_2_), 46.46 (NHCH_2_), 110.88, 111.87, 117.46, 118.18, 121.26, 127.71, 134.89, 143.21, 149.95 (C_ar_), 152.47, 156.12 (N=C), 169.90 (C=O) ppm.

IR (KBr): ν_max_ = 3410 (NHNH_2_), 1611 (C=O) cm^−1^.

MS (APCI+, 25 V) *m*/*z*, %: 426 (100) [M + H]^+^.

Calcd. for C_24_H_23_N_7_O, %: C 67.75; H 5.45; N 23.04. Found, %: C 67.84; H 5.36; N 23.12.

*4-((4-(1H-benzimidazol-2-yl)phenyl)amino)-3-(1H-benzimidazol-2-yl)-N-(2,5-dimethyl-1H-pyrrol-2-yl)butanamide* (**18**)**:** To a mixture of hydrazide **17** (2.13 g, 5 mmol) and hexane-2,5-dione (3.42 g, 30 mmol) in 2-propanol (50 mL), conc. hydrochloric acid (2.5 mL) was added dropwise; the mixture was heated at reflux for 4 h, then cooled to room temperature, and the solvent was evaporated under reduced pressure. The residue was poured with water (30 mL) and stirred for 10 min. The obtained solid was filtered off, washed with water, and recrystallized from methanol to give the title compound **18** (white solid, yield 1.79 g, 71%, m. p. 227–228 °C).

^1^H-NMR (400 MHz, DMSO-*d*_6_): δ = 1.62 (s, 3H, CH_3_), 1.95 (s, 3H, CH_3_), 2.93–3.09 (m, 2H, COCH_2_), 3.54–3.84 (m, 3H, CH + NHC*H*_2_), 5.52 (d, *J* = 3.1 Hz, 1H, C=CH), 5.57 (d, *J* = 3.1 Hz, 1H, C=CH), 6.90 (d, *J* = 8.7 Hz, 2H, H_ar_), 7.02 (s, 1H, N*H*CH_2_), 7.19–7.24 (m, 2H, H_ar_), 7.34–7.39 (m, 2H, H_ar_), 7.53–7.59 (m, 2H, H_ar_), 7.64–7.68 (m, 2H, H_ar_), 8.06 (d, *J* = 8.7 Hz, 2H, H_ar_), 10.86 (s, 1H, CONH), 13.68 (br. s, 2H, 2NH) ppm.

^13^C-NMR (101 MHz, DMSO-*d*_6_): δ = 10.50 (CH_3_), 11.00 (CH_3_), 34.97 (CO*C*H_2_), 35.55 (CH), 46.03 (NHCH_2_), 102.76, 102.81 (2C=CH), 112.12, 112.13, 113.52, 113.54, 122.08, 122.12, 123.87, 126.56, 126.79, 129.08, 134.17, 134.22, 139.09, 150.54 (C_ar_), 151.79, 154.99 (2N=C), 169.97 (C=O) ppm.

IR (KBr): ν_max_ = 3050 (NH), 1608 (C=O) cm^−1^.

Calcd. for C_30_H_29_N_7_O, %: C 71.55; H 5.80; N 19.47. Found, %: C 71.78; H 5.72; N 19.33.

*Ethyl 2-(2-(4-(4-(1-(2-ethoxy-2-oxoethyl)-1H-benzimidazol-2-yl)-2-oxopyrrolidin-1-yl)-phenyl)-1H-benzimidazol-1-yl)acetate* (**19**)**:** A mixture of benzimidazole **14** (3 g, 7.6 mmol), potassium carbonate (2.1 g, 15.2 mmol), TBAI (0.02 g), and acetone (50 mL) was boiled, and ethyl chloroacetate (9.9 mL, 91.4 mmol) was slowly added dropwise. The mixture was refluxed for 20 h and filtered while hot; the filtrate was cooled to room temperature and diluted with water (100 mL). The formed solid was filtered off, washed with plenty of water, and recrystallized from methanol to give the title compound **19** (light brown solid, yield 3.78 g, 88%, m. p. 106–107 °C).

^1^H-NMR (400 MHz, DMSO-*d*_6_): δ = 1.15 (t, *J* = 7.1 Hz, 3H, CH_2_C*H*_3_), 1.25 (t, *J* = 7.1 Hz, 3H, CH_2_C*H*_3_), 2.94–3.11 (m, 2H, COCH_2_), 4.10–4.38 (m, 7H, 2C*H*_2_CH_3_ + CH + NCH_2_), 5.23 (s, 2H, NCH_2_CO), 5.32 (s, 2H, NCH_2_CO), 7.20–7.30 (m, 4H, H_ar_), 7.51–7.72 (m, 4H, H_ar_), 7.75 (d, *J* = 8.7 Hz, 2H, H_ar_), 7.90 (d, *J* = 8.8 Hz, 2H, H_ar_).

^13^C-NMR (101 MHz, DMSO-*d*_6_): δ = 13.94 (CH_3_), 14.04 (CH_3_), 28.41 (CH), 37.76 (CO*C*H_2_), 44.41 (N*C*H_2_CO), 46.06 (N*C*H_2_CO), 51.90 (NCH_2_), 61.40 (*C*H_2_CH_3_), 61.49 (*C*H_2_CH_3_), 110.12, 110.59, 118.85, 119.06, 119.15, 121.92, 122.37, 125.11, 129.42, 135.76, 136.33, 140.46, 141.75, 142.40 (C_ar_), 152.87, 155.42 (2N=C), 168.24, 168.39 (2C=O), 172.33 (NC=O) ppm.

IR (KBr): ν_max_ = 1732, 1708, 1611 (3C=O) cm^−1^.

MS (APCI+, 25 V) *m*/*z*, %: [M + H]^+^ = 566 (100).

Calcd. for C_32_H_31_N_5_O_5_, %: C 67.95; H 5.52; N 12.38. Found, %: C 67.86; H 5.54; N 12.28.

*2-(2-(4-(4-(1-(2-Hydrazinyl-2-oxoethyl)-1H-benzimidazol-2-yl)-2-oxopyrrolidin-1-yl)phenyl)-1H-benzimidazol-1-yl)acetohydrazide* (**20**)**:** A mixture of diester **19** (1.98 g, 3.5 mmol), hydrazine monohydrate (1.42 g, 28.3 mmol) and 1,4-dioxane (20 mL) was refluxed for 18 h and cooled to room temperature; the formed solid was filtered off, washed with water and 2-propanol, and recrystallized from methanol to give the title compound **20** (yellowish solid, yield 1.60 g, 85%, m. p. 206–207 °C).

^1^H-NMR (400 MHz, DMSO-*d*_6_): δ = 2.99–3.15 (m, 2H, COCH_2_), 4.00–4.74 (m, 7H, CH + NCH_2_ + 2NH_2_), 4.87, 4.95, 5.24, 5.32 (4s, 2H, NCH_2_CO), 7.00–7.57 (m, 6H, H_ar_), 7.58–8.00 (m, 6H, H_ar_), 8.83, 8.88, 9.60, 9.65 (4s, 2H, 2NH).

^13^C-NMR (101 MHz, DMSO-*d*_6_): δ = 28.55 (CH), 37.81 (CO*C*H_2_), 44.47 (N*C*H_2_CO), 45.78 (N*C*H_2_CO), 52.09 (NCH_2_), 110.02, 110.54, 118.82, 118.99, 119.03, 121.75, 122.17, 125.28, 129.78, 135.79, 136.38, 140.46, 141.79, 142.49 (C_ar_), 153.20, 155.77 (2N=C), 166.01, 166.34 (2C=O), 172.54 (NC=O) ppm.

IR (KBr): ν_max_ = 1729, 1674 (3C=O) cm^−1^.

MS (APCI+, 25 V) *m*/*z*, %: [M + H]^+^ = 566 (100).

Calcd. for C_38_H_30_N_9_O_3_, %: C 68.56; H 5.30; N 18.94. Found, %: C 68.62; H 5.25; N 18.87.

*4-(1-(2-(3,5-Dimethyl-1H-pyrazol-1-yl)-2-oxoethyl)-1H-benzimidazol-2-yl)-1-(4-(1-(3,5-dimethyl-1H-pyrazol-1-yl)-2-oxoethyl)-1H-benzimidazol-2-yl)phenyl)pyrrolidin-2-one* (**21**)**:** A mixture of hydrazide **20** (2.15 g, 4 mmol), 2,4-pentanedione (4.4 g, 44 mmol), 2-propanol and conc. hydrochloric acid (0.25 mL) was refluxed for 5 h and then cooled down and diluted with water. The formed precipitate was filtered off, washed with water, and recrystallized from methanol to give the title compound **21** (light yellow solid, yield 2.17 g, 82%, m. p. 178–179 °C).

^1^H-NMR (400 MHz, DMSO-*d*_6_): δ = 1.14 (s, 3H, CH_3_), 1.16 (s, 3H, CH_3_), 1.24 (s, 3H, CH_3_), 1.25 (s, 3H, CH_3_), 2.91–3.11 (m, 2H, COCH_2_), 4.11–4.19 (m, 1H, CH), 4.25–4.37 (m, 2H, NCH_2_), 4.89–4.96 (m, 1H, C=CH), 4.97–5.06 (m, 1H, C=CH), 5.20, 5.30 (4s, 4H, 2NCH_2_CO), 7.20–7.30 (m, 4H, H_ar_), 7.47–7.72 (m, 4H, H_ar_), 7.76 (d, *J* = 8.6 Hz, 2H, H_ar_), 7.90 (d, *J* = 8.8 Hz, 2H, H_ar_).

^13^C-NMR (101 MHz, DMSO-*d*_6_): δ = 21.37 (CH_3_), 21.53 (CH_3_), 28.43, 37.72, 44.60, 46.25, 51.90 (CH, CO*C*H_2_, N*C*H_2_CO, NCH_2_), 69.19, 69.32 (C-CH-C), 110.04, 110.51, 118.86, 119.06, 119.14, 121.90, 122.25, 122.37, 125.19, 129.37, 135.79, 136.36, 140.45, 141.74, 142.39 (C_ar_), 152.85, 155.37 (2N=C), 167.75, 167.89 (2C=O), 172.31 (NC=O) ppm.

IR (KBr): ν_max_ = 3279, 3203 (2NHNH_2_), 1689, 1610 (3C=O) cm^−1^.

Calcd. for C_28_H_27_N_9_O3_5_, %: C 62.56; H 5.06; N 23.45. Found, %: C 62.31; H 4.99; N 23.27.

*4-(1-((5-Thioxo-4,5-dihydro-1,3,4-oxadiazol-2-yl)methyl)-1H-benzo[d]imidazol-2-yl)-1-(4-(1-((5-thioxo-4,5-dihydro-1,3,4-oxadiazol-2-yl)methyl)-1H-benzo[d]imidazol-2-yl)phenyl)pyrrolidin-2-one* (**22**)**:** To a cooled solution of potassium hydroxide (1 g, 18 mmol) in methanol (80 mL), carbon disulfide (0.7 mL, 11 mmol) was added dropwise and the obtained mixture was stirred at room temperature for 15 min. Then hydrazide **20** (2.15 g, 4 mmol) was added slowly, and the mixture was heated at reflux for 12 h. After completion of the reaction (TLC), the volatile fraction was evaporated under reduced pressure; the residue was dissolved in water (100 mL) and boiled for 3 min with activated carbon. The mixture was filtered off, and the filtrate was acidified with diluted hydrochloric acid to pH 1. The formed precipitate was filtered off, washed with water, and recrystallized from methanol to give the title compound **22** (white/yellow solid, yield 2.02 g, 81%, m. p. 262–263 °C).

^1^H-NMR (400 MHz, DMSO-*d*_6_): δ = 2.90–3.17 (m, 2H, COCH_2_), 4.06–4.53 (m, 2H, NCH_2_ + CH), 5.72, 5.81, 5.90, 6.01 (4s, 4H, 2NCH_2_CO), 7.19–8.04 (m, 12H, H_ar_), 12.41 (br. s, 2H, 2NH) ppm.

^13^C-NMR (101 MHz, DMSO-*d*_6_): δ = 28.45, 37.82, 38.08, 44.35, 51.94 (CH, CO*C*H_2_, N*C*H_2_CO, NCH_2_), 110.40, 110.87, 119.03, 119.13, 119.24, 122.29, 122.73, 124.48, 129.78, 135.35, 135.76, 140.67, 141.76, 142.42, 155.20, 164.35, 165.63, 165.93 (C_ar_), 172.36, 172.42 (C=O, 2C=S) ppm.

IR (KBr): ν_max_ = 3425 (2NH), 1609 (C=O), 1327 (C=S) cm^−1^.

Calcd. for C_30_H_26_N_9_O_3_S_2_, %: C 57.96; H 3.73; N 20.28. Found, %: C 57.87; H 3.80; N 20.21.

*2-(2-(-2-(5-Oxo-1-(4-(1-(2-oxo-2-(2-(phenylcarbamothioyl)hydrazinyl)ethyl)-1H-benzimidazol-2-yl)phenyl)pyrrolidin-3-yl)-1H-benzimidazol-1-yl)acetyl)-N-phenylhydrazin-1-carbothioamide* (**23**)**:** To a boiled mixture hydrazide **20** (2.15 g, 4 mmol) and methanol (100 mL), phenyl isothiocyanate (2.03 g, 15 mmol) was added dropwise, and the obtained mixture was heated at reflux for 6 h and then cooled down; the obtained solid was filtered off, washed with water and cold methanol, and recrystallized from methanol to give the title compound **23** (light yellow solid, yield 2.91 g, 90%, m. p. 240 (decomp.) °C).

^1^H-NMR (400 MHz, DMSO-*d*_6_): δ = 3.01–3.17 (m, 2H, COCH_2_), 4.11–4.37 (m, 3H, CH + NCH_2_), 4.89, 4.97, 5.06, 5.15 (4s, 4H, 2NCH_2_CO), 7.08–7.95 (m, 22H, H_ar_), 9.48–10.04 (m, 4H, 4NH), 10.51, 10.58 (2s, 2H, 2NH) ppm.

IR (KBr): ν_max_ = 3206 (NH), 1694, 1608 (3C=O) cm^−1^.

Calcd. for C_42_H_37_N_11_O_3_S_2_, %: C 62.44; H 4.62; N 19.07. Found, %: C 62.57; H 4.69; N 19.16.

General procedure for the preparation of hydrazones **24a**–**e**: To a solution of hydrazide **20** (2.15 g, 4 mmol) in DMF (100 mL), the corresponding aromatic aldehyde (30 mmol) was added, and the mixture was heated at reflux for 5 (**a**,**c**–**e**) or 8 (**b**) h. After completion of the reaction, the mixture was cooled to room temperature and diluted with water (**a**,**c**–**e**). Upon cooling the reaction mixture **b**, a solid formed in the DMF. The obtained crystalline substance was filtered off, washed with water, 2-propanol, and diethyl ether, and recrystallized from the mixture of 1,4-dioxane and 2-propanol (1:2) to give the corresponding title compound **24**.

*N’-benzylidene-2-(2-(4-(4-(1-(2-(2-(benzylidene)hydrazinyl)-2-oxoethyl)-1H-benzo[d]imidazol-2-yl)-2-oxopyrrolidin-1-yl)phenyl)-1H-benzo[d]imidazol-1-yl)acetohydrazide* (**24a**)**:** Light brown solid, yield 2.34 g, 82%, m. p. 254–255 °C. ^1^H-NMR (400 MHz, DMSO-d_6_): δ = Z/E 2.95–3.13 (m, 2H, COCH_2_), 4.09–4.41 (m, 3H, CH + NCH_2_), 5.08, 5.14, 5.54, 5.61 (4s, 4H, 2NCH_2_CO), 7.20–8.00 (m, 22H, H_ar_), 8.06, 8.11 (2s, 0.75(2H), 2N=CH), 8.26, 8.30 (2s, 0.25(2H), 2N=CH), 11.82, 11.89, 12.00, 12.04 (4s, 2H, 2NH) ppm.

IR (KBr): ν_max_ = 3200 (2NH), 1693, 1610 (3C=O) cm^−1^.

Calcd. for C_42_H_35_N_9_O_3_, %: C 70.67; H 4.94; N 17.66. Found, %: C 70.62; H 4.98; N 17.58.

*N’-4-nitrobenzylidene-2-(2-(4-(4-(1-(2-(2-(4-nitrobenzylidene)hydrazinyl)-2-oxoethyl)-1H-benzo[d]imidazol-2-yl)-2-oxopyrrolidin-1-yl)phenyl)-1H-benzo[d]imidazol-1-yl)acetohydrazide* (**24b**)**:** Light yellow solid, yield 2.48 g, 77%, m. p. 213–214 °C. ^1^H-NMR (400 MHz, DMSO-d_6_): δ = Z/E 2.90–3.11 (m, 2H, COCH_2_), 4.02–4.43 (m, 3H, CH + NCH_2_), 5.12, 5.18, 5.60, 5.66 (4s, 4H, 2NCH_2_CO), 7.09–7.43 (m, 4H, H_ar_), 7.46–8.46 (m, 18H, H_ar_ + 2N=CH), 12.08, 12.14,12.24, 12.26 (4s, 2H, 2NH) ppm.

IR (KBr): ν_max_ = 3438 (2NH), 1694, 1610 (3C=O) cm^−1^.

Calcd. for C_42_H_33_N_11_O_5_, %: C 62.76; H 4.14; N 19.17. Found, %: C 62.81; H 4.20; N 19.14.

*N’-(4-fluorobenzylidene)-2-(2-(4-(4-(1-(2-(2-(4-fluorobenzylidene)hydrazinyl)-2-oxoethyl)-1H-benzo[d]imidazol-2-yl)-2-oxopyrrolidin-1-yl)phenyl)-1H-benzo[d]imidazol-1-yl)acetohydrazide* (**24c**)**:** White solid, yield 2.40 g, 80%, m. p. 268–269 °C. ^1^H-NMR (400 MHz, DMSO-d_6_): δ = Z/E 2.93–3.13 (m, 2H, COCH_2_), 4.08–4.20 (m, 1H, CH), 4.21–4.39 (m, 2H, NCH_2_), 5.08, 5.14,5.54, 5.61 (4s, 4H, 2NCH_2_CO), 7.05–7.46 (m, 8H, H_ar_), 7.47–7.96 (m, 12H, H_ar_), 8.05, 8.10 (2s, 0.75(2H), 2N=CH), 8.26, 8.30 (2s, 0.25(2H), 2N=CH), 11.82, 11.88, 12.00, 12.03 (4s, 2H, 2NH) ppm.

IR (KBr): ν_max_ = 3206 (2NH), 1694, 1608 (3C=O) cm^−1^.

Calcd. for C_42_H_33_F_2_N_9_O_3_, %: C 62.44; H 4.62; N 19.07. Found, %: C 62.47; H 4.69; N 19.16.

*N’-3-chlorobenzylidene)-2-(2-(4-(4-(1-(2-(2-(3-chlorobenzylidene)hydrazinyl)-2-oxoethyl)-1H-benzo[d]imidazol-2-yl)-2-oxopyrrolidin-1-yl)phenyl)-1H-benzo[d]imidazol-1-yl)acetohydrazide* (**24d**)**:** Light brown solid, yield 2.47 g, 79%, m. p. 256–257 °C. ^1^H-NMR (400 MHz, DMSO-d_6_): δ = Z/E 2.95–3.09 (m, 2H, COCH_2_), 3.99–4.52 (m, 3H, CH + NCH_2_), 5.09, 5.15, 5.56, 5.63 (4s, 4H, 2NCH_2_CO), 7.08–7.96 (m, 20H, H_ar_), 8.03, 8.08 (2s, 0.8(2H), 2N=CH), 8.23, 8.27 (2s, 0.2(2H), 2N=CH), 11.89, 11.95, 12.11, 12.14 (4s, 2H, 2NH) ppm.

IR (KBr): ν_max_ = 3393, 3207 (2NH), 1685, 1611 (3C=O) cm^−1^.

Calcd. for C_42_H_33_Cl_2_N_9_O_3_, %C 64.45; H 4.25; N 16.11. Found, %: C 64.36; H 4.20; N 16.20.

*N’-(2,3-dimethoxybenzylidene)-2-(2-(4-(4-(1-(2-(2-(2,3-dimethoxybenzylidene)hydrazinyl)-2-oxoethyl)-1H-benzo[d]imidazol-2-yl)-2-oxopyrrolidin-1-yl)phenyl)-1H-benzo[d]imidazol-1-yl)acetohydrazide* (**24e**)**:** Light yellow solid, yield 3.14 g, 94%, m. p. 194–195 °C. ^1^H-NMR (400 MHz, DMSO-d_6_): δ = Z/E 2.94–3.11 (m, 2H, COCH_2_), 3.70, 3.76, 3.79, 3.81 (4s, 12H, 4CH_3_O), 4.10–4.44 (m, 1H, CH+NCH_2_), 5.05, 5.11, 5.54, 5.61 (4s, 4H, 2NCH_2_CO), 6.96–7.91 (m, 18H, H_ar_), 8.36, 8.40 (2s, 0.75(2H), 2N=CH), 8.57, 8.60 (2s, 0.25(2H), 2N=CH), 11.77, 11.83, 12.00, 12.01 (4s, 2H, 2NH) ppm. IR (KBr): ν_max_ = 3200, 3054 (2NH), 1682, 1610 (3C=O) cm^−1^. Calcd. for C_46_H_43_N_9_O_7_, %: C, 66.26; H, 5.20; N, 15.12. Found, %: C 66.19; H 5.26; N 15.21.

### 3.2. Determination of Antimicrobial Activity 

#### 3.2.1. Preparation of Bacterial Inoculum

A panel of clinically important reference bacterial pathogens were obtained from the American Type Culture Collection (ATCC) and the National Type Culture Collection (NTCT) (Supplementary Table S1). Each test organism was subcultured on Tryptic Soy Agar (TSA) at 37 °C, for 24 h. After incubation, the representative colonies were suspended in 5 mL of Tryptic Soy Broth (TBS) and further incubated at 37 °C for 24 h to initiate the liquid culture. The bacterial cultures were normalized using a spectrophotometer (OD_600 nm_), and the final inoculum (1 × 10^7^ CFU/mL) was achieved by diluting the culture with fresh TSB.

#### 3.2.2. Preparation of the Test Compounds

The test compounds **3**–**24** were dissolved in hybridoma grade DMSO to achieve a 50 mg/mL stock solution. The stock solution was further diluted in TSB supplemented with 1% of DMSO to produce the series of dilutions (0, 15.62, 31.25, 62.5, 125, 250, 500, and 1000 µg/mL). Ampicillin was dissolved in sterile deionized water and the series of dilutions (0, 15.62, 31.25, 62.5, 125, 250, 500, and 1000 µg/mL) were prepared as described above.

#### 3.2.3. Evaluation of Minimal Inhibitory Concentration

The minimal inhibitory concentration (MIC) of the compounds **3**–**24** and ampicillin were determined by the broth dilution method as described by Balouiri et al. [75] with brief modifications. The tubes containing diluted compounds in TSB were inoculated with normalized bacterial inoculum (100 µL) to achieve a final bacterial concentration of 1 × 10^6^ CFU/mL. The inoculated tubes were incubated at 37 °C for 24 h. After incubation, the turbidity was evaluated visually and MIC was estimated. The MIC was defined as the lowest concentration of the test compound that inhibits the visual growth of the test organism.

#### 3.2.4. Determination of Minimal Bactericidal Concentration

The minimal bactericidal concentration (MBC) was determined as described by Parvekar et al. [76]. After a MIC evaluation of the novel compounds and ampicillin, aliquots of 100 µL were taken from tubes without growth and plated on TSA. The plates were incubated at 37 °C for 48 h. After incubation, the plates were evaluated, and the minimal bactericidal concentration (MBC) was estimated. The MBC was defined as the lowest concentration of the test compound that fully suppresses the growth of the test organism.

## 4. Conclusions

In this study, the chemical transformations of *p*-aminobenzoic acid were carried out, and a series of 1-phenyl-5-oxopyrrolidine derivatives with hydrazone, pyrazole, thiosemicarbazide, triazole, oxadiazole fragments were obtained. A convenient and efficient method for the synthesis of benzimidazoles by heating reagents in polyphosphoric acid was proposed.

The synthesized compounds were evaluated for their antibacterial activity against a panel of clinically relevant Gram-positive and Gram-negative pathogens. The antimicrobial activity evaluation revealed that the *γ*-amino acid derivative **16** bearing two benzimidazole fragments demonstrated the strongest broad-spectrum bactericidal activity on both Gram-positive and Gram-negative organisms. The antimicrobial activity of compound **16** was notably greater than that of ampicillin. Furthermore, benzimidazoles **14** and **15** showed promising, broad-spectrum antibacterial activity against tested pathogens, with notably good bactericidal activity against *L. monocytogenes*. Collectively, these results demonstrated that the 5-oxopyrrolidine **14** could be further explored as a potential pharmacophore in the development of novel antimicrobials targeting clinically significant bacterial pathogens. Further studies are needed to better understand the safety, tolerability, and in vivo activity of the most promising compounds.

## Figures and Tables

**Table 1:** Reaction conditions for the synthesis of benzimidazoles **14** and **15** and their corresponding product yields.

Entry	Reagent	Solvent/ Catalyst	Temperature, °C	Time, h	Yield, %
**1**	Benzene-1,2- diamine	-	170; 230	2; 0.5	51
**2**		15% HCl	Reflux	96	8
**3**		-	MW, 140 W	0.25	40
**4**		2-PrOH/NH_4_Cl	Reflux	25	12
**5**		PPA	120	6	97 (**14**); 91 (**15**)

**Table 2:** Minimal inhibitory concentrations (MIC) as well as minimal bactericidal concentrations (MBC) of 5-oxopyrrolidine derivatives **3**–**24** against various bacterial strains.

**Compound**	Gram-Positive Bacteria						Gram-Negative Bacteria					
	*S. aureus* ATCC 9144		*B. cereus* ATCC 11778		*L. monocytogenes* ATCC 7644		*S. enteritidis* ATCC 13076		*E. coli* ATCC 8739		*P. aeruginosa* NCTC 6750	
	MIC	MBC	MIC	MBC	MIC	MBC	MIC	MBC	MIC	MBC	MIC	MBC
	µg/mL											
**3**	250	250	31.25	31.25	125	125	250	250	250	250	250	250
**4**	125	125	125	250	250	250	250	250	250	250	250	250
**5**	250	250	31.25	31.25	125	125	125	125	125	125	125	125
**6**	250	250	125	125	250	250	125	125	125	125	125	125
**7a**	250	250	250	250	125	125	125	125	250	250	125	125
**7b**	250	250	250	250	250	250	125	125	250	250	125	125
**7c**	250	250	250	250	125	125	125	125	250	250	125	125
**8**	250	250	125	125	125	125	62.5	125	125	125	62.5	125
**9**	125	250	250	250	250	250	125	125	250	250	125	125
**10**	125	125	125	125	125	125	125	125	125	125	125	125
**11a**	>250	>250	>250	>250	>250	>250	>250	>250	>250	>250	>250	>250
**11b**	250	250	>250	>250	>250	>250	>250	>250	>250	>250	>250	>250
**12**	125	250	125	250	125	250	125	250	125	250	125	250
**13**	125	125	125	250	62.5	62.5	125	125	125	250	62.5	62.5
**14**	62.5	62.5	62.5	62.5	15.62	15.62	62.5	62.5	62.5	62.5	62.5	62.5
**15**	62.5	125	62.5	125	15.62	31.25	62.5	62.5	62.5	125	62.5	62.5
**16**	31.25	31.25	31.25	31.25	31.25	31.25	31.25	31.25	31.25	31.25	31.25	31.25
**17**	31.25	62.5	62.5	62.5	62.5	62.5	62.5	62.5	62.5	62.5	62.5	62.5
**19**	31.25	31.25	62.5	62.5	62.5	62.5	31.25	62.5	62.5	62.5	62.5	62.5
**20**	62.5	62.5	31.25	31.25	62.5	62.5	62.5	62.5	62.5	62.5	62.5	62.5
**21**	125	125	62.5	62.5	62.5	62.5	62.5	62.5	62.5	62.5	125	125
**22**	62.5	62.5	62.5	62.5	62.5	62.5	31.25	31.25	62.5	62.5	62.5	62.5
**24a**	125	125	125	125	125	125	125	125	125	125	62.5	125
**24b**	62.5	62.5	62.5	62.5	62.5	62.5	62.5	62.5	31.25	31.25	31.25	31.25
**24c**	125	125	125	125	125	125	62.5	62.5	125	125	125	62.5
**24d**	125	125	125	125	62.5	62.5	125	125	125	125	125	125
**24e**	62.5	125	62.5	62.5	62.5	62.5	62.5	62.5	62.5	125	62.5	62.5
Ampicillin 62.5												

## Data Availability

Not applicable.

## Supplementary Materials

The following are available online, Figure S1: ^1^H-NMR of compound **2**, Figure S2: ^13^C-NMR of compound **2**, Figure S3: ^1^H-NMR of compound **3**, Figure S4: ^13^C-NMR of compound **3**, Figure S5: ^1^H-NMR of compound **4**, Figure S6: ^13^C-NMR of compound **4**, Figure S7: ^1^H-NMR of compound **5**, Figure S8: ^13^C-NMR of compound **5**, Figure S9: ^1^H-NMR of compound **6**, Figure S10: ^13^C-NMR of compound **6**, Figure S11: ^1^H-NMR of compound **7a**, Figure S12: ^1^H-NMR of compound **7b**, Figure S13: ^1^H-NMR of compound **7c**, Figure S14: ^1^H-NMR of compound **8**, Figure S15: ^13^C-NMR of compound **8**, Figure S16: ^1^H-NMR of compound **9**, Figure S17: ^13^C-NMR of compound 9, Figure S18: ^1^H-NMR of compound **10**, Figure S19: ^13^C-NMR of compound **10**, Figure S20: ^1^H-NMR of compound **11a**, Figure S21: ^1^H-NMR of compound **11b**, Figure S22: ^1^H NMR of compound **12**, Figure S23: ^13^C-NMR of compound **12**, Figure S24: ^1^H-NMR of compound **13**, Figure S25: ^13^C-NMR of compound **13**, Figure S26: ^1^H-NMR of compound **14**, Figure S27: ^13^C-NMR of compound **14**, Figure S28: ^1^H-NMR of compound **15**, Figure S29: ^13^C-NMR of compound **15**, Figure S30: ^1^H-NMR of compound **16**, Figure S31: ^13^C-NMR of compound **16**, Figure S32: ^1^H-NMR of compound **17**, Figure S33: ^13^C-NMR of compound **17**, Figure S34: ^1^H-NMR of compound **18**, Figure S35: ^13^C-NMR of compound **18**, Figure S36: ^1^H-NMR of compound **19**, Figure S37: ^13^C-NMR of compound **19**, Figure S38: ^1^H-NMR of compound **20**, Figure S39: ^13^C-NMR of compound **20**, Figure S40: ^1^H-NMR of compound **21**, Figure S41: ^13^C-NMR of compound **21**, Figure S42: ^1^H-NMR of compound **22**, Figure S43: ^13^C-NMR of compound **22**, Figure S44: ^1^H-NMR of compound **23**, Figure S45: ^1^H-NMR of compound **24a**, Figure S46: ^1^H-NMR of compound **24b**, Figure S47: ^1^H-NMR of compound **24c**, Figure S48: ^1^H-NMR of compound **24d**, Figure S49: ^1^H-NMR of compound **24e**. Table S1: Bacteria strains used in the biological evaluation.

## Sample Availability

Samples of the compounds are not available from the authors.

## References

[1] Wright G.D. (2005). Bacterial resistance to antibiotics: Enzymatic degradation and modification. Adv. Drug Deliv. Rev..

[2] Horcajada J.P., Montero M., Oliver A., Sorlí L., Luque S., Gómez-Zorrilla S., Benito N., Grau S. (2019). Epidemiology and Treatment of Multidrug-Resistant and Extensively Drug-Resistant Pseudomonas aeruginosa Infections. Clin. Microbiol. Rev..

[3] Exner M., Bhattacharya S., Christiansen B., Christiansen B., Gebel J., Goroncy-Bermes P., Hartemann P., Heeg P., Ilschner C., Kramer A. (2017). Antibiotic resistance: What is so special about multidrug-resistant Gram-negative bacteria?. GMS Hyg. Infect. Control.

[4] European Food Safety Authority, European Centre for Disease Prevention and Control (2019). The European Union summary report on antimicrobial resistance in zoonotic and indicator bacteria from humans, animals and food in 2017. EFSA J..

[5] Lee C.R., Lee J.H., Park M., Park K.W., Bae I.K., Kim Y.B., Cha C.-J., Jeong B.C., Lee S.H. (2017). Biology of Acinetobacter baumannii: Pathogenesis, Antibiotic Resistance Mechanisms, and Prospective Treatment Options. Front. Cell. Infect. Microbiol..

[6] Fodor A., Abate B.A., Deák P., Fodor L., Gyenge E., Klein M.G., Koncz Z., Muvevi J., Ötvös L., Székely G. (2020). Multidrug Resistance (MDR) and Collateral Sensitivity in Bacteria, with Special Attention to Genetic and Evolutionary Aspects and to the Perspectives of Antimicrobial Peptides-A Review. Pathogens.

[7] Geisinger E., Isberg R.R. (2017). Interplay between Antibiotic Resistance and Virulence During Disease Promoted by Multidrug-Resistant Bacteria. J. Infect. Dis..

[8] Toutain P.L., Bousquet-Mélou A., Damborg P., Ferran A.A., Mevius D., Pelligand L., Veldman K.T., Lees P. (2017). En Route towards European Clinical Breakpoints for Veterinary Antimicrobial Susceptibility Testing: A Position Paper Explaining the VetCAST Approach. Front. Microbiol..

[9] Frosini S.M., Bond R., McCarthy A.J., Feudi C., Schwarz S., Lindsay J.A., Loeffler A. (2020). Genes on the Move: In Vitro Transduction of Antimicrobial Resistance Genes between Human and Canine Staphylococcal Pathogens. Microorganisms.

[10] Partridge S.R., Kwong S.M., Firth N., Jensen S.O. (2018). Mobile Genetic Elements Associated with Antimicrobial Resistance. Clin. Microbiol. Rev..

[11] Pagano M., Martins A.F., Barth A.L. (2016). Mobile genetic elements related to carbapenem resistance in Acinetobacter baumannii. Braz. J. Microbiol..

[12] Bansal G., Allen-McFarlane R., Eribo B. (2020). Antibiotic Susceptibility, Clonality, and Molecular Characterization of Carbapenem-Resistant Clinical Isolates of Acinetobacter baumannii from Washington DC. Int. J. Microbiol..

[13] Mulani M.S., Kamble E.E., Kumkar S.N., Tawre M.S., Pardesi K.R. (2019). Emerging Strategies to Combat ESKAPE Pathogens in the Era of Antimicrobial Resistance: A Review. Front. Microbiol..

[14] Pendleton J.N., Gorman S.P., Gilmore B.F. (2013). Clinical relevance of the ESKAPE pathogens. Expert. Rev. Anti Infect. Ther..

[15] De Oliveira D.M.P., Forde B.M., Kidd T.J., Harris P.N.A., Schembri M.A., Beatson S.A., Paterson D.L., Walker M.J. (2020). Antimicrobial Resistance in ESKAPE Pathogens. Clin. Microbiol. Rev..

[16] Kluczyk A., Popek T., Kiyota T., de Macedo P., Stefanowicz P., Lazar C., Yasuo Konishi Y. (2002). Drug evolution: P-aminobenzoic acid as a building block. Curr. Med. Chem..

[17] Pan X., Zheng Y., Chen R., Qiu S., Chen Z., Rao W., Chen S., You Y., Lü J., Xu L. (2019). Cocrystal of Sulfamethazine and *p*-Aminobenzoic Acid: Structural Establishment and Enhanced Antibacterial Properties. Cryst. Growth Des..

[18] Veeravarapu H., Tirumalasetty M., Kurati S.P., Wunnava U., Muthyala M.K.K. (2020). Design, synthesis, antimycobacterial activity and molecular docking studies of novel 3- (N-substituted glycinamido) benzoic acid analogues as antitubercular agents. Bioorg. Med. Chem. Lett..

[19] Vasilieva S. (2001). *Para*-aminobenzoic acid inhibits a set of SOS functions in *Escherichia coli* K12. Mutat. Res. Genet. Toxicol. Environ. Mutagen..

[20] Markitantova Y.V., Akberova S.I., Ryabtseva A.A., Stroeva O.G. (2018). The Effect of *para*-Aminobenzoic Acid on Apoptosis Processes in the Adult Rat Conjunctiva and Corneal Epithelium in vivo after Hypobaric Hypoxia. Biol. Bull. Russ. Acad. Sci..

[21] Sowinska M., Morawiak M., Bochyńska-Czyż M., Lipkowski A.W., Ziemińska E., Zabłocka B., Urbanczyk-Lipkowska Z. (2019). Molecular Antioxidant Properties and In Vitro Cell Toxicity of the *p*-Aminobenzoic Acid (PABA) Functionalized Peptide Dendrimers. Biomolecules.

[22] Akberova S.I. (2002). New biological properties of p-aminobenzoic acid. Biolog. Bull. Russ. Acad. Sci..

[23] Roden D. (2000). Antiarrhythmic drugs: From mechanisms to clinical practice. Heart.

[24] Pierrel F., Hamelin O., Douki T., Kieffer-Jaquinod S., Muhlenhoff U., Ozeir M., Lill R.M., Fontecave M. (2010). Involvement of mitochondrial ferredoxin and para-aminobenzoic acid in yeast coenzyme Q biosynthesis. Chem. Biol..

[25] Marbois B., Xie L.X., Choi S., Hirano K., Hyman K., Clarke C.F. (2010). para-Aminobenzoic acid is a precursor in coenzyme Q6 biosynthesis in Saccharomyces cerevisiae. J. Biol. Chem..

[26] Lu Z., Kong X., Lu Z., Xiao M., Chen M., Zhu L., Shen Y., Hu X., Song S. (2014). *Para*-Aminobenzoic Acid (PABA) Synthase Enhances Thermotolerance of Mushroom *Agaricus bisporus*. PLoS ONE.

[27] Song G.C., Choi H.K., Ryu C.-M. (2013). The folate precursor *para*-aminobenzoic acid elicits induced resistance against *Cucumber mosaic virus* and *Xanthomonas axonopodis*. Ann. Bot..

[28] Martinez F., Tolentino L.E., Campos M.E. (2013). Design of Compounds Derivatives from P-Amino Benzoic Acid as Inhibitor Cyclophilin a Theoretical Study. Free Radic. Biol. Med..

[29] Okey N.C., Obasi N.L., Ejikeme P.M., Ndinteh D.T., Ramasami P., Sherif E.-S.M., Akpan E.D., Ebenso E.E. (2020). Evaluation of some amino benzoic acid and 4-aminoantipyrine derived Schiff bases as corrosion inhibitors for mild steel in acidic medium: Synthesis, experimental and computational studies. J. Mol. Liq..

[30] Naushad M., Alqadami A.A., Al-Kahtani A.A., Ahamad T., Awual M.R., Tatarchuk T. (2019). Adsorption of textile dye using para-aminobenzoic acid modified activated carbon: Kinetic and equilibrium studies. J. Mol. Liq..

[31] Liu J., Zhang H.-R., Lin X.-R., Yan S.-J., Lin J. (2014). Catalyst-free cascade reaction of heterocyclic ketene aminals with *N*-substituted maleimide to synthesise bicyclic pyrrolidinone derivatives. RSC Adv..

[32] Tumosienė I., Kantminienė K., Jonuškienė I., Peleckis A., Belyakov S., Mickevičius V. (2019). Synthesis of 1-(5-Chloro-2-hydroxyphenyl)-5-oxopyrrolidine-3-carboxylic Acid Derivatives and their antioxidant activity. Molecules.

[33] Yoshie O., Matsushima K. (2015). CCR4 and its ligands: From bench to bedside. Int. Immunol..

[34] He X., Alian A., Stroud R., Ortiz de Montellano P.R. (2006). Pyrrolidine carboxamides as a novel class of inhibitors of enoyl acyl carrier protein reductase from Mycobacterium tuberculosis. J. Med. Chem..

[35] Gein V.L., Armisheva M.N., Rassudikhina N.A., Vakhrin M.I., Voronina E.V. (2011). Synthesis and antimicrobial activity of 1-(4-hydroxyphenyl)-4-acyl-5-aryl-3-hydroxy-3-pyrrolin-2-ones. Pharm. Chem. J..

[36] Wang W., Zhang L., Wang S., Shi S., Jiang Y., Li N., Tu P. (2014). 8-C *N*-ethyl-2-pyrrolidinone substituted flavan-3-ols as the marker compounds of Chinese dark teas formed in the post-fermentation process provide significant antioxidative activity. Food Chem..

[37] Geng Y., Wang X., Yang L., Sun H., Wang Y., Zhao Y., She R., Wang M.-X., Wang D.-X., Tang J. (2015). Antitumor Activity of a 5-Hydroxy-1H-Pyrrol-2-(5H)-One-Based Synthetic Small Molecule In Vitro and In Vivo. PLoS ONE.

[38] Moutevelis-Minakakis P., Papavassilopoulou E., Michas G., Georgikopoulou K., Ragoussi M.-E., Neophytou N., Zoumpoulakis P., Mavromoustakos T., Hadjipavlou-Litina D. (2011). Synthesis, in silico docking experiments of new 2-pyrrolidinone derivatives and study of their anti-inflammatory activity. Bioorg. Med. Chem..

[39] Vaškevičienė I., Paketurytė V., Pajanok N., Žukauskas Š., Sapijanskaitė B., Kantminienė K., Mickevičius V., Zubrienė A., Matulis D. (2019). Pyrrolidinone-bearing methylated and halogenated benzenesulfonamides as inhibitors of carbonic anhydrases. Bioorg. Med. Chem..

[40] Balandis B., Ivanauskaitė G., Smirnovienė J., Kantminienė K., Matulis D., Mickevičius V., Zubrienė A. (2020). Synthesis and structure-affinity relationship of chlorinated pyrrolidinone-bearing benzenesulfonamides as human carbonic anhydrase inhibitors. Bioorg. Chem..

[41] Gaba M., Singh S., Mohan C. (2014). Benzimidazole: An emerging scaffold for analgesic and anti-inflammatory agents. Eur. J. Med. Chem..

[42] Shin J.M., Sache G., Cho Y.M., Garst M. (2009). 1-Arylsulfonyl-2-(Pyridylmethylsulfinyl) Benzimidazoles as New Proton Pump Inhibitor Prodrugs. Molecules.

[43] Shinde V.S., Lawande P.P., Sontakke V.A., Khan A. (2020). Synthesis of benzimidazole nucleosides and their anticancer activity, *Carbohydr*. Res..

[44] Beltran-Hortelano I., Alcolea V., Font M., Pérez-Silanes S. (2020). The role of imidazole and benzimidazole heterocycles in Chagas disease: A review. Eur. J. Med. Chem..

[45] Li Y., Zhou X., Wu H., Yu Z., Li H., Yang S. (2020). Nanospheric heterogeneous acid-enabled direct upgrading of biomass feedstocks to novel benzimidazoles with potent antibacterial activities. Ind. Crops Prod..

[46] Tumosienė I., Peleckis A., Jonuškienė I., Vaickelionienė R., Kantminienė K., Šiugždaitė J., Beresnevičius Z.J., Mickevičius V. (2018). Synthesis of novel 1,2- and 2-substituted benzimidazoles with high antibacterial and antioxidant activity. Monatsh. Chem..

[47] Strelciunaite V., Anusevicius K., Tumosiene I., Siugzdaite J., Jonuskiene I., Ramanauskaite I., Mickevicius V. (2016). Synthesis of novel benzimidazoles 2-functionalized with pyrrolidinone and γ-amino acid with a high antibacterial activity. Heterocycles.

[48] Bhrigu B., Siddiqui N., Pathak D., Alam S.M., Ali R., Azad B. (2012). Anticonvulsant evaluation of some newer benzimidazole derivatives: Design and synthesis. Acta Pol. Pharm..

[49] Karadayi F.Z., Yaman M., Kisla M.M., Keskus A.G., Konu O., Ates-Alagoz Z. (2020). Design, synthesis and anticancer/antiestrogenic activities of novel indole-benzimidazoles. Bioorg. Chem..

[50] Singla R., Gupta K.B., Upadhyay S., Dhiman M. (2018). Design, synthesis and biological evaluation of novel indole-benzimidazole hybrids targeting estrogen receptor alpha (ER-α). Eur. J. Med. Chem..

[51] Wu Z., Xia M.-B., Bertsetseg D., Wang Y.-H., Bao X.-L., Zhu W.-B., Tao X., Chen P.-R., Tang H.-S., Yan Y.-J. (2020). Design, synthesis and biological evaluation of novel fluoro-substituted benzimidazole derivatives with anti-hypertension activities. Bioorg. Chem..

[52] Sallam M.A.E., Salem D.M.S.A., Labib G.M.H., Youssef T.N.M.A., Matsuo K. (2020). Studies on saccharide benzimidazoles: 2-(β-d-gulofuranosyl)benzimidazole and 2-(β-d-glucofuranosyl)benzimidazole C-nucleoside analogs; synthesis, anomeric configuration and antifouling potency. Carbohydr. Res..

[53] Chahkandi M., Bhatti M.H., Yunus U., Nadeem M., Rehman N., Tahir M.N. (2019). Crystalline network study of new *N*-phthaloyl-*β*-Alanine with benzimidazole, cocrystal: Computational consideration & free radical scavenging activity. J. Mol. Struct..

[54] De la Torre S.M.-D., Vázquez C., González-Chávez Z., Yépez-Mulia L., Nieto-Meneses R., Jasso-Chávez R., Saavedra E. (2017). Synthesis and biological evaluation of 2-methyl-1H-benzimidazole-5-carbohydrazides derivatives as modifiers of redox homeostasis of Trypanosoma cruzi. Bioorg. Med. Chem. Lett..

[55] Ozadali-Sari K., Küçükkılınç T.T., Ayazgok B., Balkan A., Unsal-Tan O. (2017). Novel multi-targeted agents for Alzheimer’s disease: Synthesis, biological evaluation, and molecular modeling of novel 2-[4-(4-substitutedpiperazin-1-yl) phenyl] benzimidazoles. Bioorg. Chem..

[56] Wang Y.-T., Qin Y.-J., Yang N., Zhang Y.-L., Liu C.-H., Zhu H.-L. (2015). Synthesis, biological evaluation, and molecular docking studies of novel 1-benzene acyl-2-(1-methylindol-3-yl)-benzimidazole derivatives as potential tubulin polymerization inhibitors. Eur. J. Med. Chem..

[57] Hauel N.H., Nar H., Priepke H., Ries U., Stassen J.M., Wienen W. (2002). Structure-based design of novel potent nonpeptide thrombin inhibitors. J. Med. Chem..

[58] Menteşe E., Bektaş H., Sokmen B.B., Emirik M., Çakır D., Kahveci B. (2017). Synthesis and molecular docking study of some 5,6-dichloro-2-cyclopropyl-1H-benzimidazole derivatives bearing triazole, oxadiazole, and imine functionalities as potent inhibitors of urease. Bioorg. Med. Chem. Lett..

[59] Toro P., Klahn A.H., Pradines B., Lahoz F., Pascual A., Biot C., Arancibia R. (2013). Organometallic benzimidazoles: Synthesis, characterization and antimalarial activity. Inorg. Chem. Commun..

[60] Rao A., Chimirri A., Clercq E.D., Monforte A.M., Monforte P., Pannecouque C., Zappala M. (2002). Synthesis and anti-HIV activity of 1-(2,6-difluorophenyl)-1H,3H-thiazolo[3,4-a]benzimidazole structurally-related 1,2-substituted benzimidazoles. IL Farm..

[61] Liao L., Jiang C., Chen J., Shi J., Li X., Wang Y., Wen J., Zhou S., Liang J., Lao Y. (2020). Synthesis and biological evaluation of 1,2,4-triazole derivatives as potential neuroprotectant against ischemic brain injury. Eur. J. Med. Chem..

[62] Groll A.H., Walsh T.J. (2002). Antifungal chemotherapy: Advances and perspectives. Swiss Med. Wkl..

[63] Xu Z., Zhao S.-J., Liu Y. (2019). 1,2,3-Triazole-containing hybrids as potential anticancer agents: Current developments, action mechanisms and structure-activity relationships. Eur. J. Med. Chem..

[64] Agisho H.A., Esatu H., Hairat S., Zaki M. (2020). TBHP/TBAI–Mediated simple and efficient synthesis of 3,5-disubstituted and 1,3,5-trisubstituted 1H-1,2,4-triazoles via oxidative decarbonylation of aromatic aldehydes and testing for antibacterial activities. Tetrahedron Lett..

[65] Mazur I., Belenichev I., Kucherenko L., Bukhtiyarova N., Khromylova O., Bidnenko O., Gorchakova N. (2019). Antihypertensive and cardioprotective effects of new compound 1-(β-phenylethyl)-4-amino-1,2,4-triazolium bromide (Hypertril). Eur. J. Pharm..

[66] Cao X., Wang W., Wang S., Bao L. (2017). Asymmetric synthesis of novel triazole derivatives and their *in vitro* antiviral activity and mechanism of action. Eur. J. Med. Chem..

[67] Kaproń B., Łuszczki J.J., Płazińska A., Siwek A., Karcz T., Gryboś A., Nowak G., Makuch-Kocka A., Walczak K., Langner E. (2019). Development of the 1,2,4-triazole-based anticonvulsant drug candidates acting on the voltage-gated sodium channels. Insights from *in-vivo*, *in-vitro*, and *in-silico* studies. Eur. J. Pharm. Sci..

[68] Zhou C.-H., Wang Y. (2012). Recent researches in triazole compounds as medicinal drugs. Curr. Med. Chem..

[69] Peyton L.R., Gallagher S., Hashemzadeh M. (2015). Triazole antifungals: A review. Drugs Today.

[70] Timur İ., Kocyigit Ü.M., Dastan T., Sandal S., Ceribası A.O., Taslimi P., Gulcin İ., Koparir M., Karatepe M., Çiftçi M.J. (2019). In vitro cytotoxic and in vivo antitumoral activities of some aminomethyl derivatives of 2,4-dihydro-3*H*-1,2,4-triazole-3-thiones—Evaluation of their acetylcholinesterase and carbonic anhydrase enzymes inhibition profiles. Biochem. Mol. Toxicol..

[71] Acharya P.T., Bhavsar Z.A., Jethava D.J., Patel D.B., Patel H.D. (2021). A review on development of bio-active thiosemicarbazide derivatives: Recent advances. J. Mol. Struct..

[72] Paytash P.L., Sparrow E., Gathe J.C. (1950). The reaction of itaconic acid with primary amines. J. Am. Chem. Soc..

[73] Brokaite K., Mickevicius V., Mikulskiene G. (2006). Synthesis and structural investigation of some 1,4-disubstituted-2-pyrrolidinones. ARKIVOC.

[74] Menteşe E., Sökmen B.B. (2019). Synthesis and *in vitro* urease inhibition of some novel benzimidazole-based hydrazones. J. Heterocycl. Chem..

[75] Balouiri M., Sadiki M., Ibnsouda S.K. (2016). Methods for in vitro evaluating antimicrobial activity: A review. J. Pharm. Anal..

[76] Parvekar P., Palaskar J., Metgud S., Maria R., Dutta S. (2020). The minimum inhibitory concentration (MIC) and minimum bactericidal concentration (MBC) of silver nanoparticles against Staphylococcus aureus. Biomater. Investig. Dent..

