# Supplementary material for: Synthesis and Antibacterial Activity of New Azole, Diazole and Triazole Derivatives Based on p-Aminobenzoic Acid
Source: Molecules. 2021 Apr 29;26(9):2597. doi: 10.3390/molecules26092597 (PMC8125559; doi:10.3390/molecules26092597)
Supplement: Supplementary file 1 [file molecules-26-02597-s001.zip › molecules-1170194-supplementary.pdf]

Synthesis and antibacterial activity of new azole, diazole and triazole derivatives based on *p*-aminobenzoic acid

by Birutė Sapijanskaitė-Banevič, Vykintas Palskys, Rita Vaickelionienė*, Jūratė Šiugždaitė, Povilas Kavaliauskas, Birutė Grybaitė, and Vytautas Mickevičius

SUPPORTING INFORMATION

- **NMR Spectra (2-24) (All in DMSO-d_6)**
- **Bacteria strains used in the biological evaluation**

Synthesis of 5-oxopyrrolidine derivatives 2-13

1-(4-Carboxyphenyl)-5-oxopyrrolidine-3-carboxylic acid 2

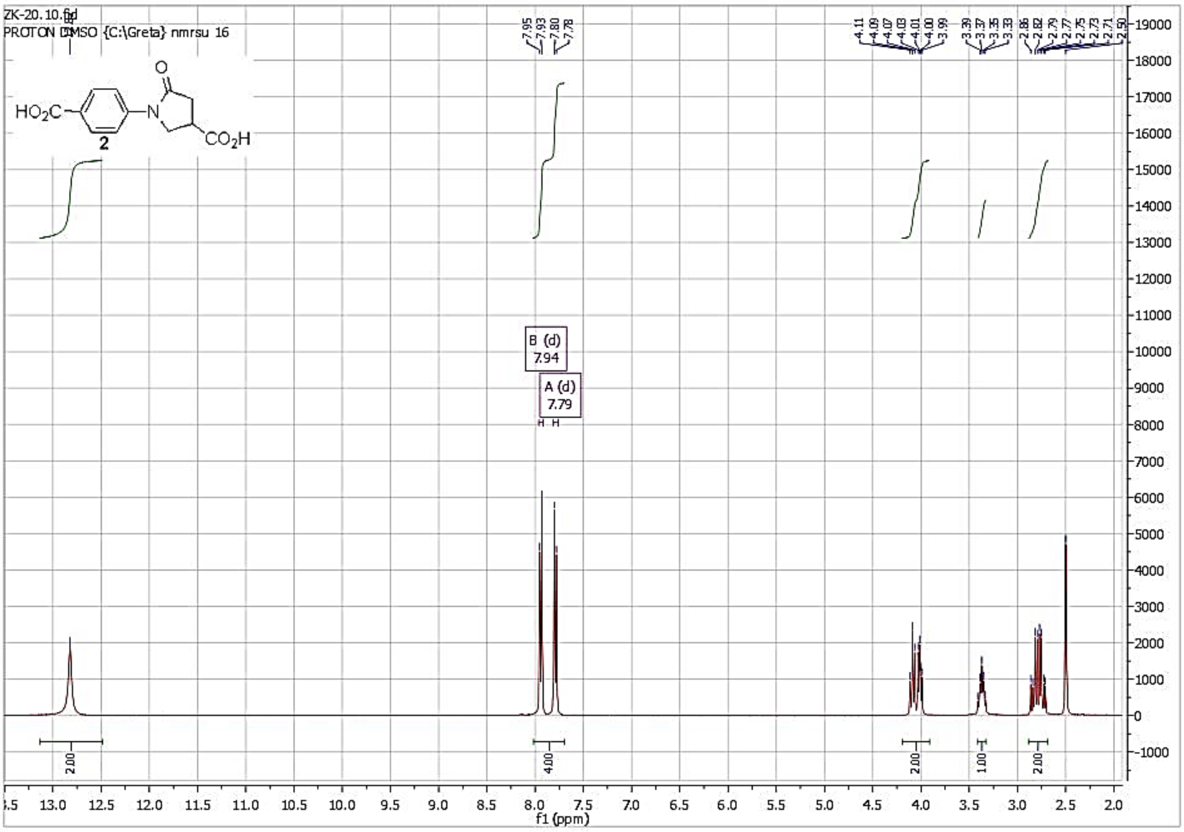

Figure S1: *^{1}H NMR of compound* **2**

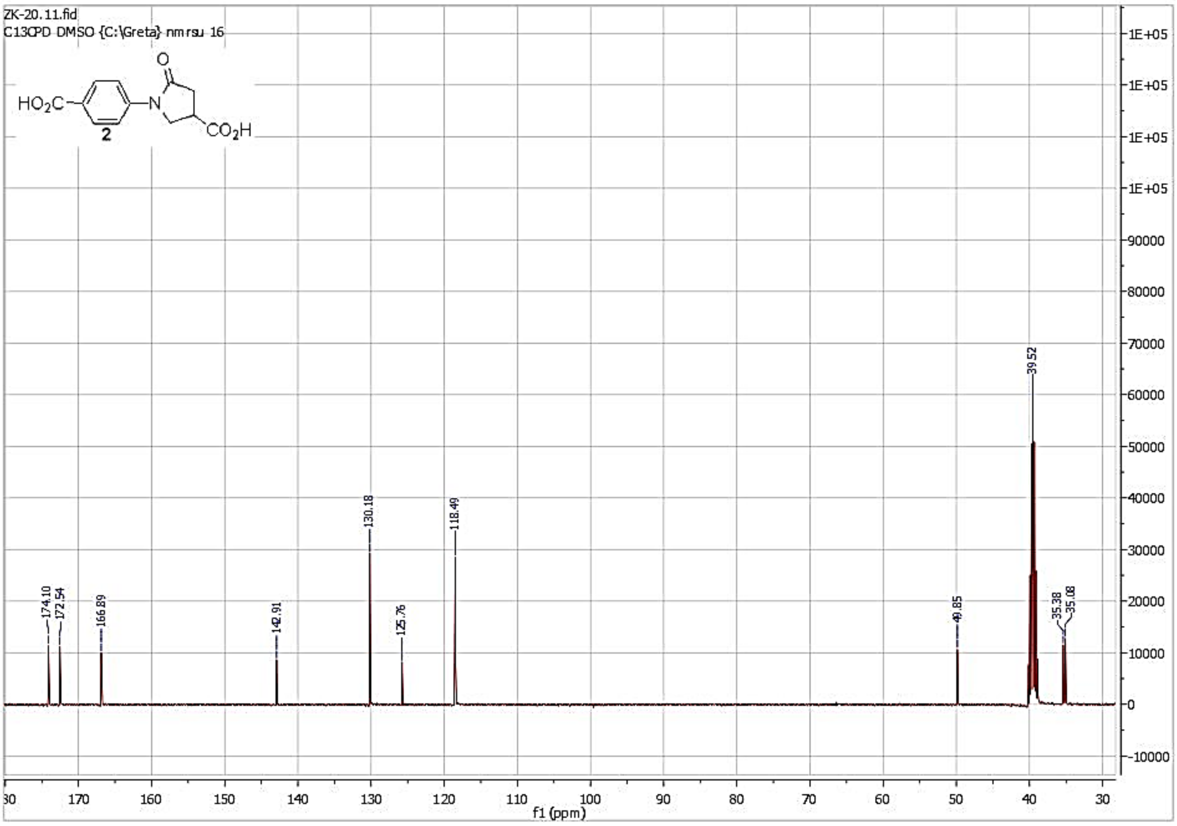

Figure S2: *^{13}C NMR of compound* **2**

Methyl 1-[4-(methoxycarbonyl)phenyl]-5-oxopyrrolidine-3-carboxylate 3

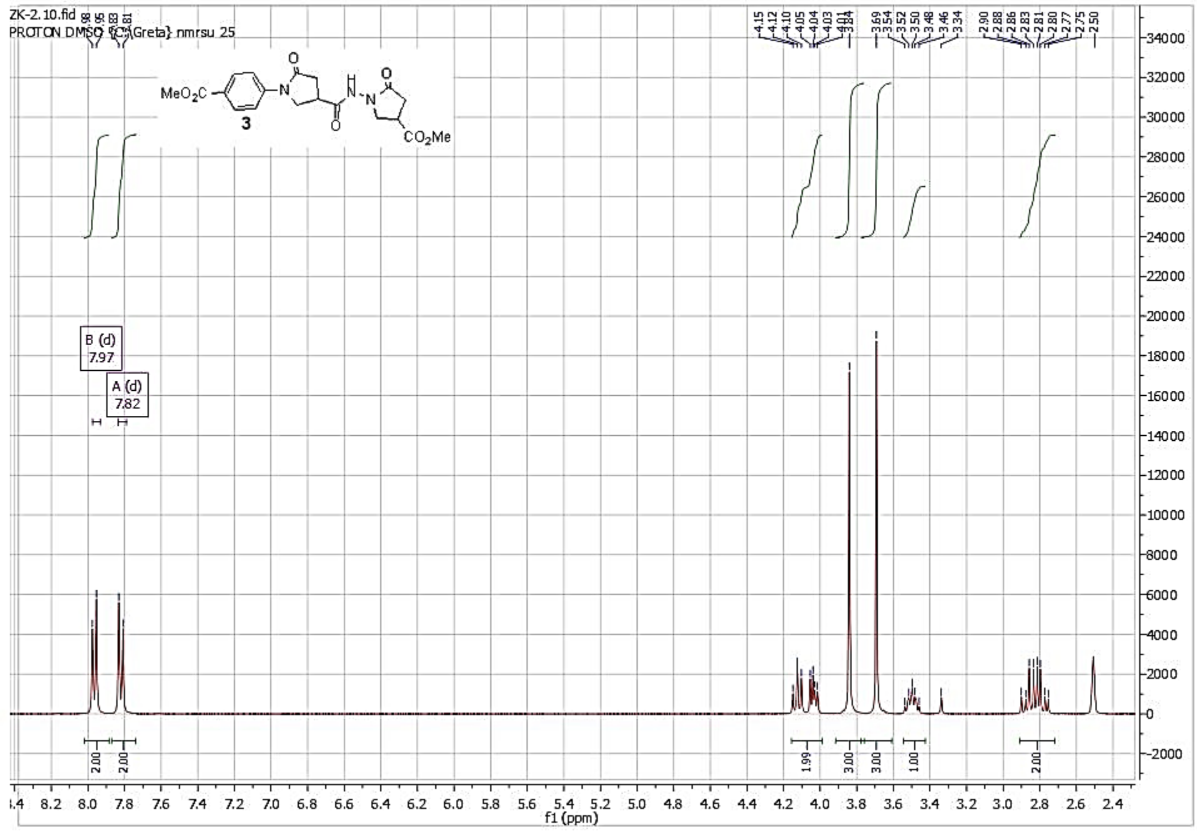

Figure S3: *^{1}H NMR of compound* ***3***

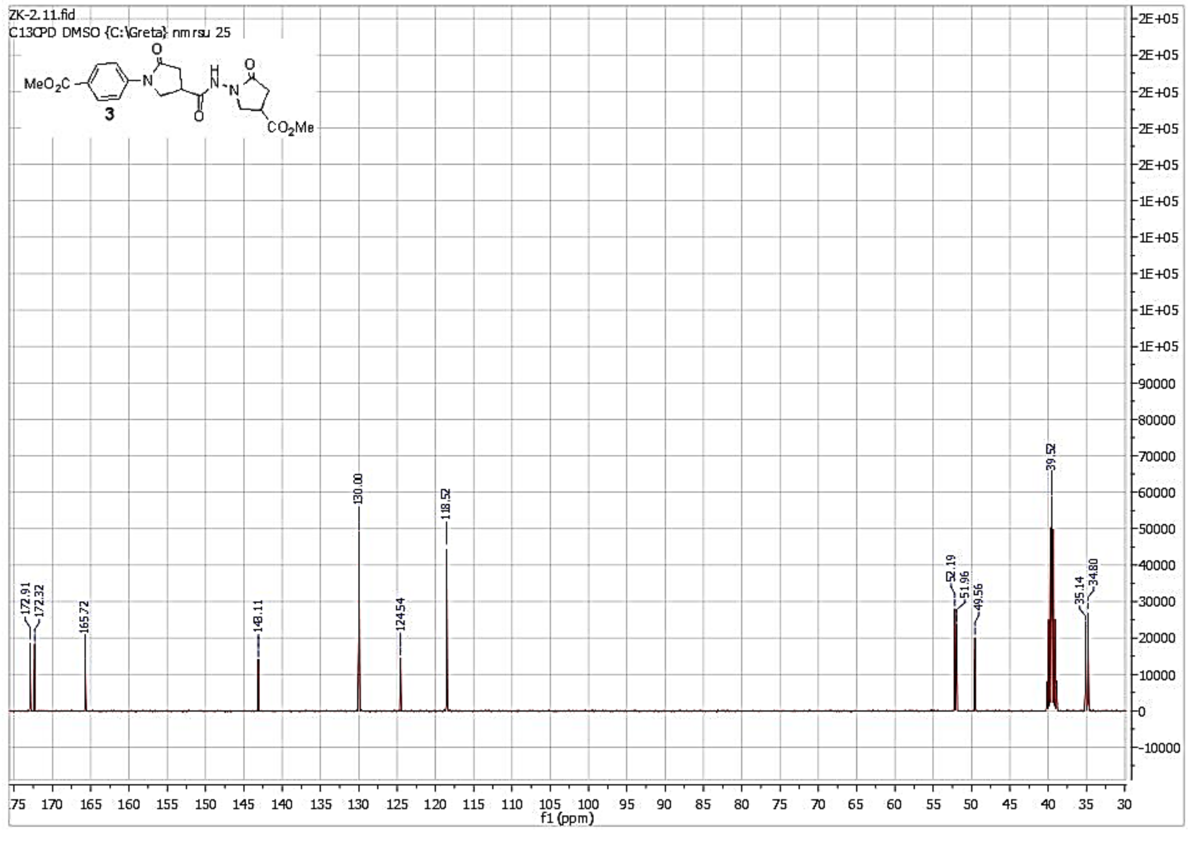

Figure S4: *^{13}C NMR of compound* ***3***

Methyl 4-[3-(hydrazinocarbonyl)-5-oxopyrrolidin-1-yl]benzoate 4

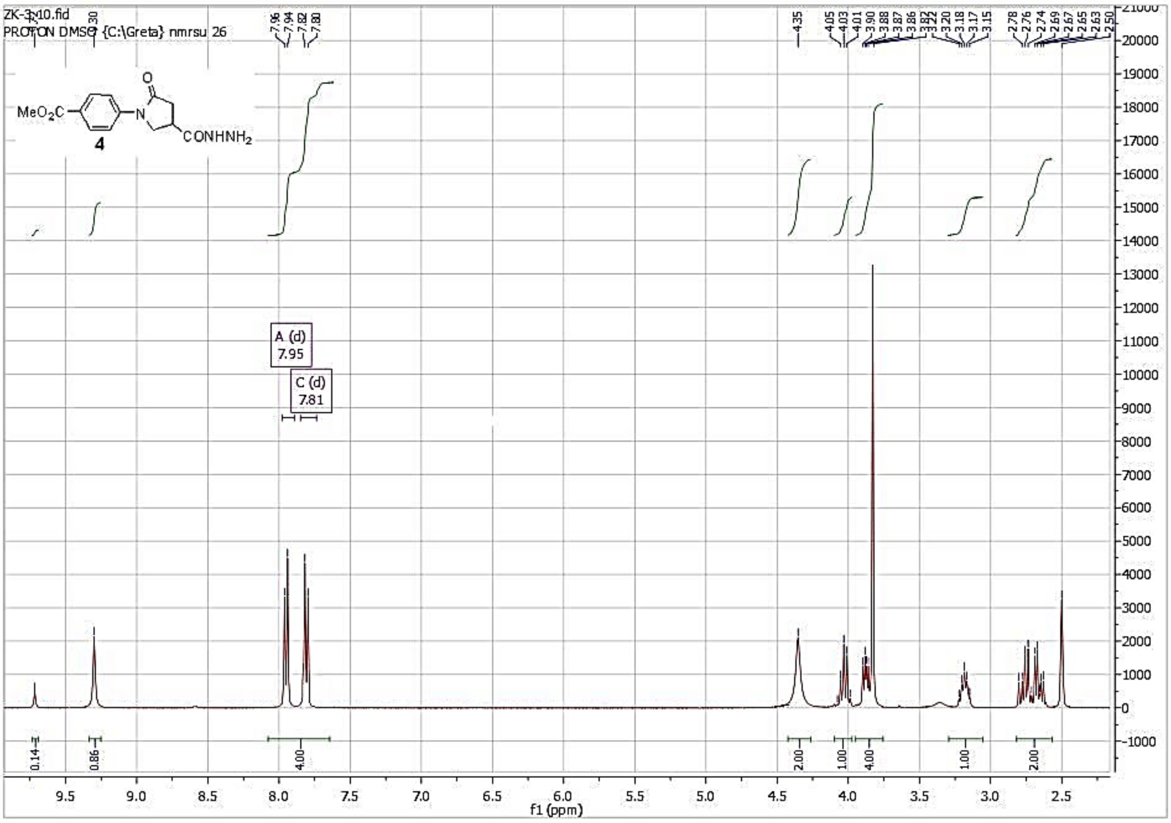

Figure S5: *^{1}H NMR of compound* ***4***

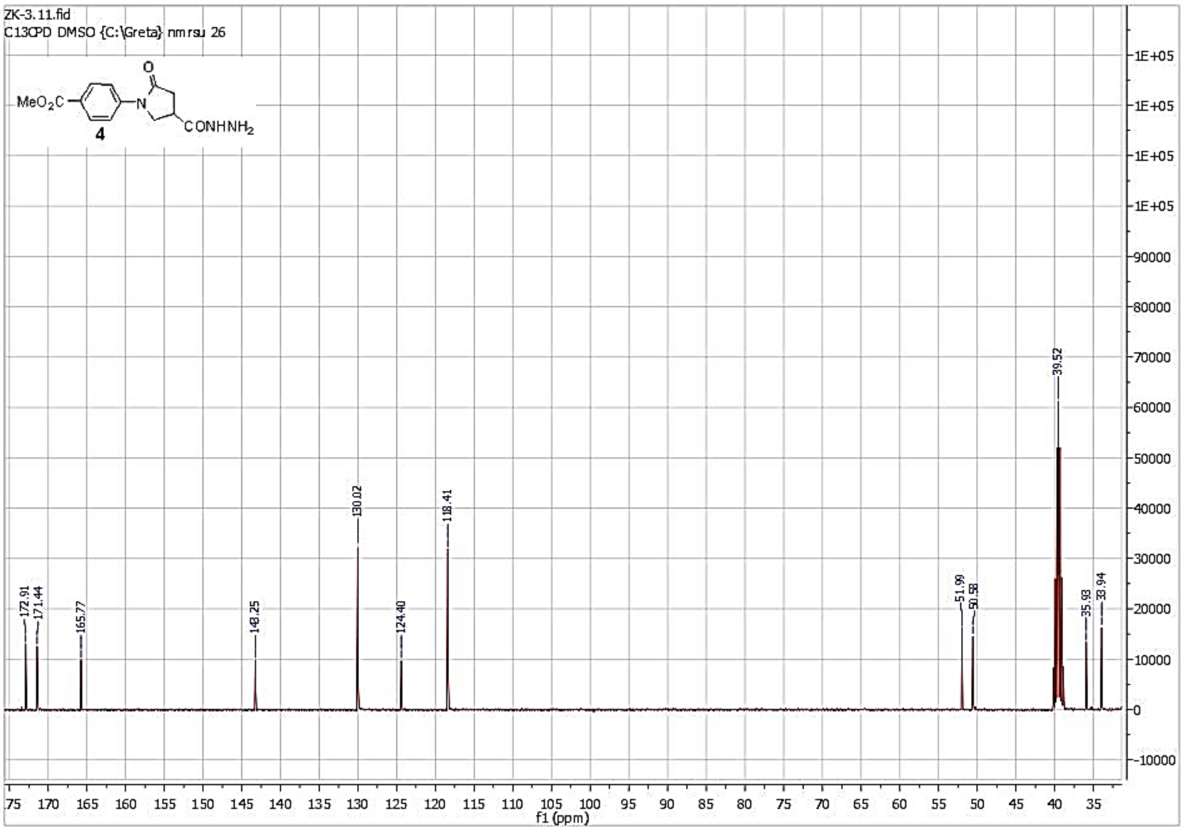

Figure S6: *^{13}C NMR of compound* ***4***

Methyl 4-(4-(3,5-dimethyl-1H-pyrazole-1-carbonyl)-2-oxopyrrolidin-1-yl)benzoate 5

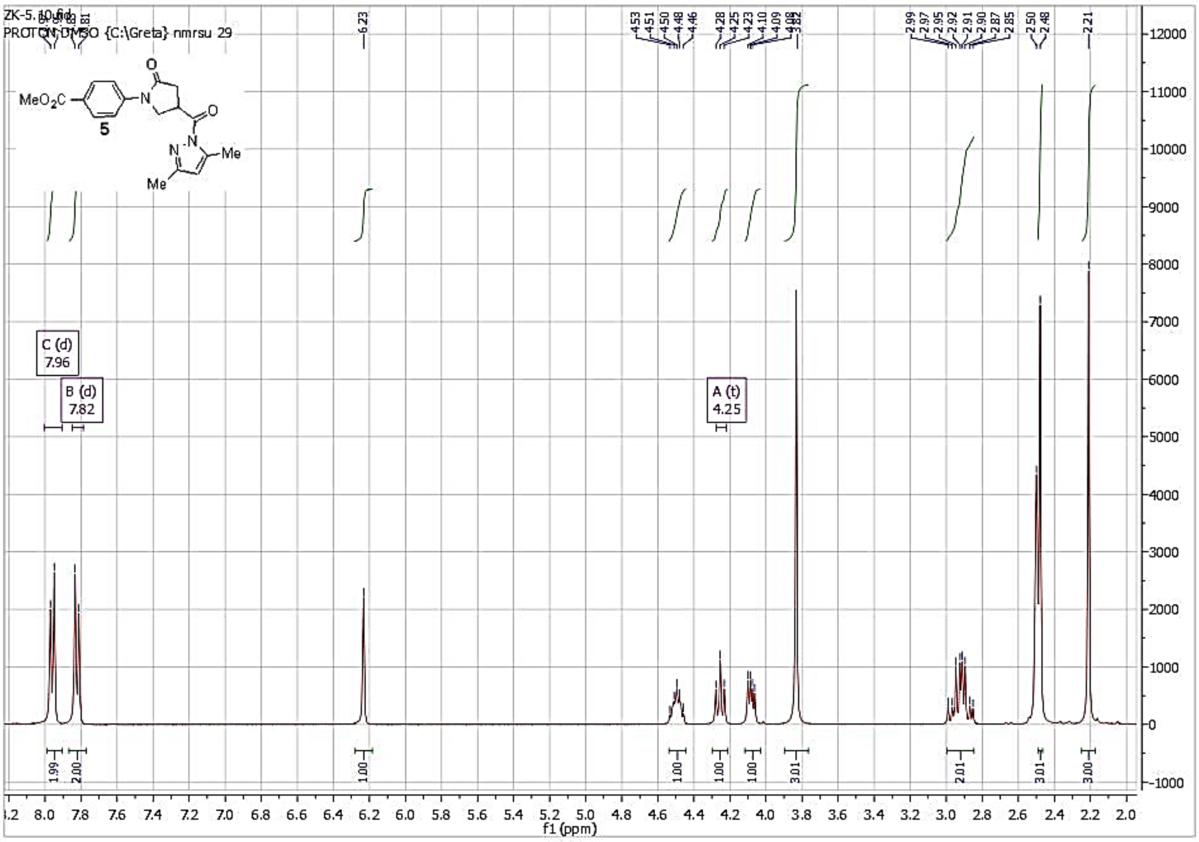

Figure S7: *^{1}H NMR of compound **5***

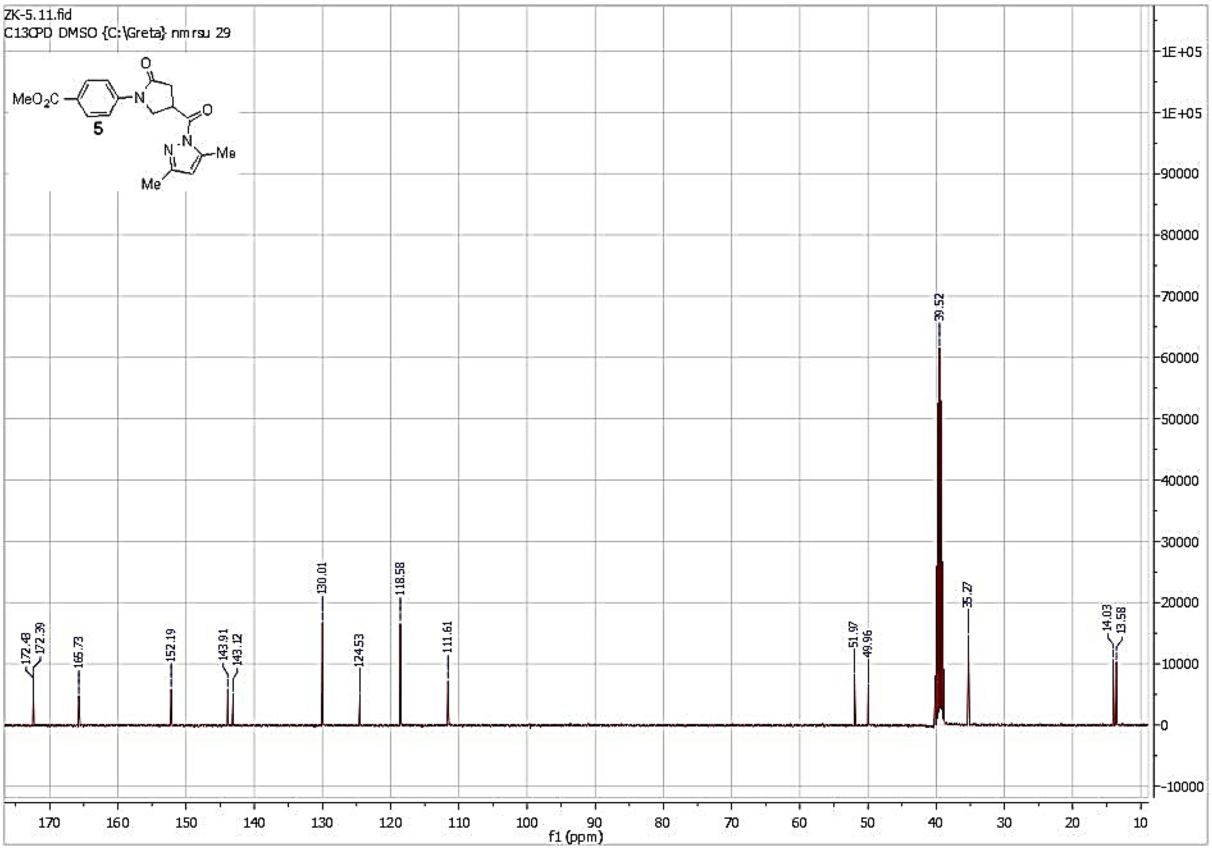

Figure S8: *^{13}C NMR of compound **5***

Methyl 4-(4-((2,5-dimethyl-1H-pyrrol-1-yl)carbamoyl)-2-oxopyrrolidin-1-yl)benzoate 6

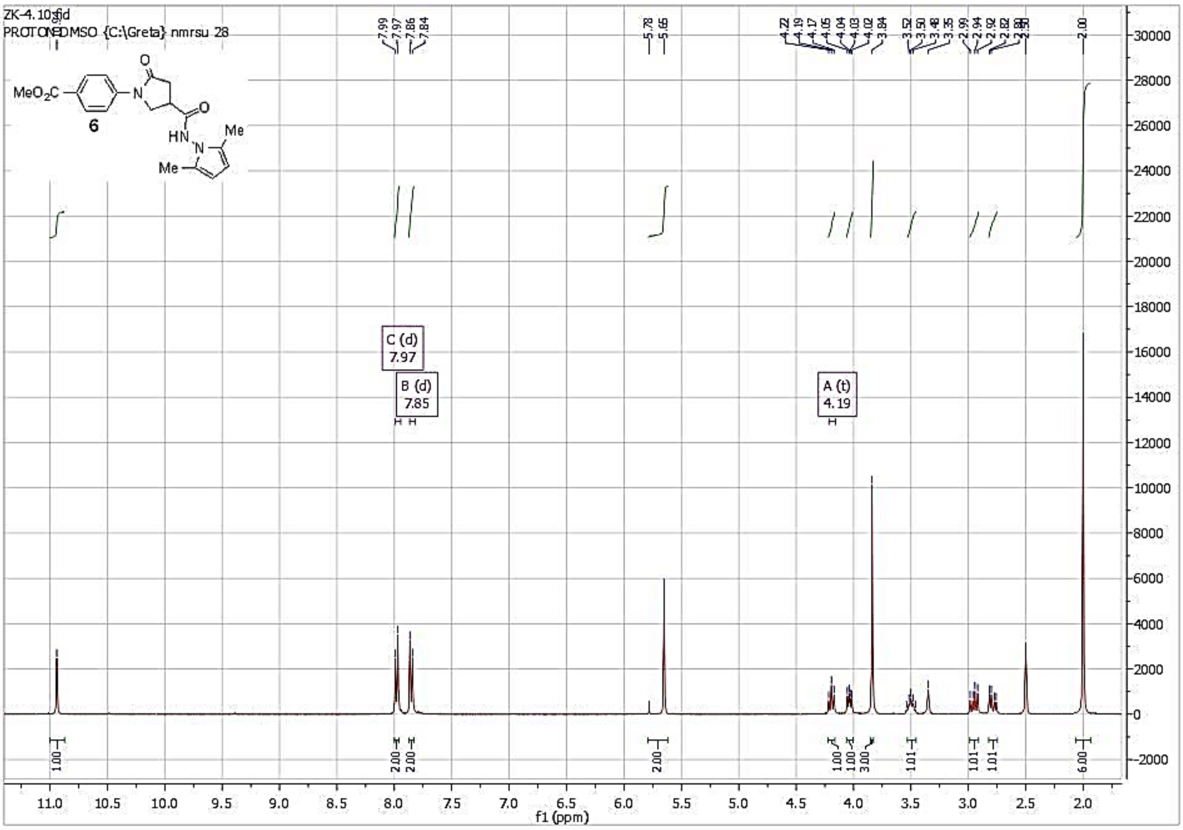

Figure S9: *^{1}H NMR of compound **6***

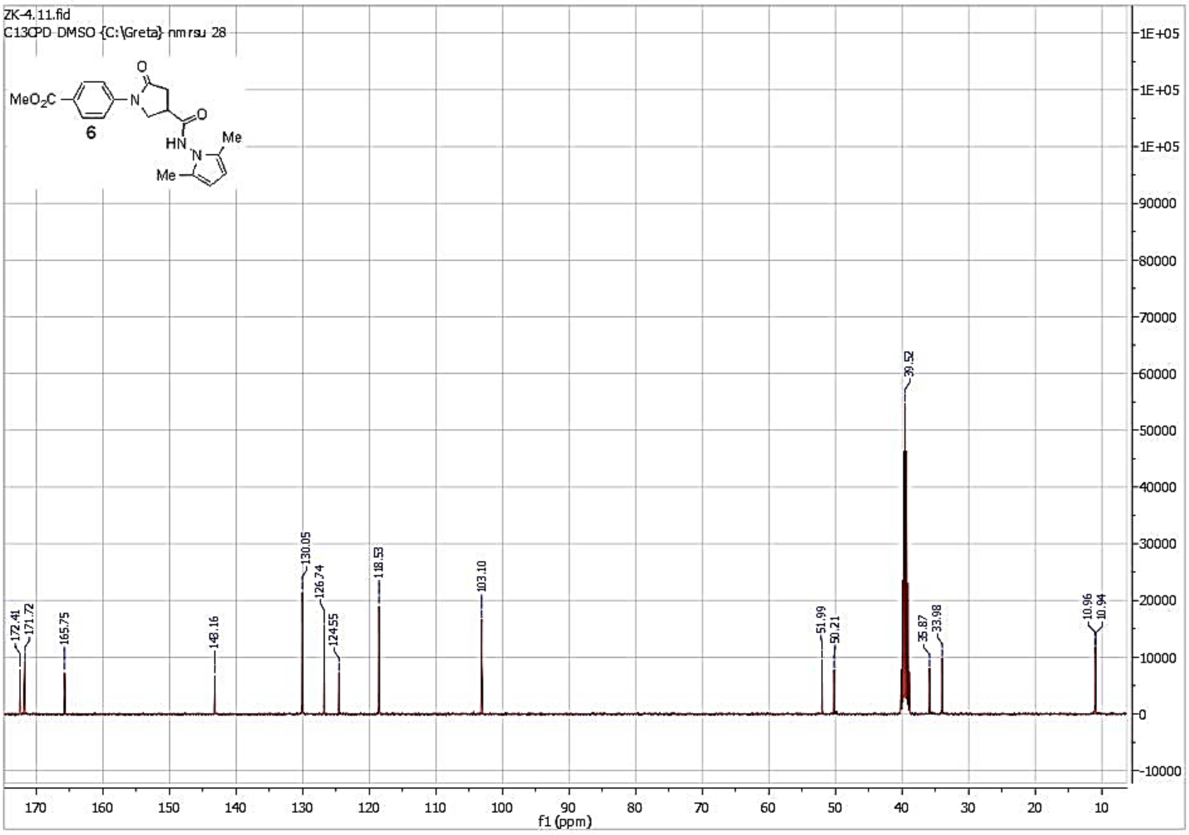

Figure S10: *^{13}C NMR of compound **6***

Methyl 4-(4-(2-benzylidenehydrazine-1-carbonyl)-2-oxopyrrolidin-1-yl)benzoate 7a

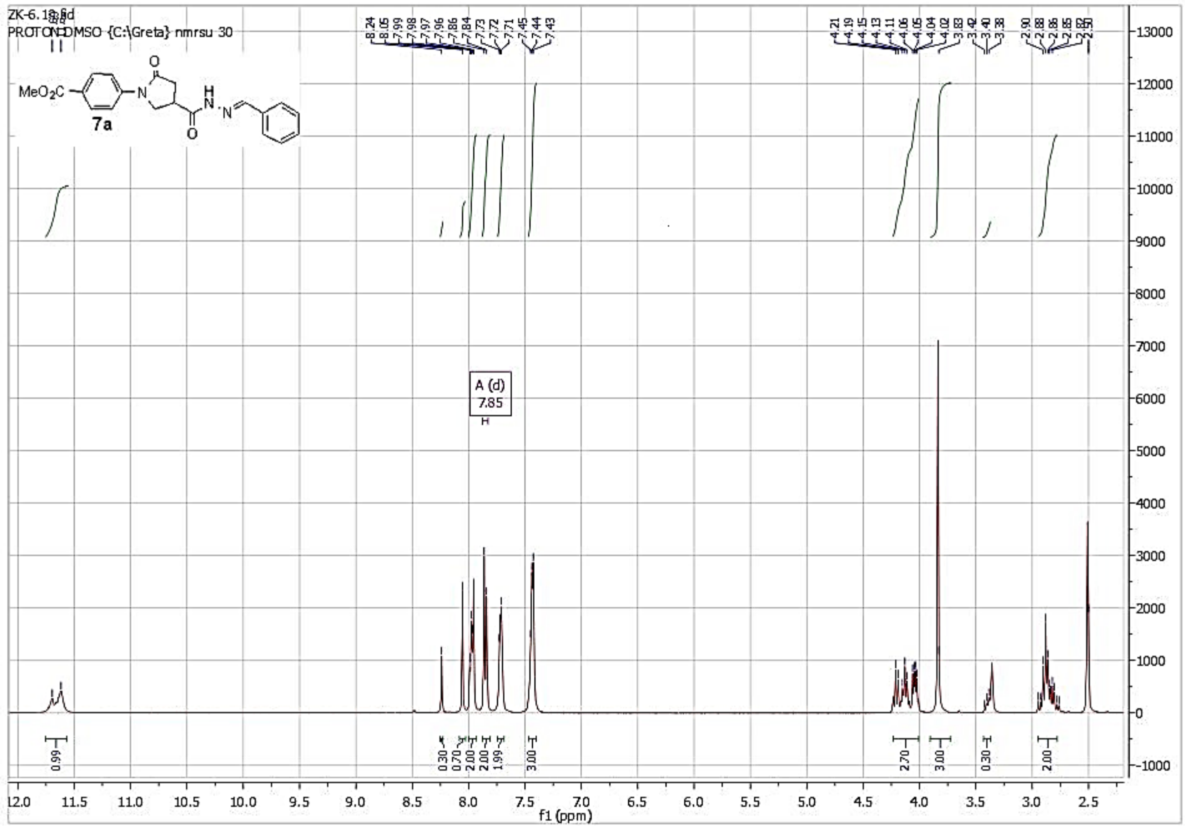

Figure S11: *^{1}H NMR of compound* ***7a***

Methyl 4-(4-(2-(4-methoxybenzylidene)hydrazine-1-carbonyl)-2-oxopyrrolidin-1-yl)benzoate 7b

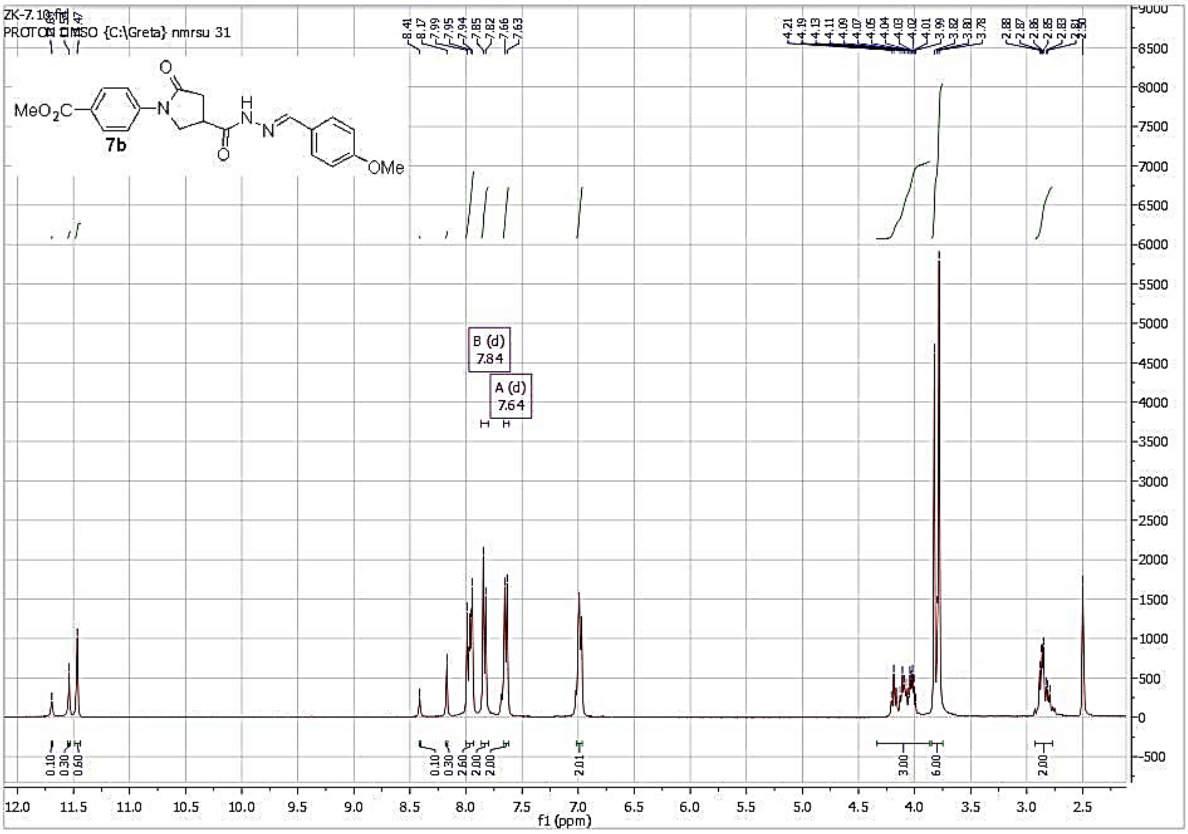

Figure S12: *^{1}H NMR of compound* ***7b***

Methyl 4-(4-(2-(4-(dimethylamino)benzylidene)hydrazine-1-carbonyl)-2-oxopyrrolidin-1-yl)benzoate 7c

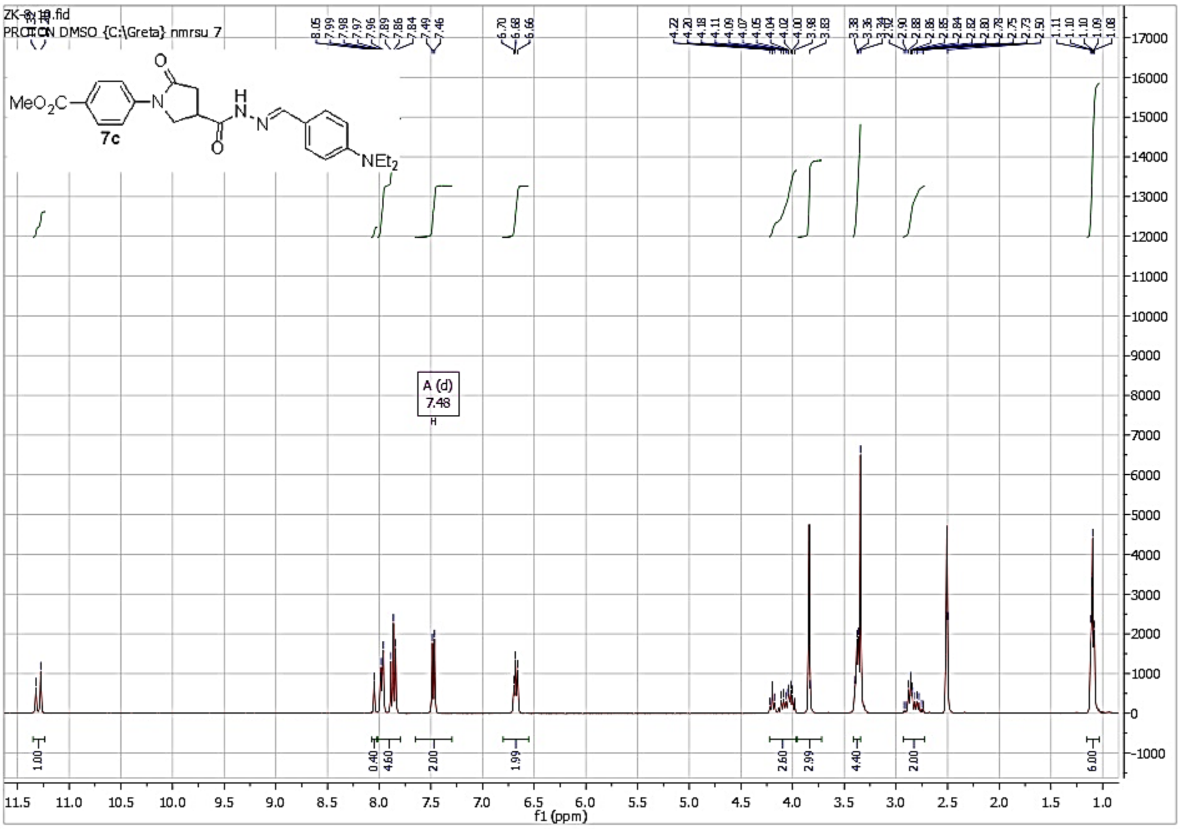

Figure S13: *^{1}H NMR of compound* ***7c***

1-(1-(4-(Methoxycarbonyl)phenyl)-5-oxopyrrolidine-3-carboxamido)-5-oxopyrrolidine-3-carboxylic acid 8

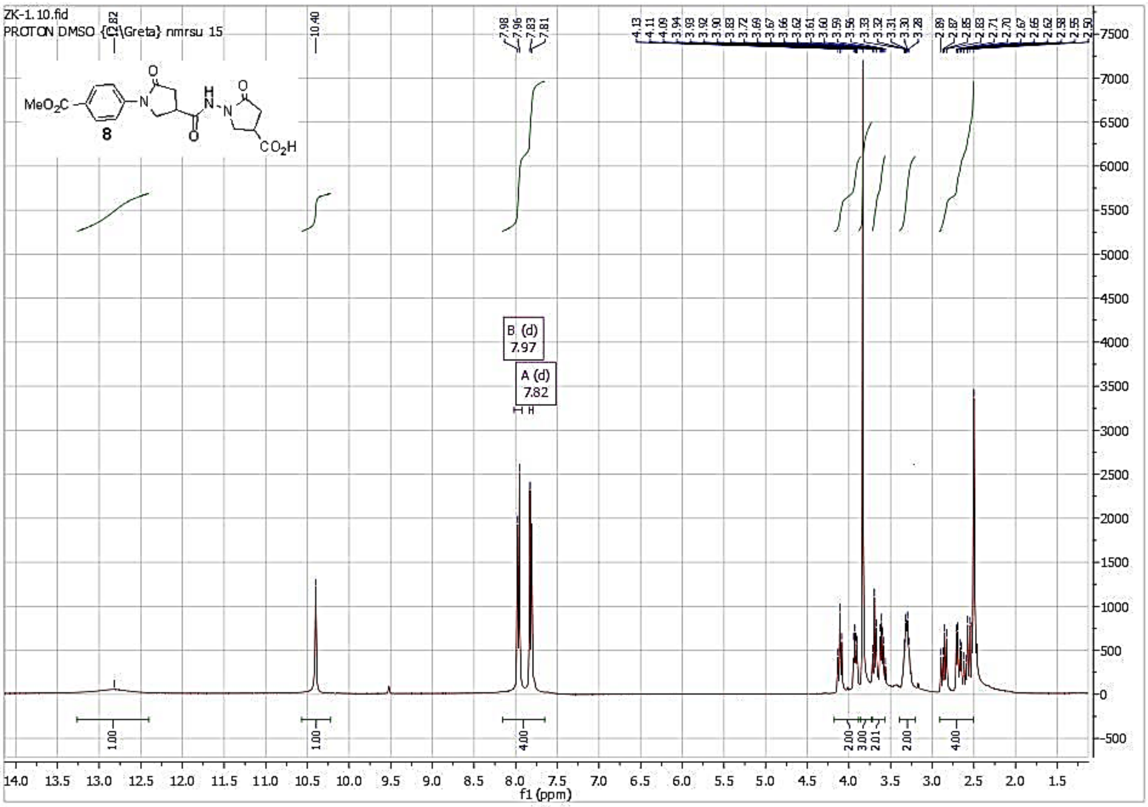

Figure S14: *^{1}H NMR of compound* ***8***

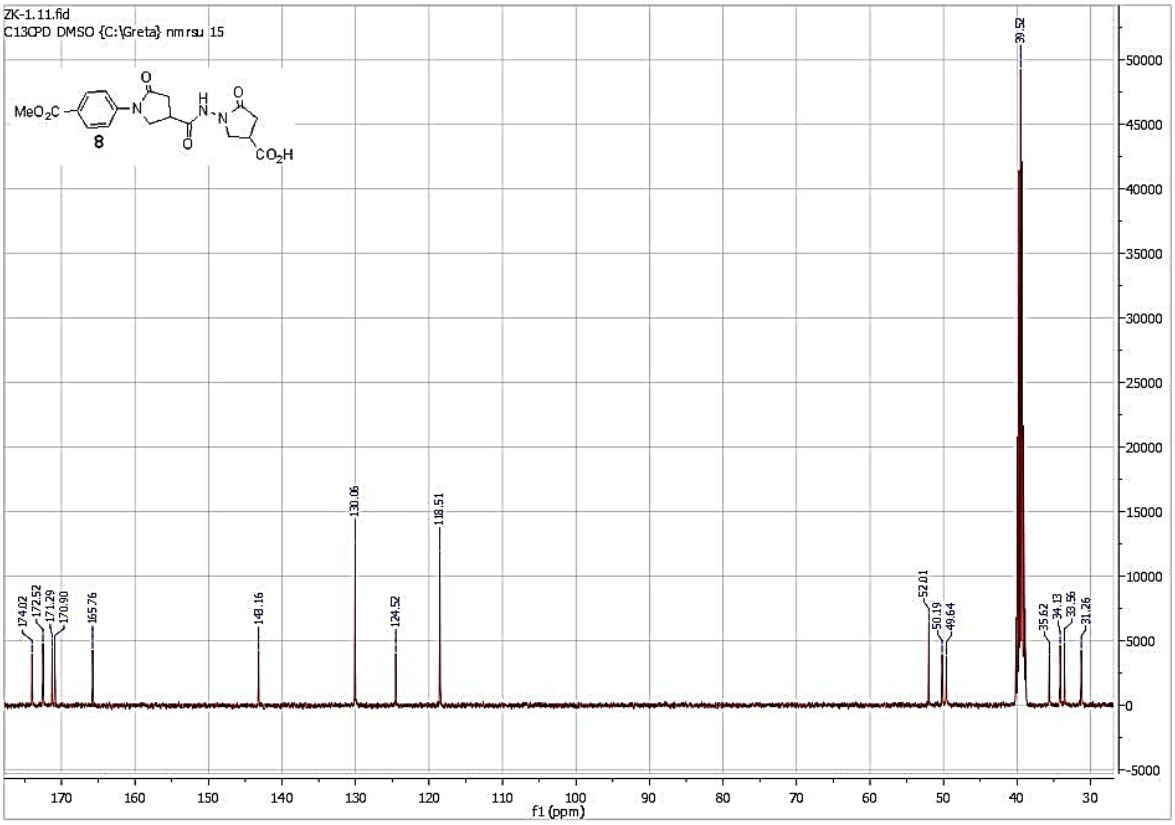

Figure S15: *^{13}C NMR of compound* ***8***

Methyl 4-(4-(2-benzoylhydrazine-1-carbonyl)-2-oxopyrrolidin-1-yl)benzoate 9

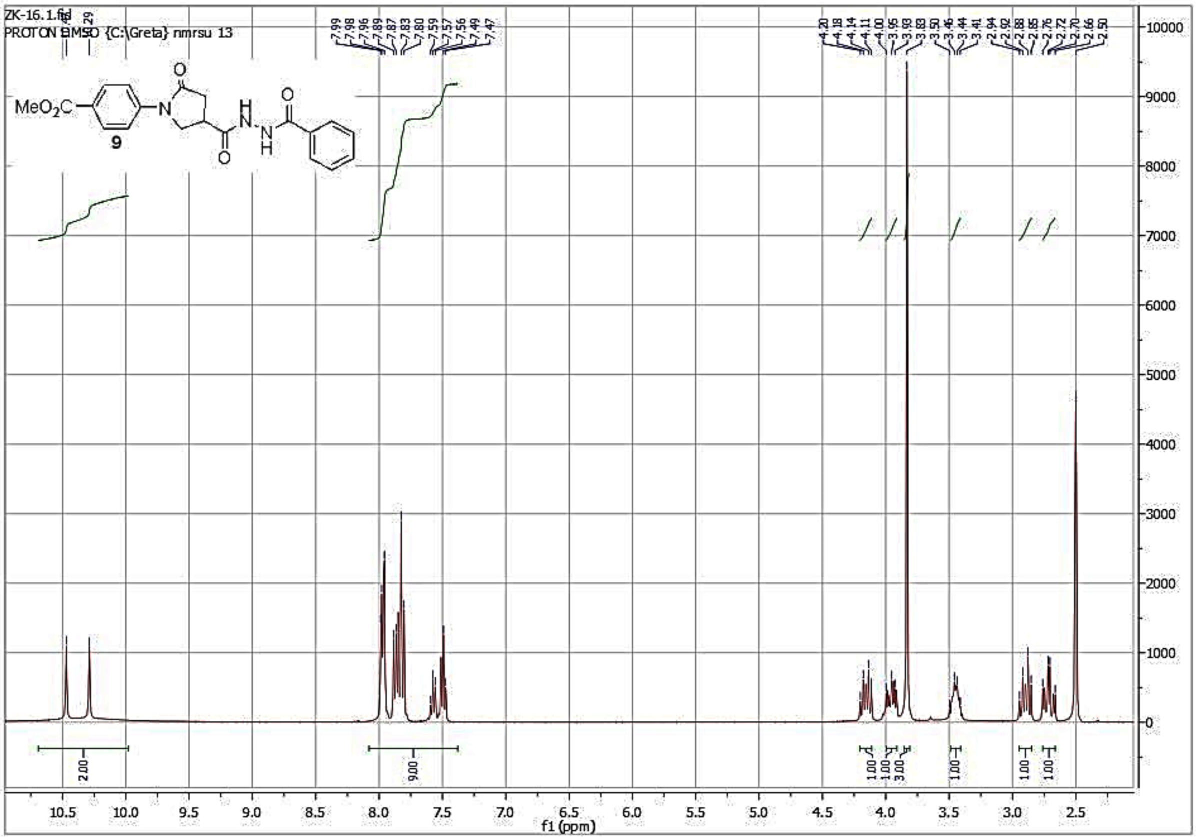

Figure S16: ^{1}*H NMR of compound* ***9***

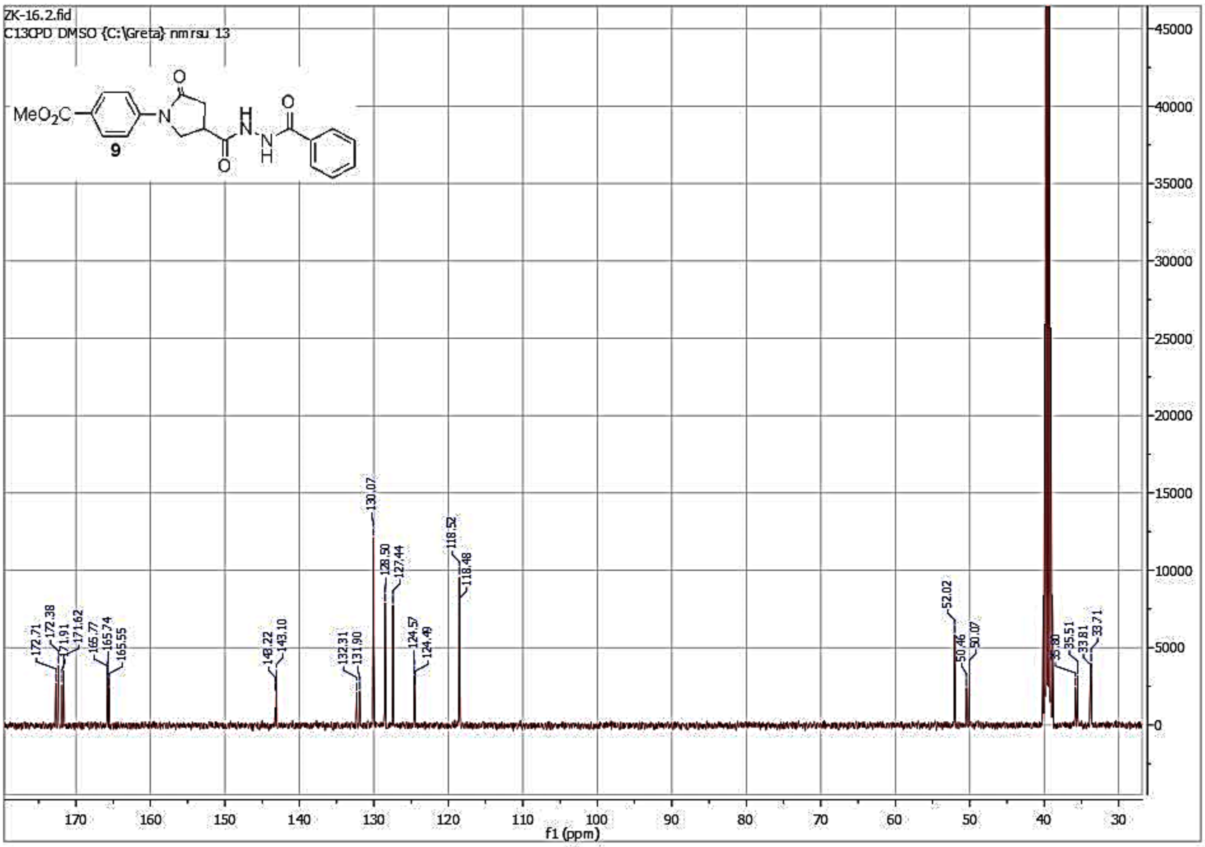

Figure S17: ^{13}*C NMR of compound* ***9***

1-(4-(Hydrazinecarbonyl)phenyl)-5-oxopyrrolidine-3-carbohydrazide 10

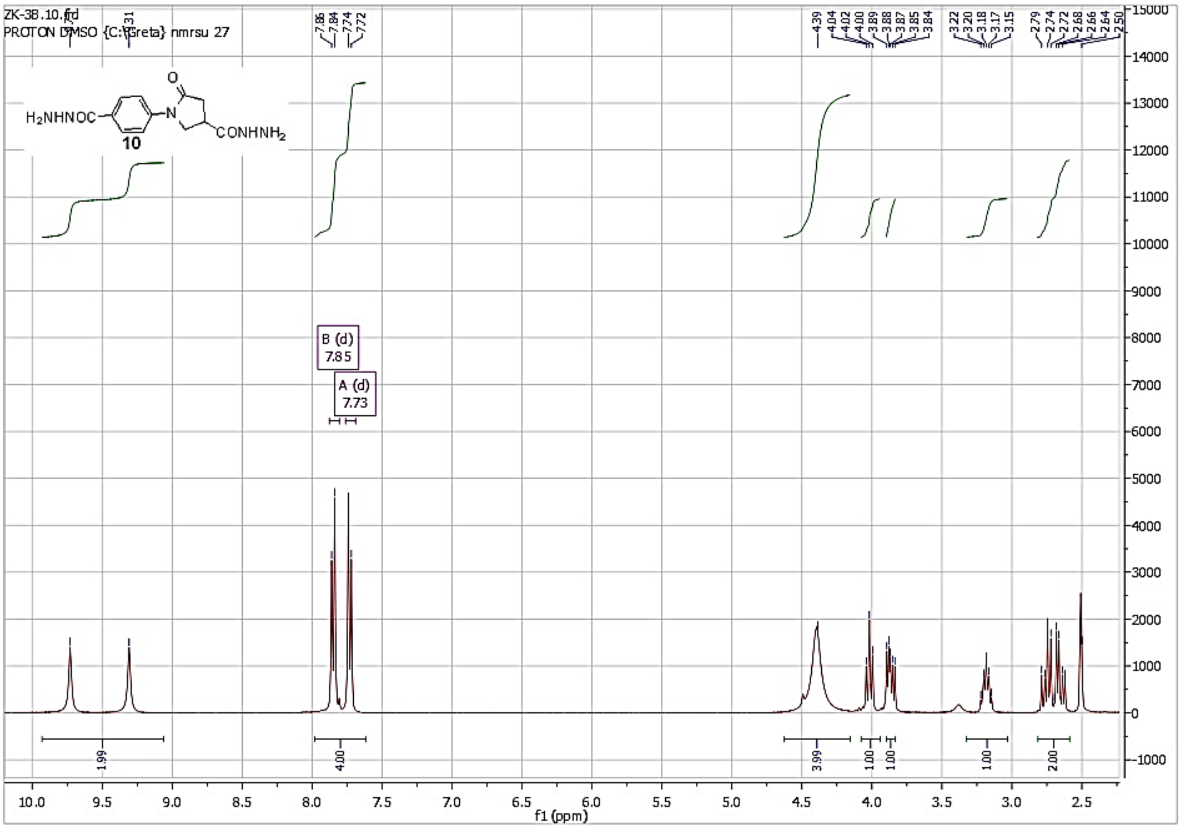

Figure S18: ^{1}H NMR of compound **10**

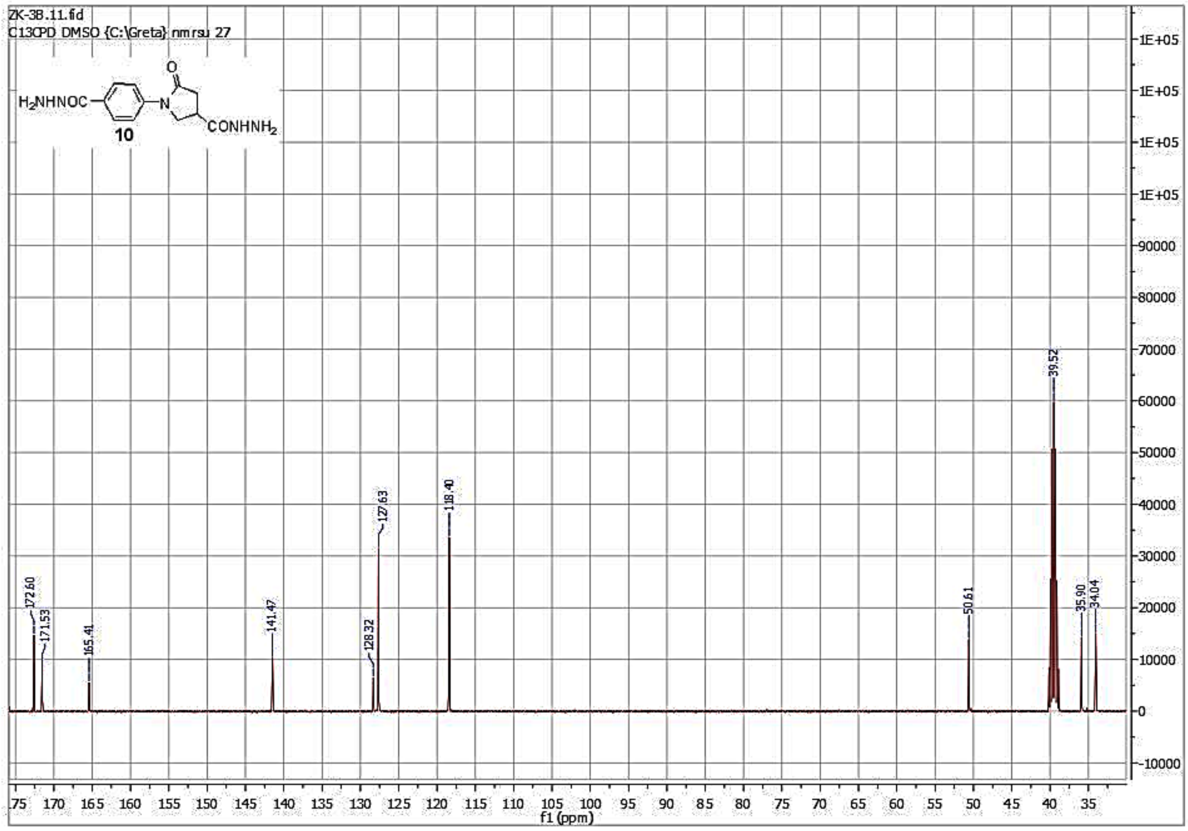

Figure S19: ^{13}C NMR of compound **10**

N-(4-nitrobenzylidene)-1-[4-[2-(4-nitrobenzylidenehydrazinocarbonyl)]phenyl]-5-oxopyrrolidine-3-carbohydrazide 11a

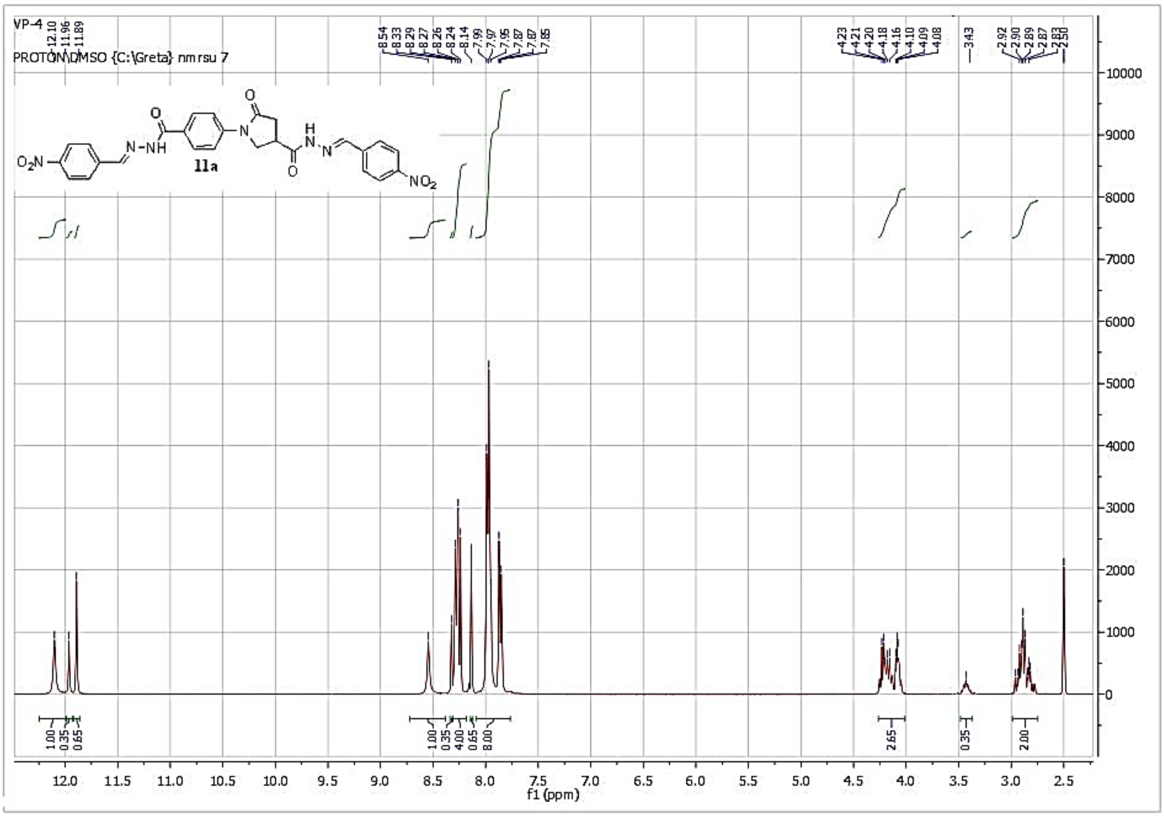

Figure S20: ^{1}H NMR of compound ***11a***

N-(4-chlorobenzylidene)-1-[4-[2-(4-chlorobenzylidenehydrazinocarbonyl)]phenyl]-5-oxopyrrolidine-3-carbohydrazide 11b

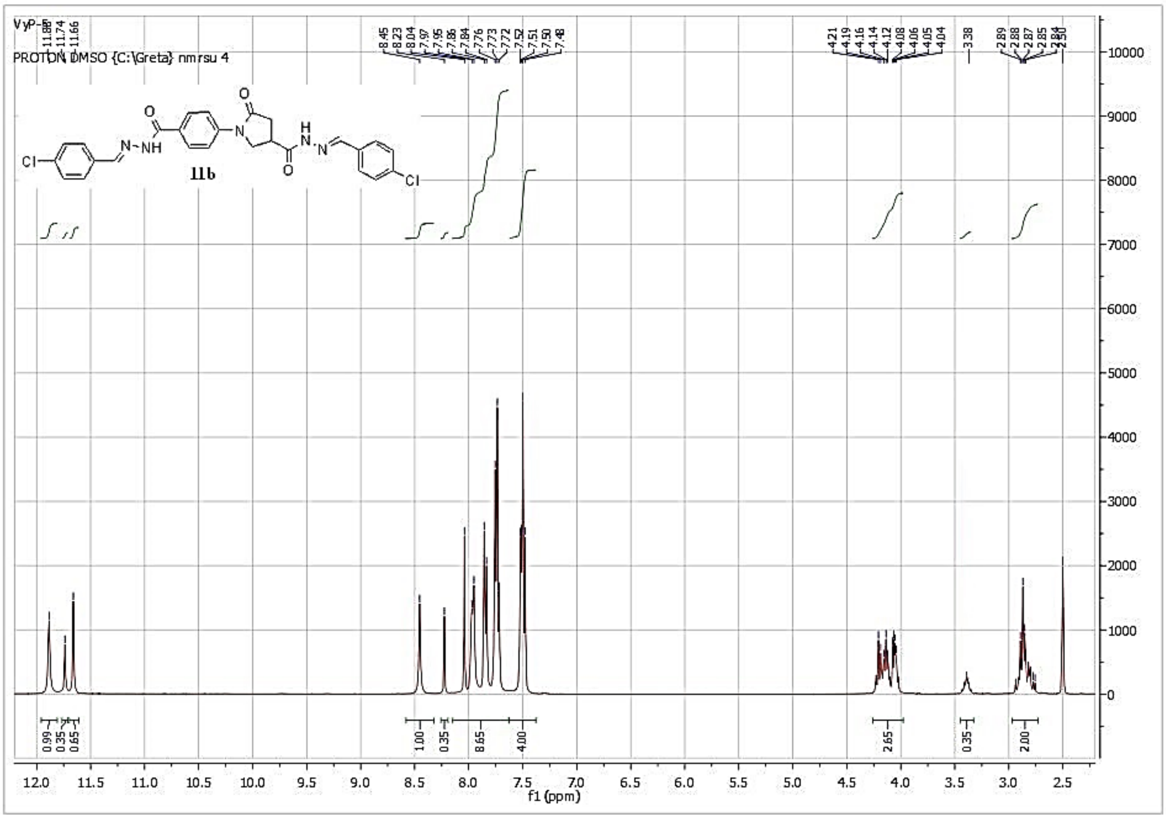

Figure S21: ^{1}H NMR of compound ***11b***

1-[[5-Oxo-1-(4-[(phenylcarbamoylthioamino)carbamoyl]pyrrolidin-3-carbonyl]-3-phenylthiocarbamide 12

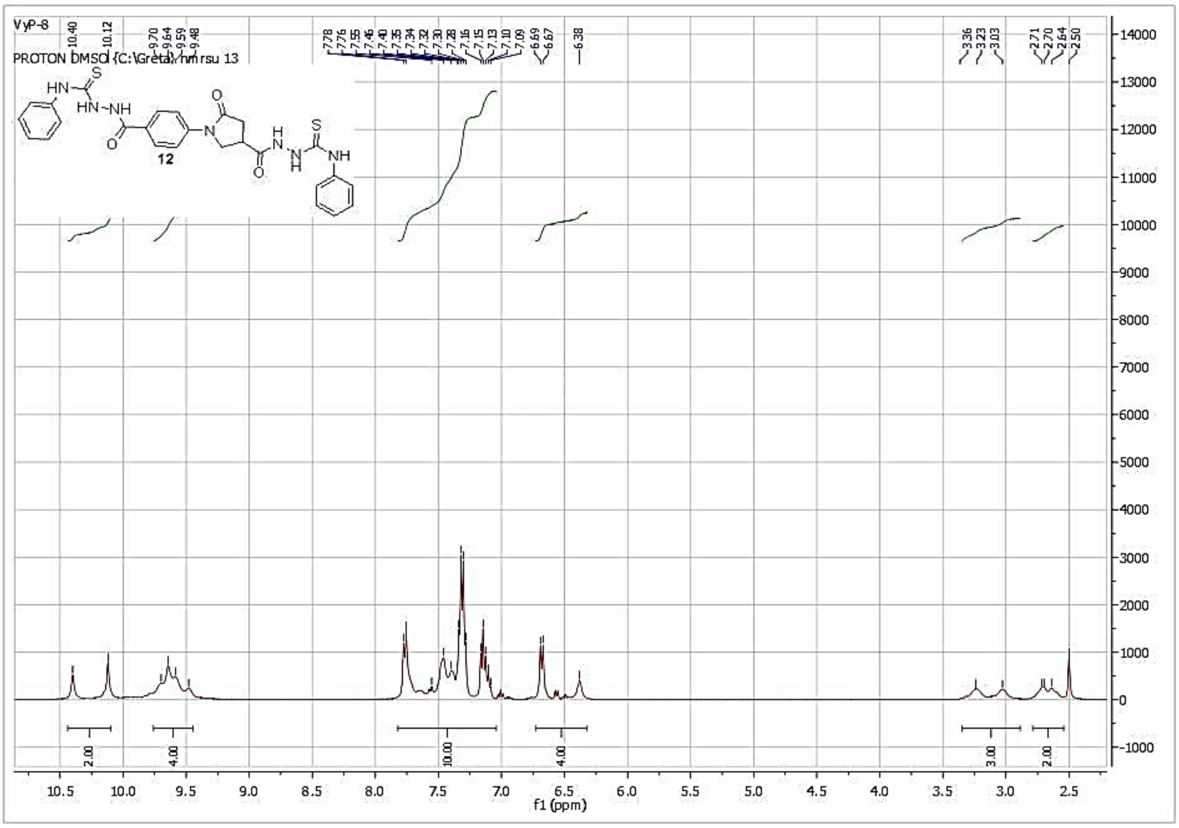

Figure S22: ^{1}H NMR of compound **12**

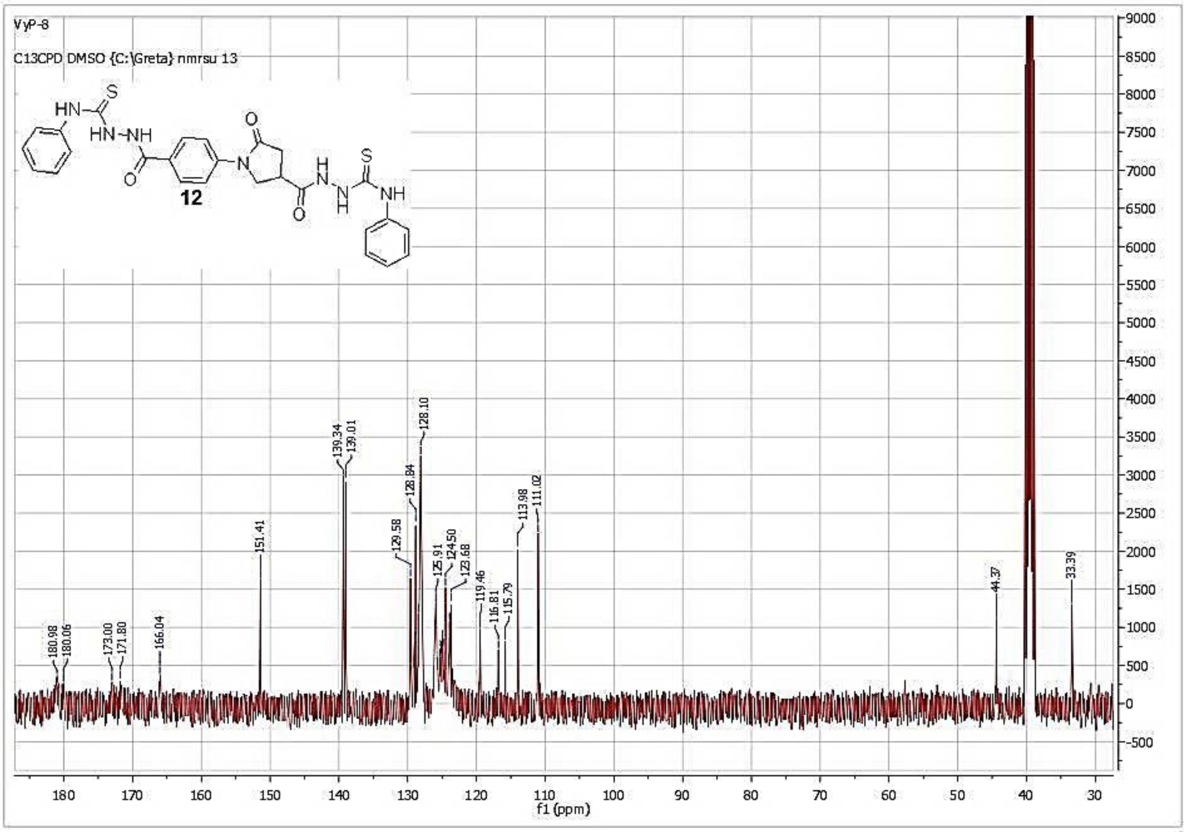

Figure S23: ^{13}C NMR of compound **12**

4-(4-phenyl-5-thioxo-4,5-dihydro-1H-1,2,4-triazol-3-yl)-1-(4-(4-phenyl-5-thioxo-4,5-dihydro-1H-1,2,4-triazol-3-yl)phenyl)pyrrolidin-2-one 13

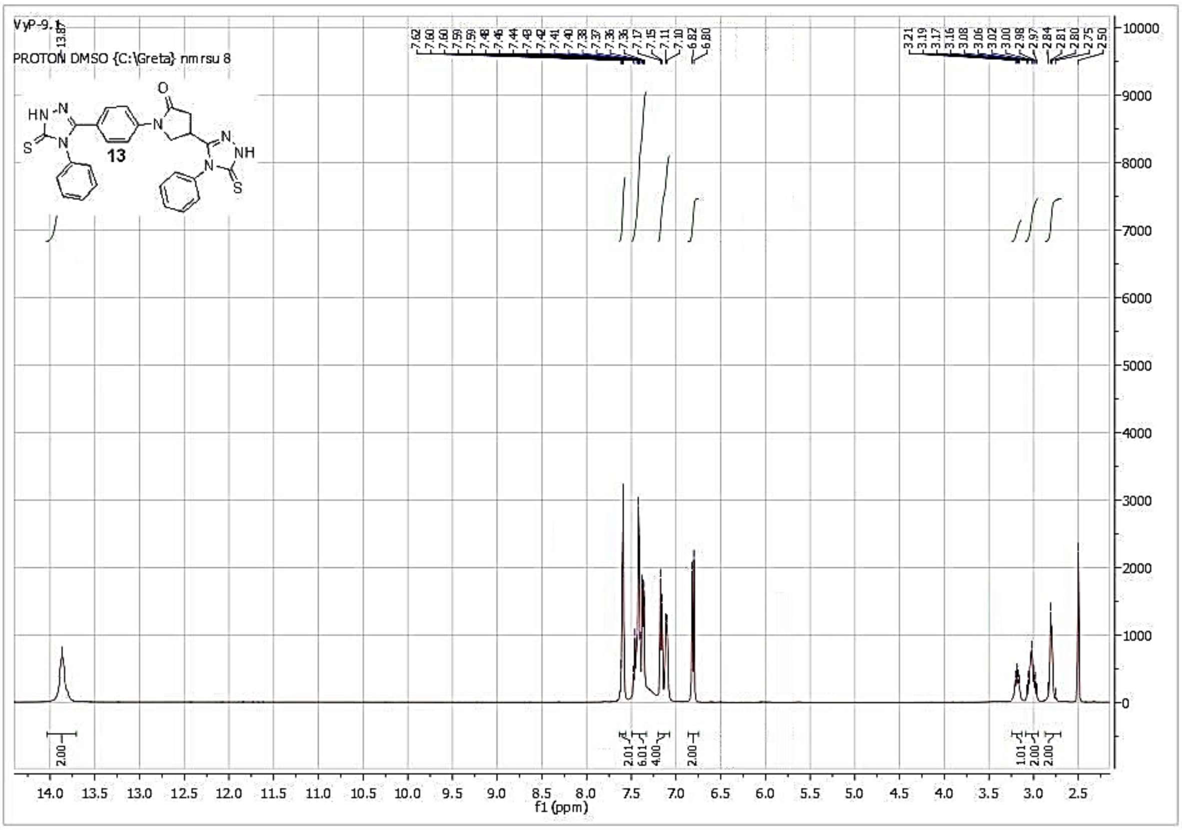

Figure S24: *^{1}H NMR of compound* ***13***

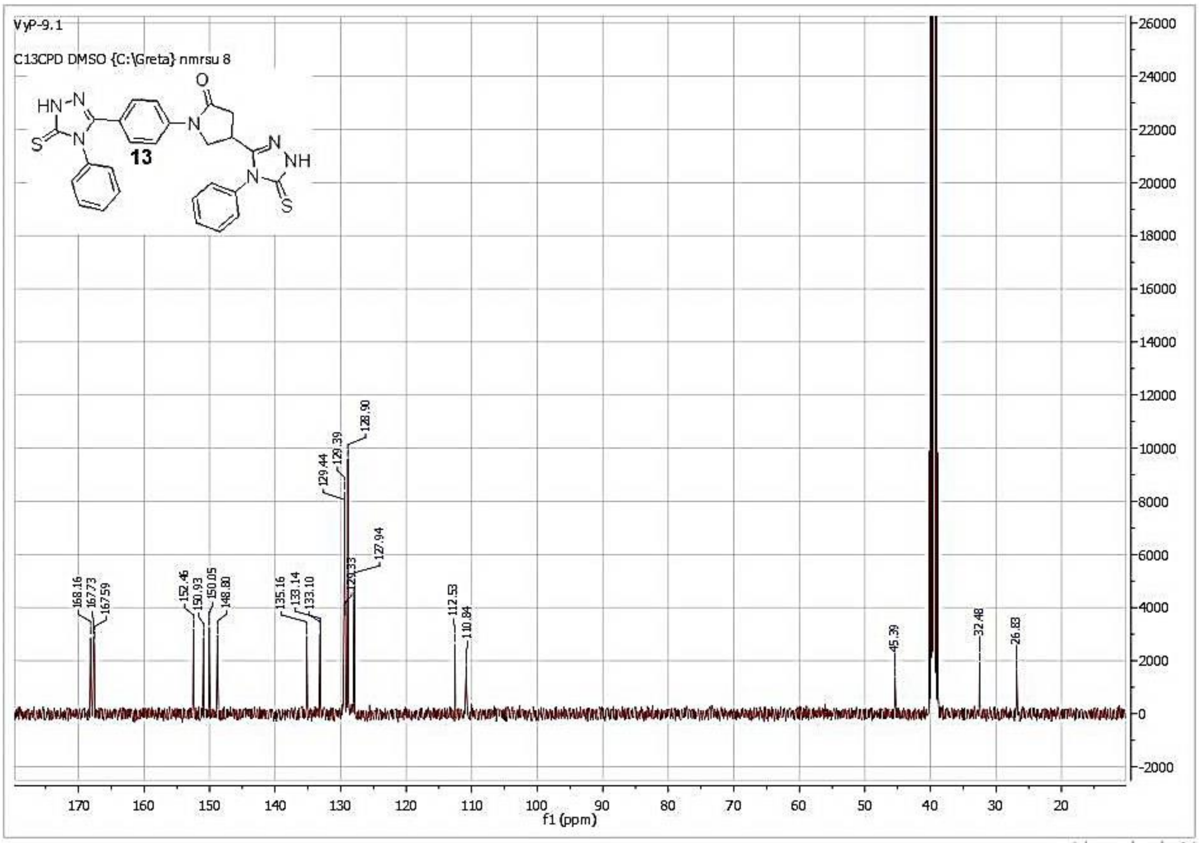

Figure S25: *^{13}C NMR of compound* ***13***

Synthesis of benzimidazoles 14-18

3-(1H-benzimidazol-2-yl)-1-[4-(1H-benzimidazol-2-yl)phenyl]pyrrolidin-5-one 14

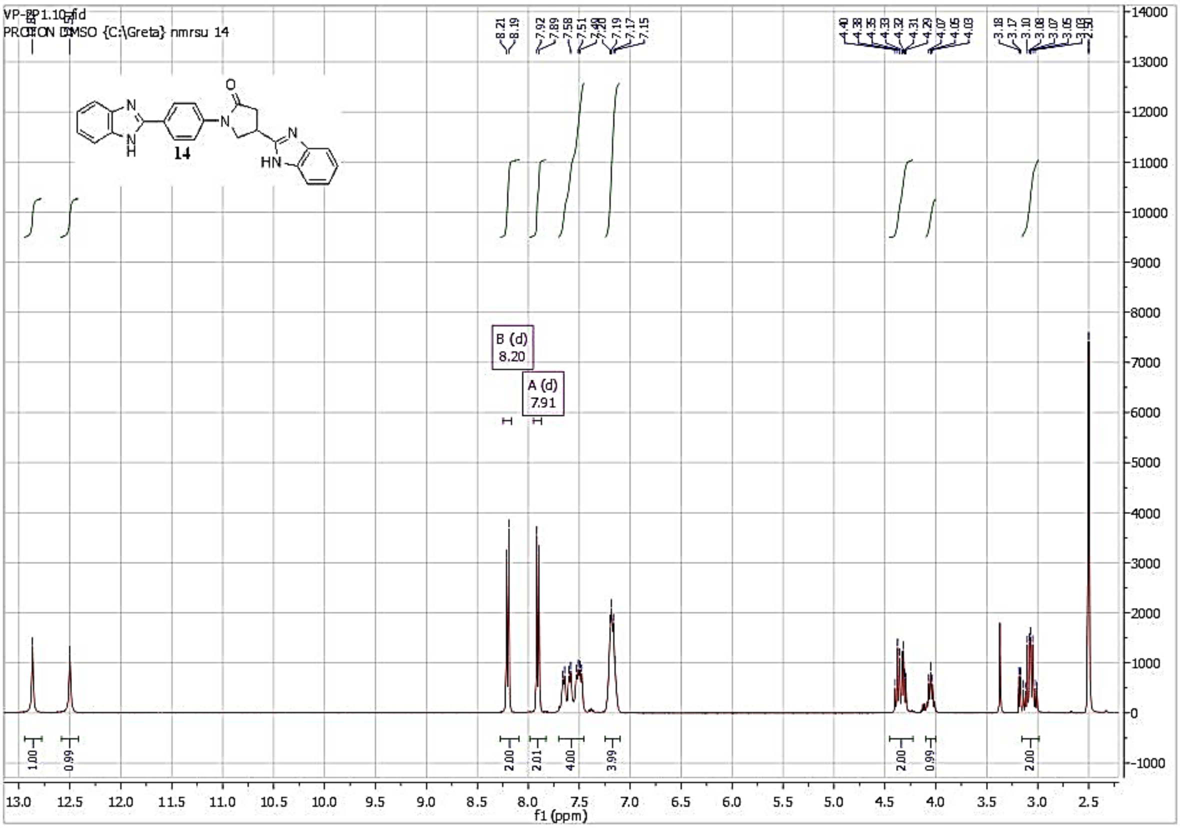

Figure S26: *^{1}H NMR of compound* ***14***

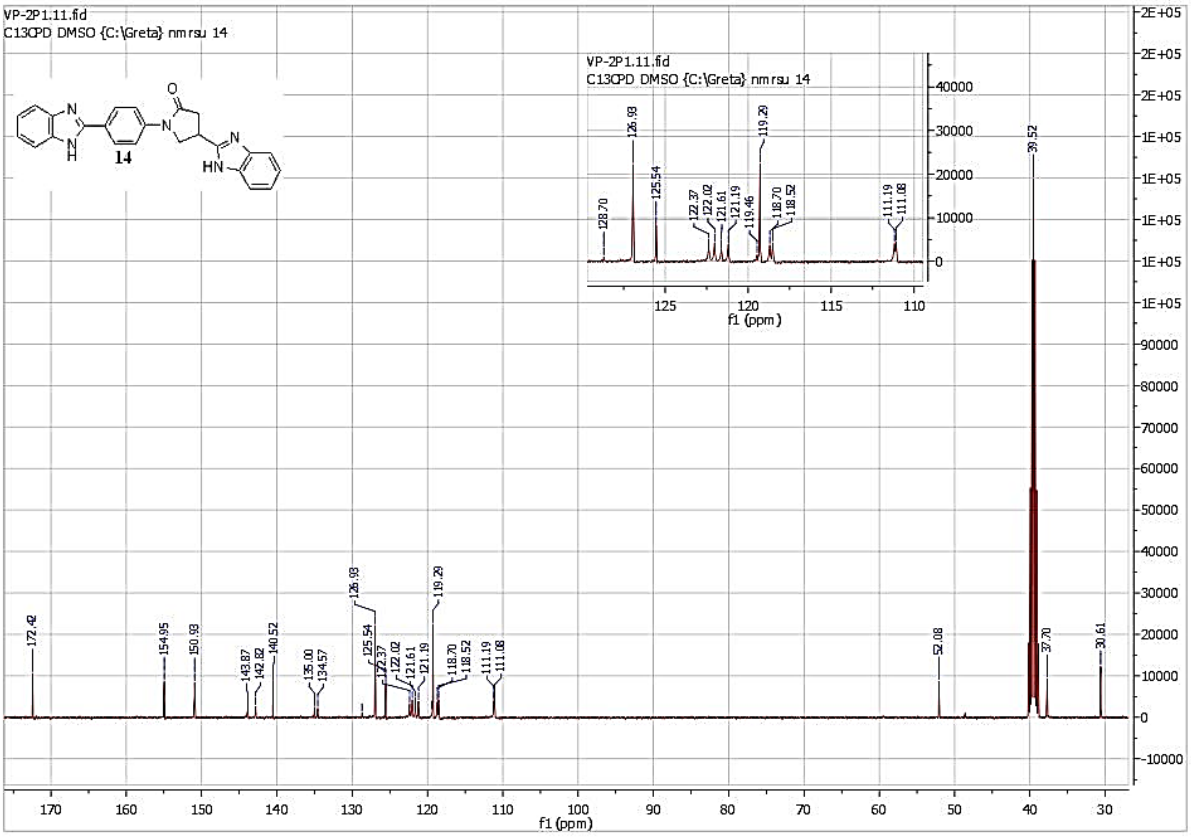

Figure S27: *^{13}C NMR of compound* ***14***

3-(6-methyl-1H-benzimidazol-2-yl)-1-[4-(6-methyl-1H-benzimidazol-2-yl)phenyl]pyrrolidin-5-one 15

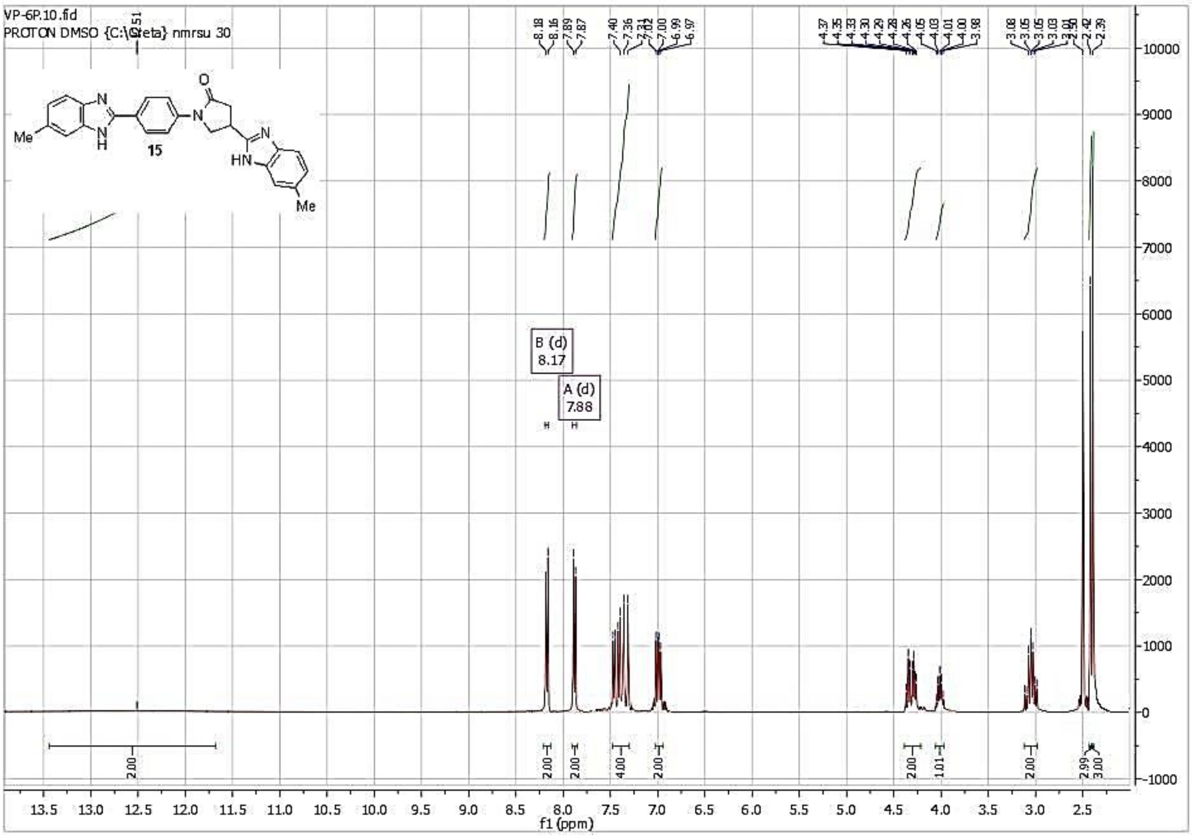

Figure S28: *^{1}H NMR of compound* ***15***

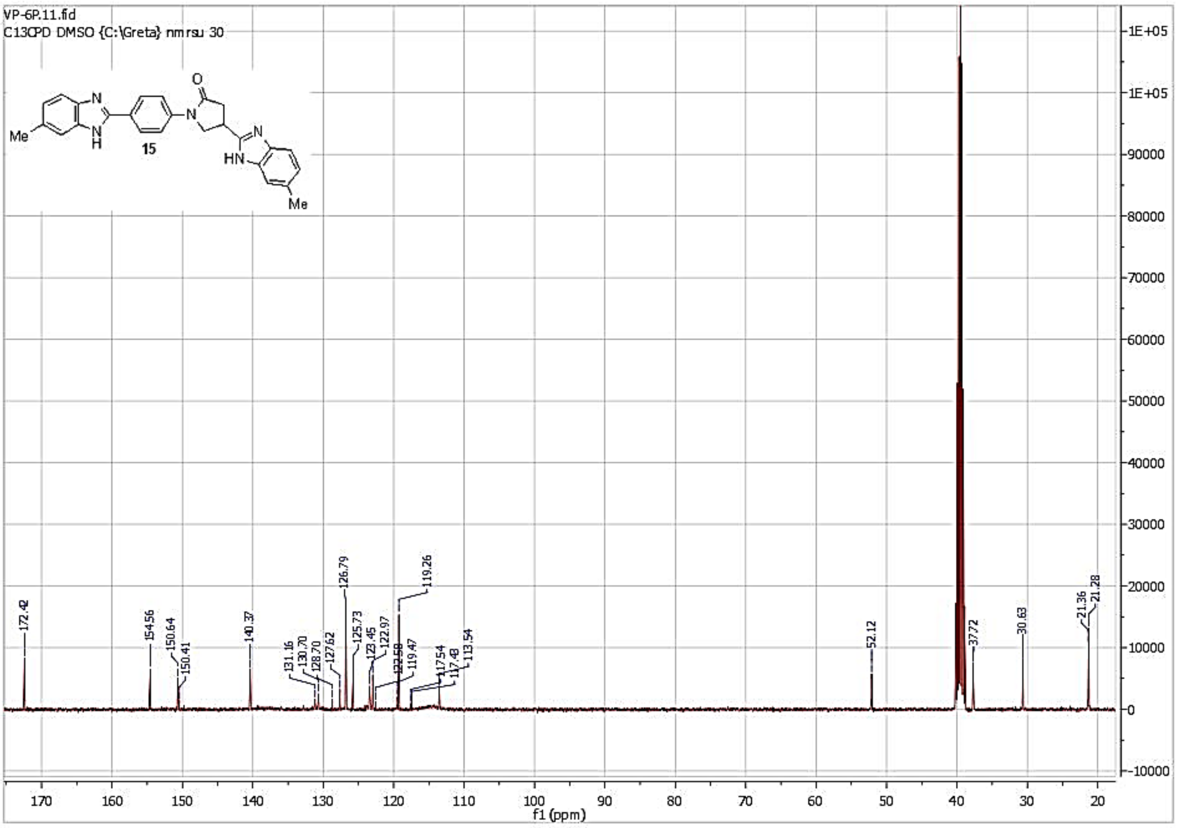

Figure S29: *^{13}C NMR of compound* ***15***

3-(1H-benzimidazol-2-yl)-4-[4-(1H-benzimidazol-2-yl)anilino]butanoic acid 16

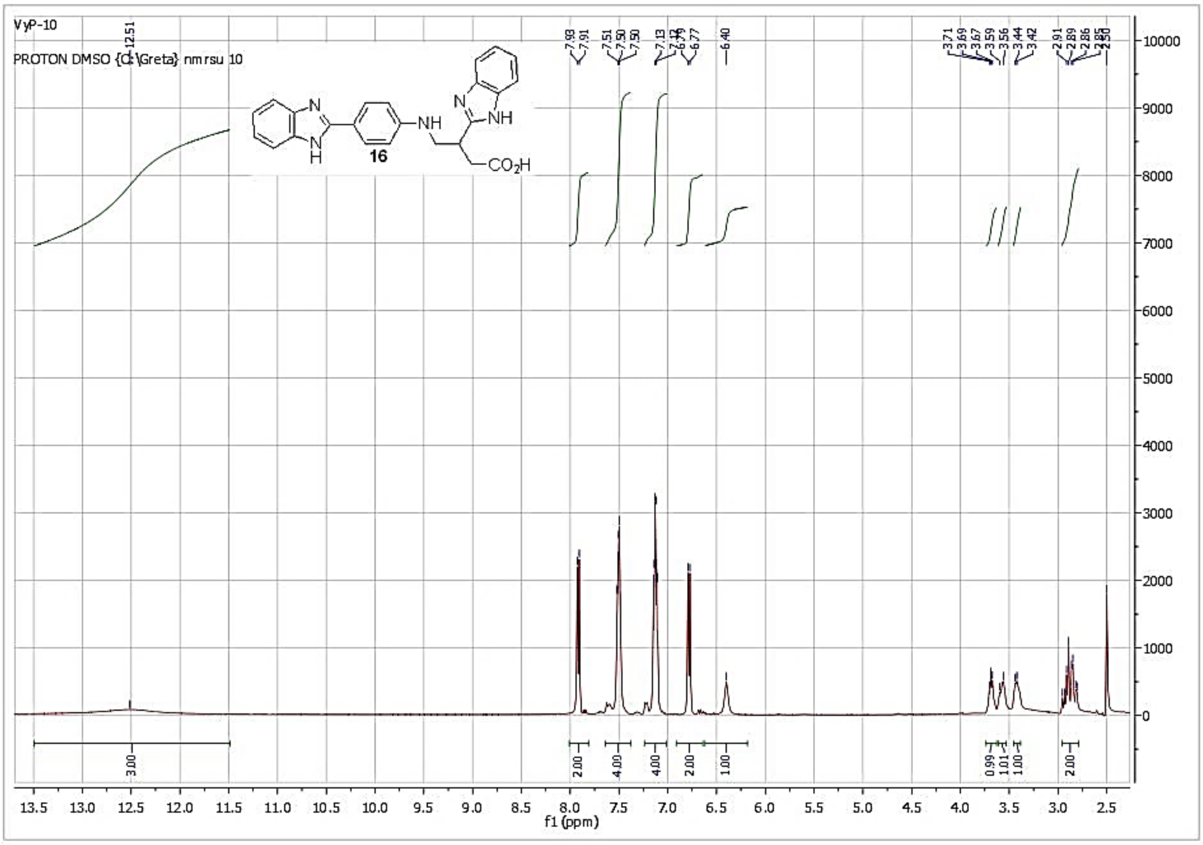

Figure S30: *^{1}H NMR of compound* ***16***

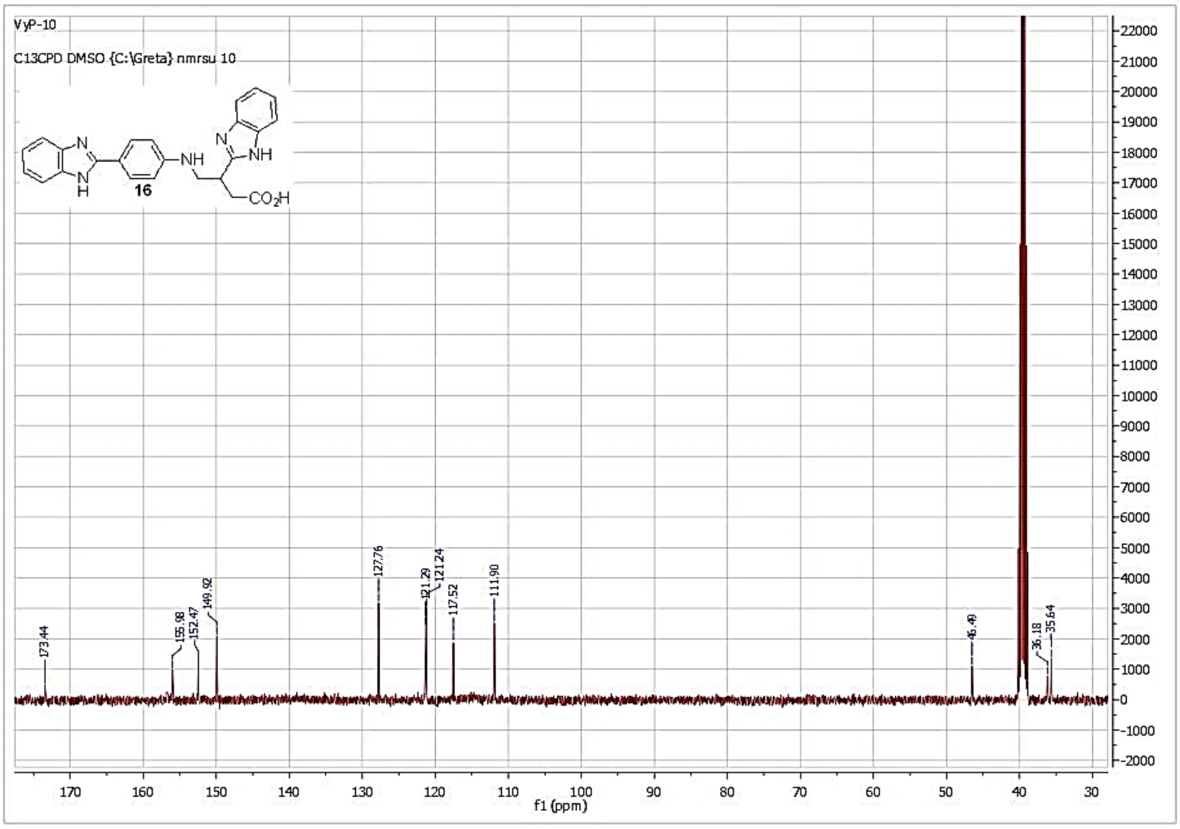

Figure S13: *^{13}C NMR of compound* ***16***

4-((4-(1H-benzimidazol-2-yl)phenyl)amino)-3-(1H-benzimidazol-2-yl)butanehydrazide 17

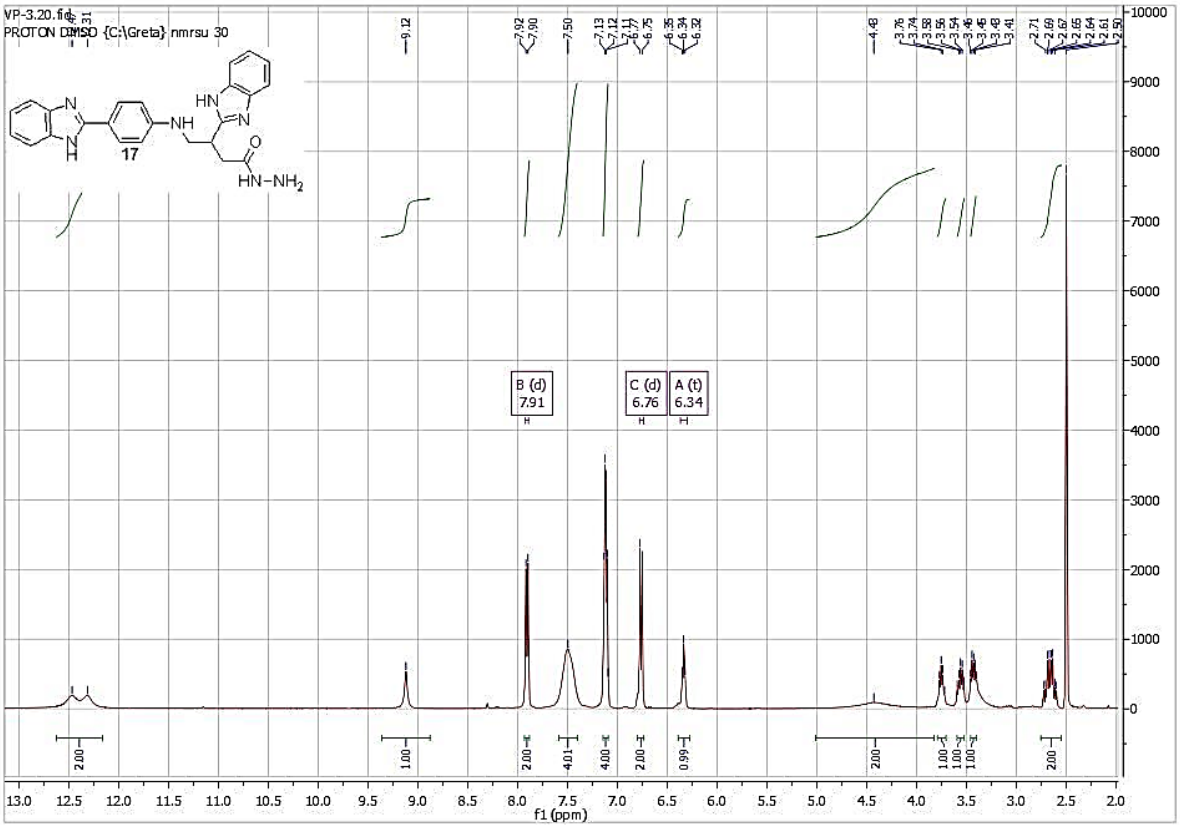

Figure S32: *^{1}H NMR of compound* ***17***

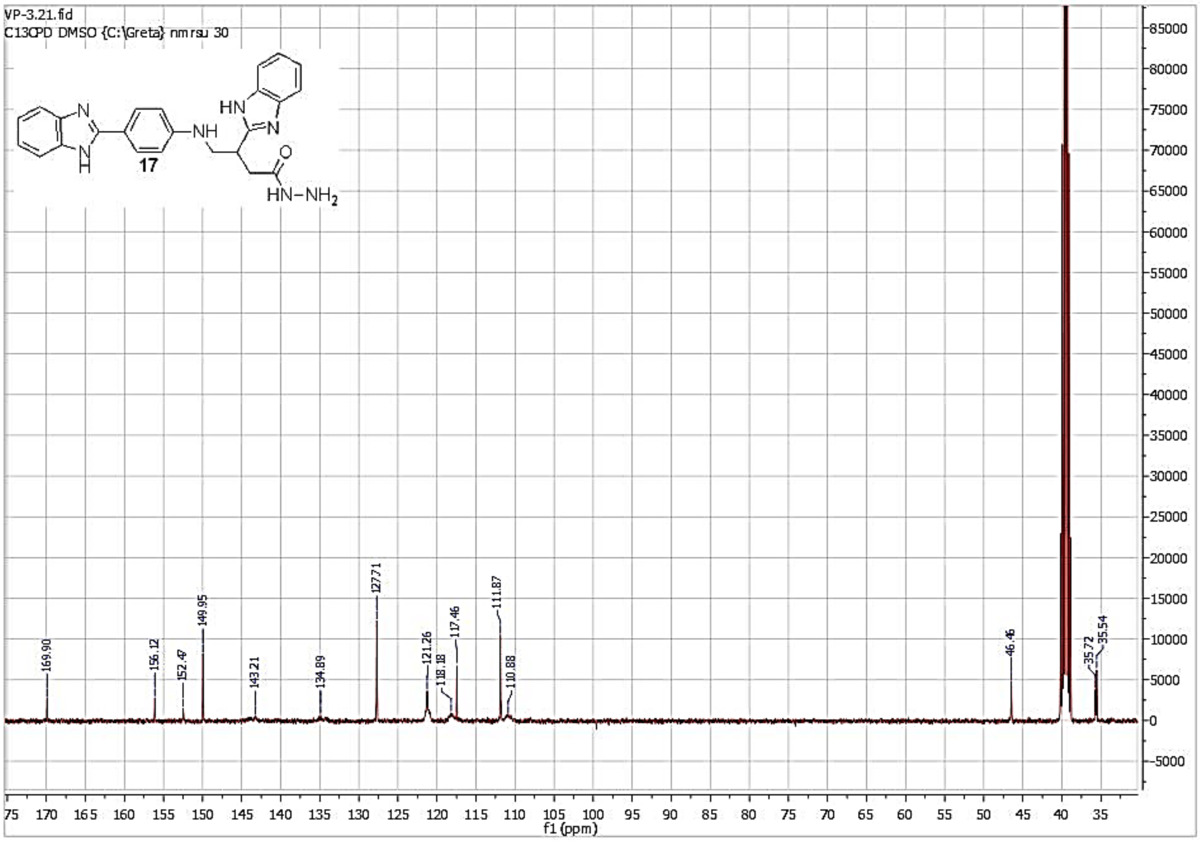

Figure S33: *^{13}C NMR of compound* ***17***

4-((4-(1H-benzimidazol-2-yl)phenyl)amino)-3-(1H-benzimidazol-2-yl)-N-(2,5-dimethyl-1H-pyrrol-2-yl)butanamide 18

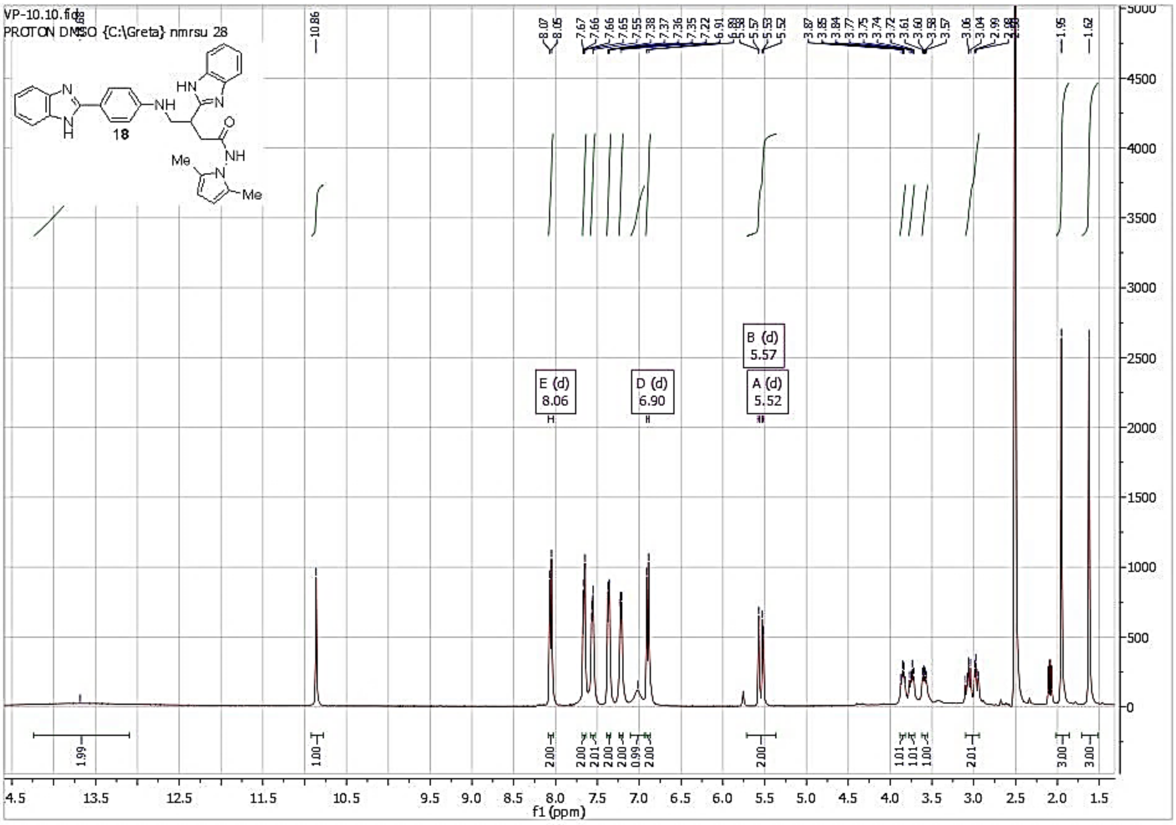

Figure S34: *^{1}H NMR of compound* ***18***

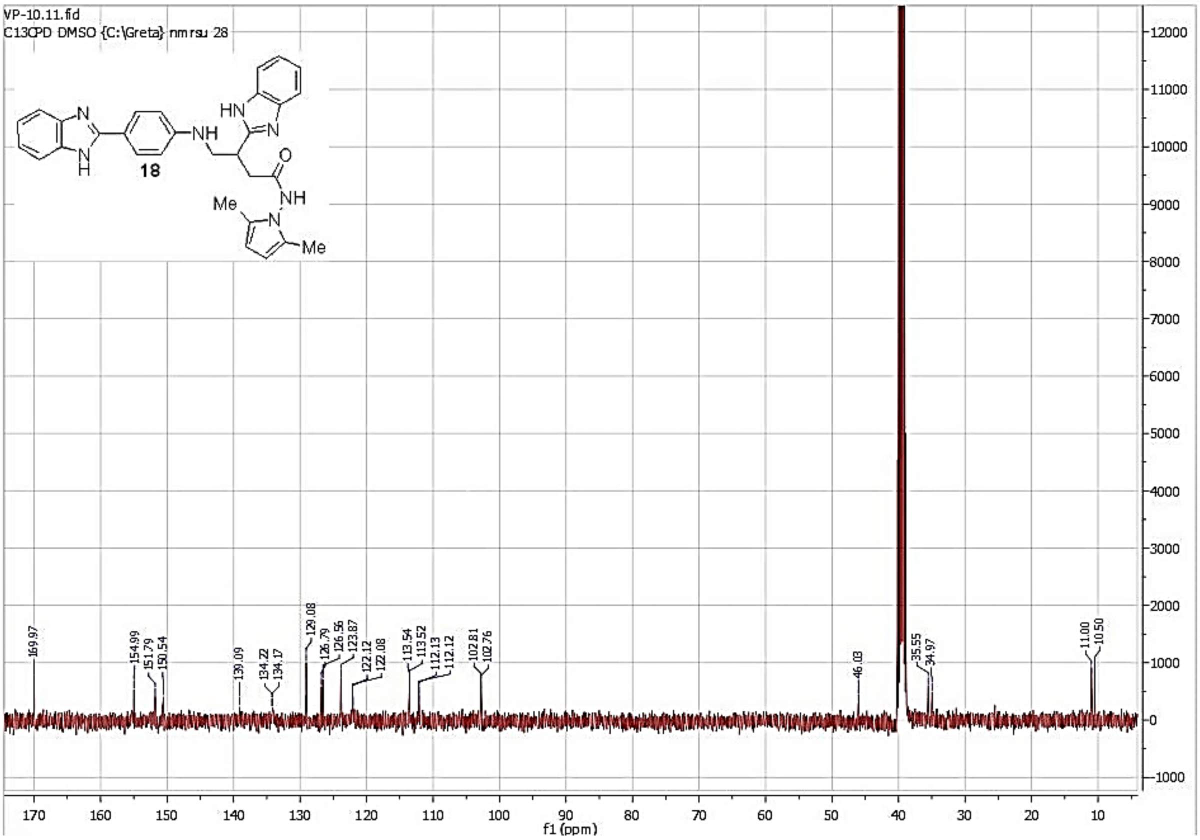

Figure S35: *^{13}C NMR of compound* ***18***

Synthesis of bisbenzimidazoles 19-24

Ethyl 2-(2-(4-(4-(1-(2-ethoxy-2-oxoethyl)-1H-benzimidazol-2-yl)-2-oxopyrrolidin-1-yl)phenyl)-1H-benzimidazol-1-yl)acetate 19

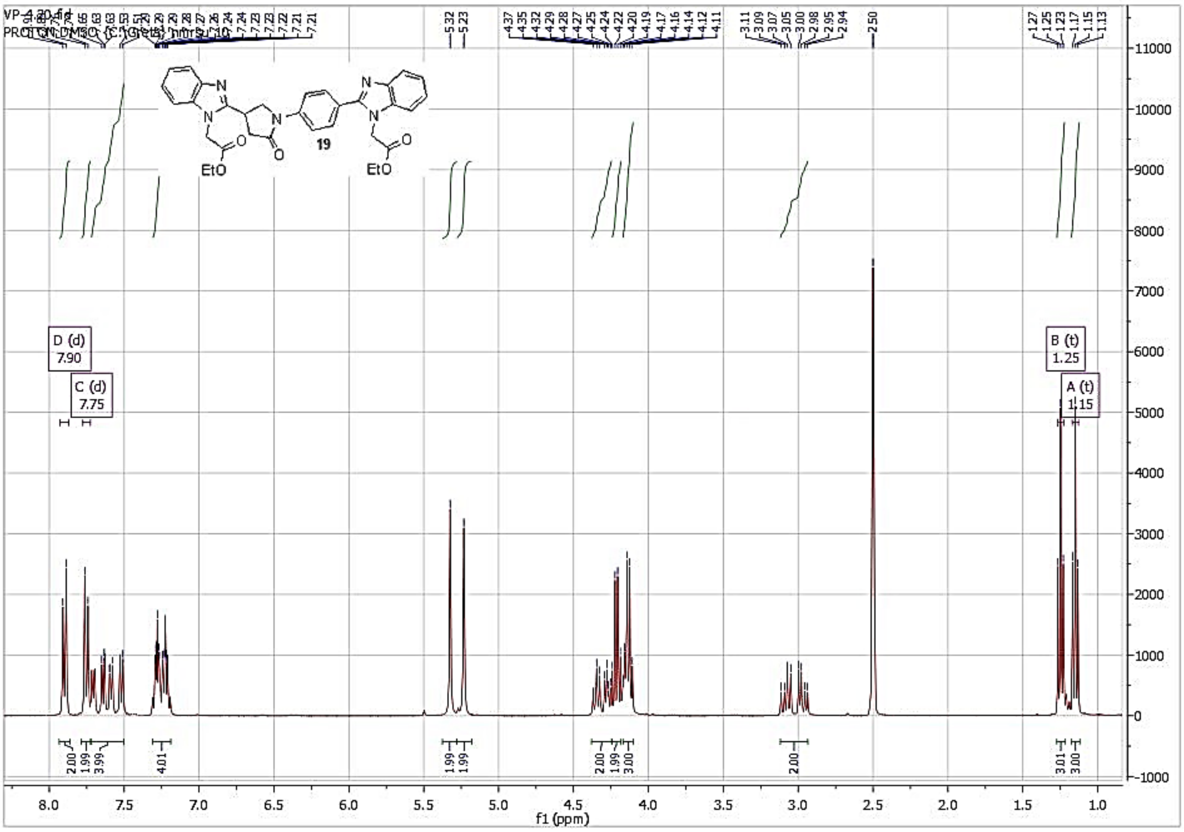

Figure S36: *^{1}H NMR of compound* ***19***

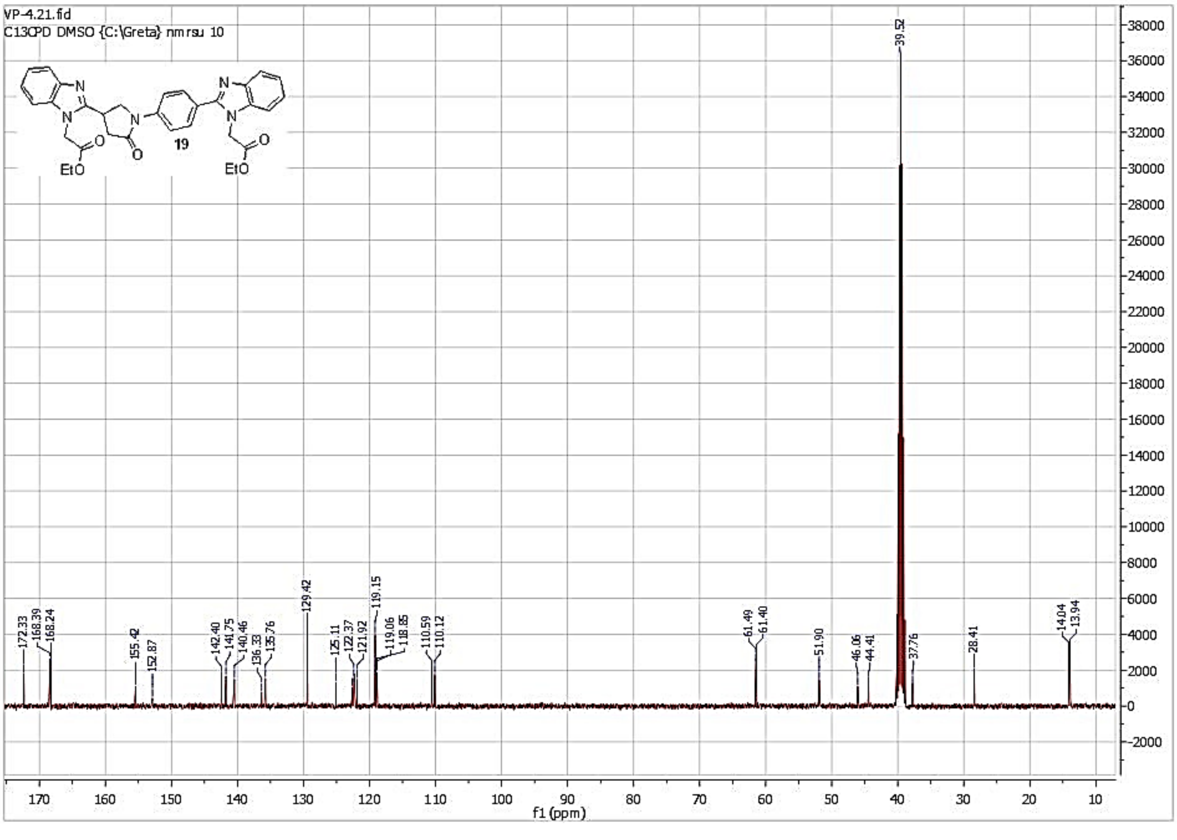

Figure S37: *^{13}C NMR of compound* ***19***

2-(2-(4-(4-(1-(2-hydrazinyl-2-oxoethyl)-1H-benzimidazol-2-yl)-2-oxopyrrolidin-1-yl)phenyl)-1H-benzimidazol-1-yl)acetohydrazide 20

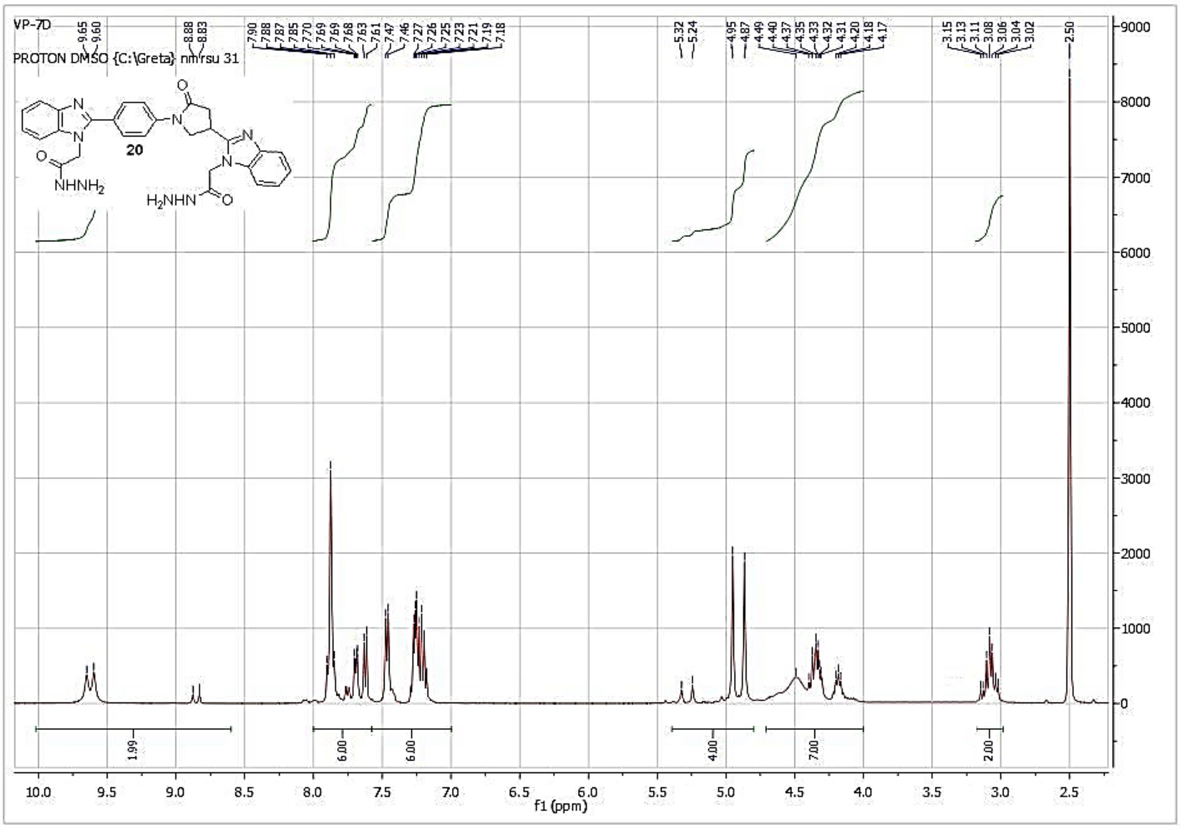

Figure S38: *^{1}H NMR of compound* ***20***

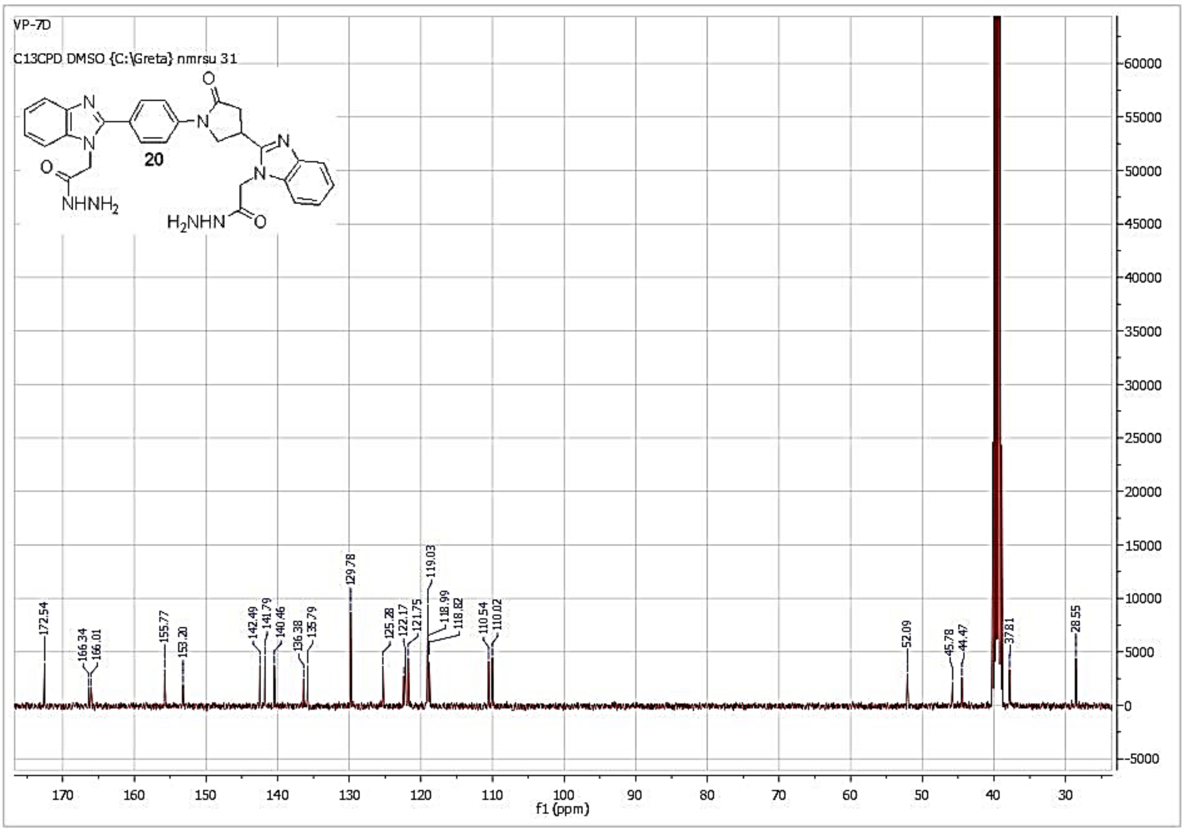

Figure S39: *^{13}C NMR of compound* ***20***

4-(1-(2-(3,5-dimethyl-1H-pyrazol-1-yl)-2-oxoethyl)-1H-benzimidazol-2-yl)-1-(4-(1-(3,5-dimethyl-1H-pyrazol-1-yl)-2-oxoethyl)-1H-benzimidazol-2-yl)phenyl)pyrrolidin-2-one 21

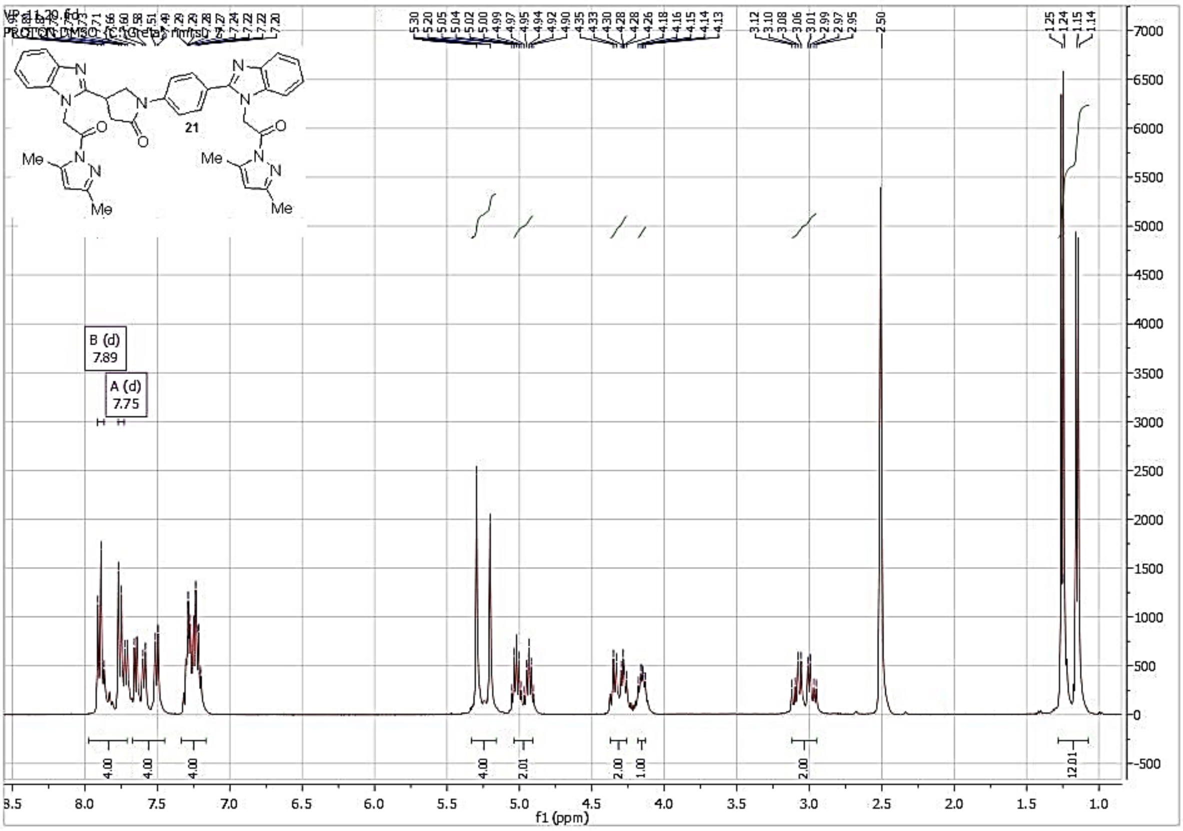

Figure S40: ^{1}H *NMR of compound* ***21***

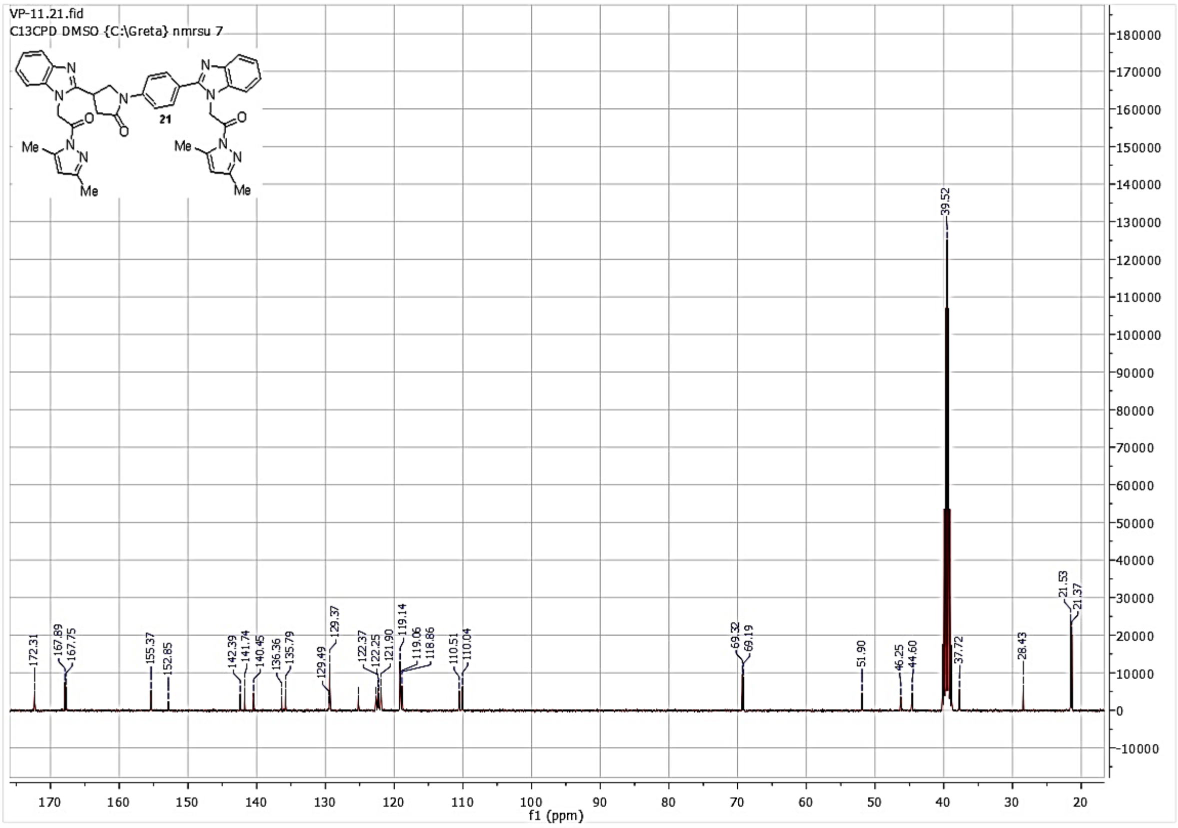

Figure S41: ^{13}C *NMR of compound* ***21***

4-(1-((5-thioxo-4,5-dihydro-1,3,4-oxadiazol-2-yl)methyl)-1H-benzo[d]imidazol-2-yl)-1-(4-(1-((5-thioxo-4,5-dihydro-1,3,4-oxadiazol-2-yl)methyl)-1H-benzo[d]imidazol-2-yl)phenyl)pyrrolidin-2-one 22

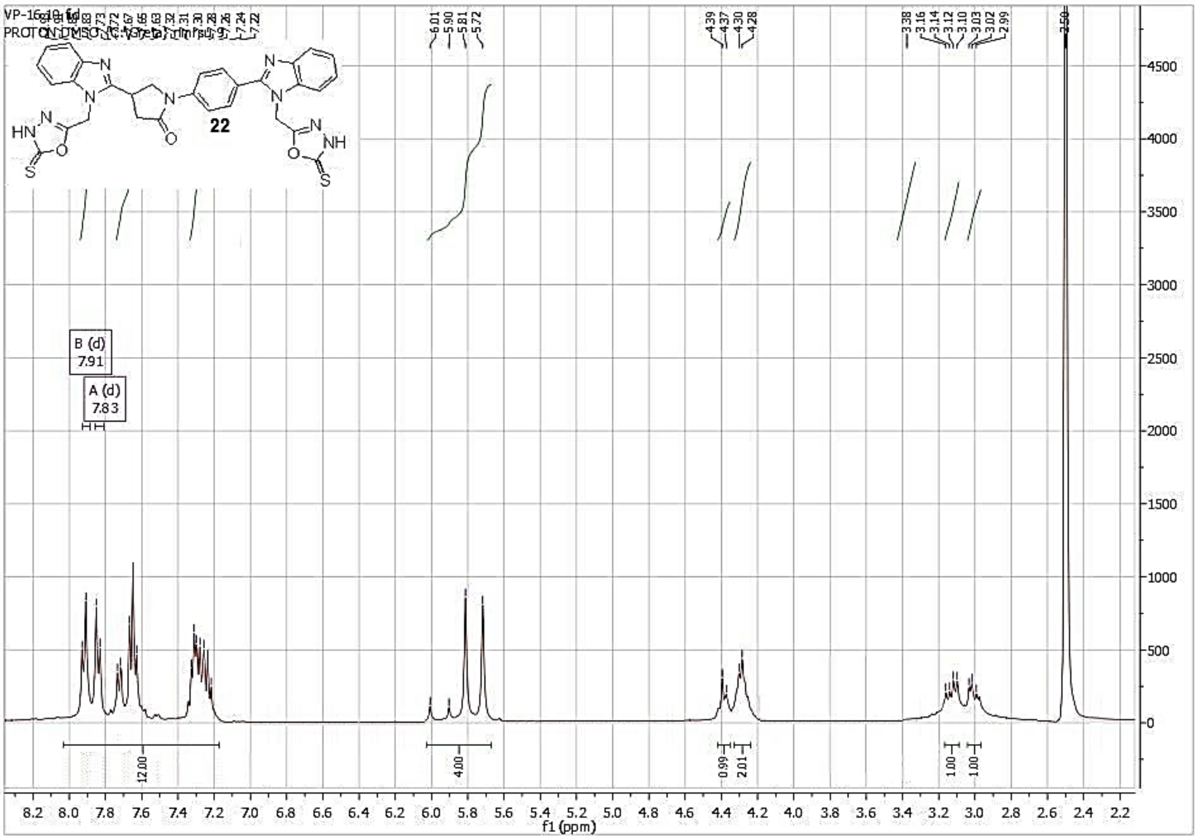

Figure S42: *¹H NMR of compound* **22**

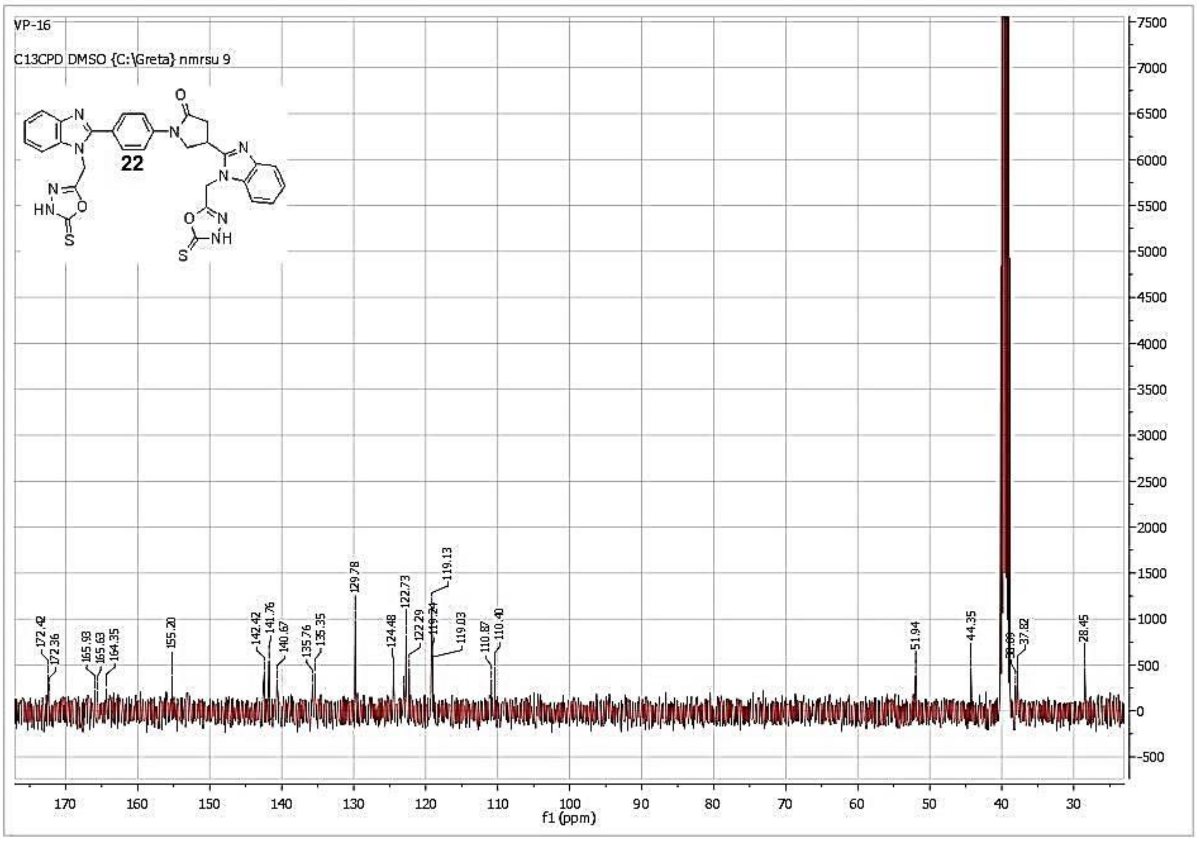

Figure S43: *¹³C NMR of compound* **22**

2-(2-(-2-(5-oxo-1-(4-(1-(2-oxo-2-(2-(phenylcarbamothioyl)hydrazinyl)ethyl)-1H-benzimidazol-2-yl)phenyl)pyrrolidin-3-yl)-1H-benzimidazol-1-yl)acetyl)-N-phenylhydrazin-1-carbothioamide 23

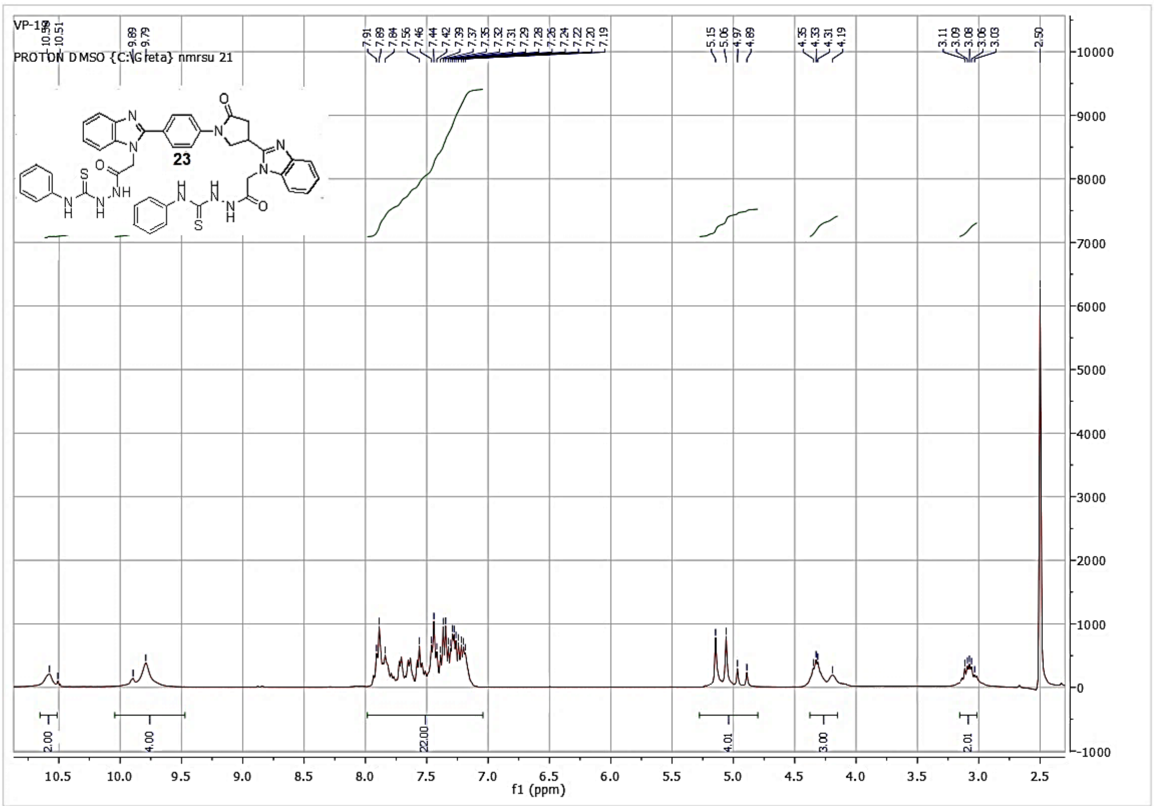

Figure S44: *^{1}H NMR of compound* ***23***

N'-benzylidene-2-(2-(4-(4-(1-(2-(2-(benzylidene)hydrazinyl)-2-oxoethyl)-1H-benzo[d]imidazol-2-yl)-2-oxopyrrolidin-1-yl)phenyl)-1H-benzo[d]imidazol-1-yl)acetohydrazide 24a

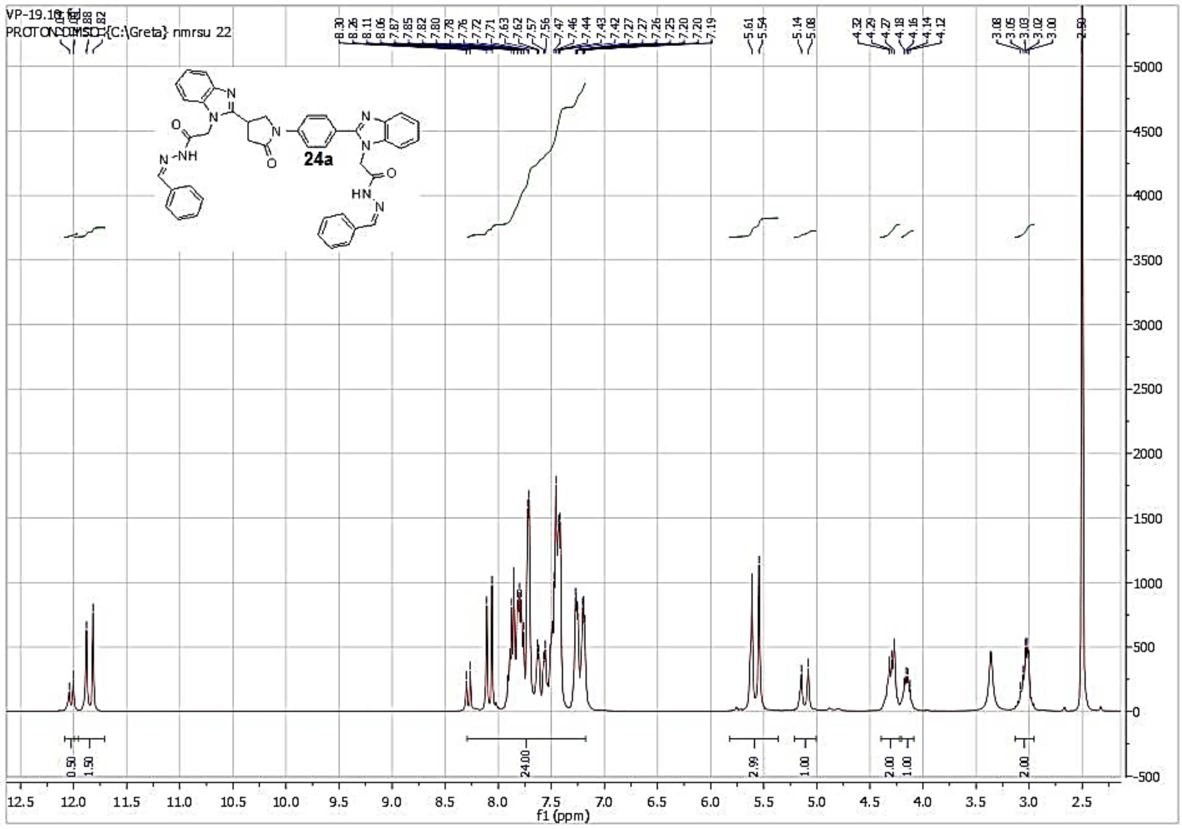

Figure S45: *^{1}H NMR of compound* ***24a***

N'-4-nitrobenzylidene-2-(2-(4-(4-(1-(2-(2-(4-nitrobenzylidene)hydrazinyl)-2-oxoethyl)-1H-benzo[d]imidazol-2-yl)-2-oxopyrrolidin-1-yl)phenyl)-1H-benzo[d]imidazol-1-yl)acetohydrazide 24b

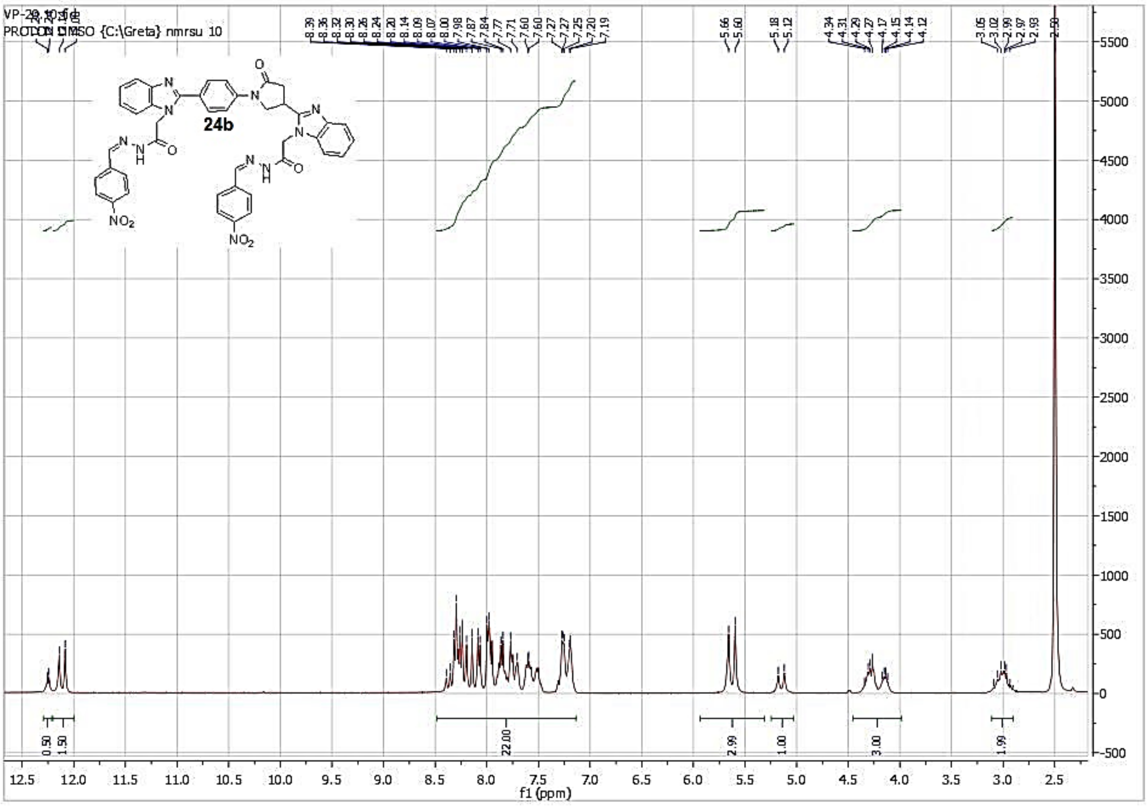

Figure S46: *^{1}H NMR of compound* ***24b***

N'-(4-fluorobenzylidene)-2-(2-(4-(4-(1-(2-(2-(4-fluorobenzylidene)hydrazinyl)-2-oxoethyl)-1H-benzo[d]imidazol-2-yl)-2-oxopyrrolidin-1-yl)phenyl)-1H-benzo[d]imidazol-1-yl)acetohydrazide 24c

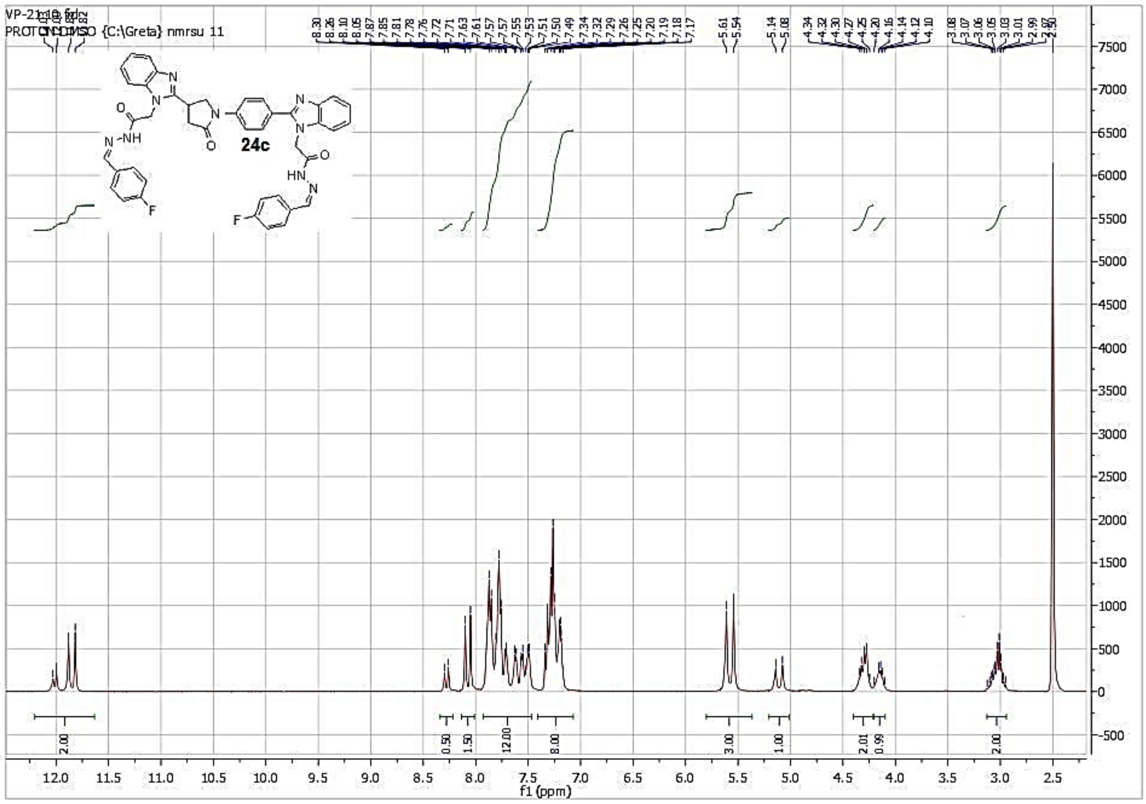

Figure S47: *^{1}H NMR of compound* ***24c***

N'-3-chlorobenzylidene)-2-(2-(4-(4-(1-(2-(2-(3-chlorobenzylidene)hydrazinyl)-2-oxoethyl)-1H-benzo[d]imidazol-2-yl)-2-oxopyrrolidin-1-yl)phenyl)-1H-benzo[d]imidazol-1-yl)acetohydrazide 24d

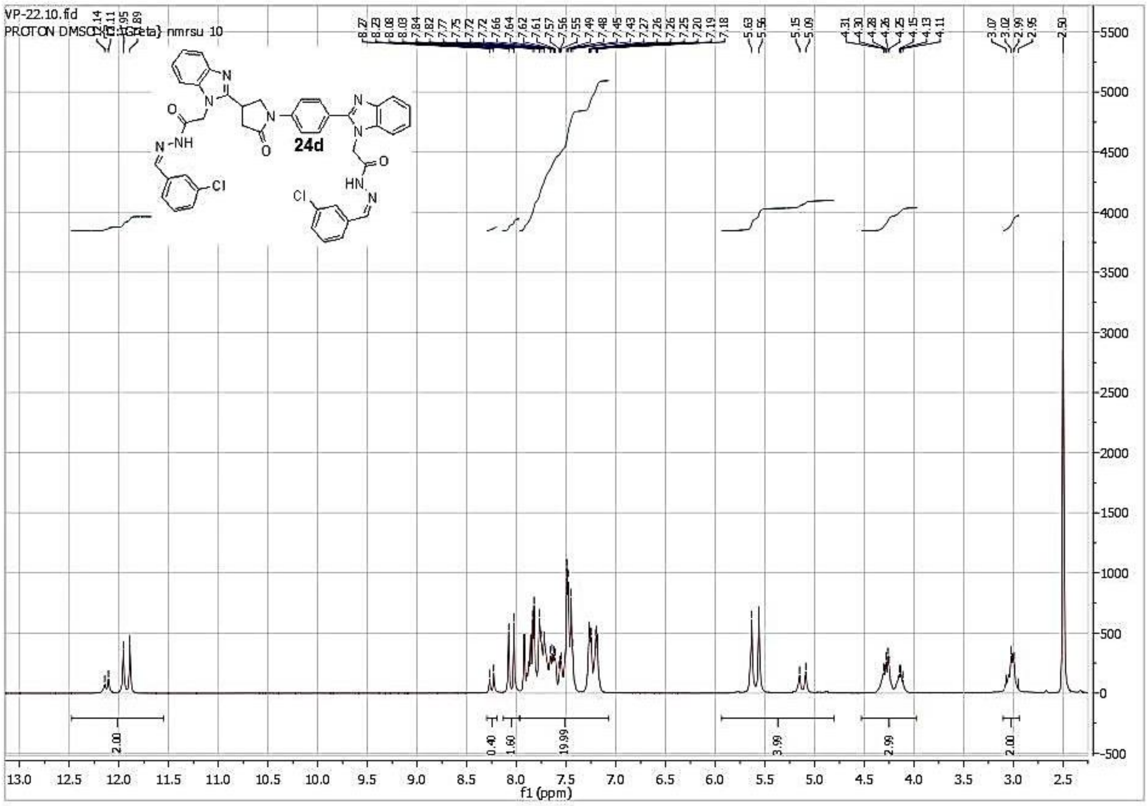

Figure S48: *^{1}H NMR of compound* ***24d***

N'-(2,3-dimethoxybenzylidene)-2-(2-(4-(4-(1-(2-(2-(2,3-dimethoxybenzylidene)hydrazinyl)-2-oxoethyl)-1H-benzo[d]imidazol-2-yl)-2-oxopyrrolidin-1-yl)phenyl)-1H-benzo[d]imidazol-1-yl)acetohydrazide 24e

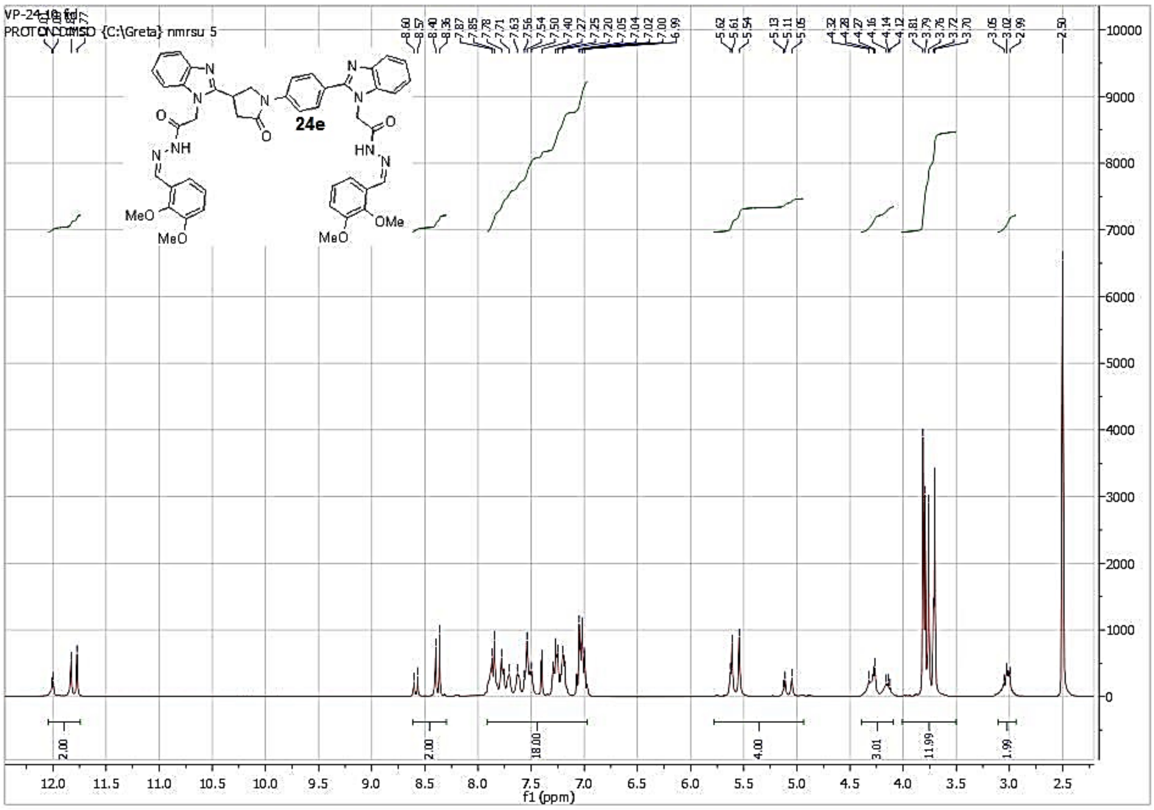

Figure S49: *^{1}H NMR of compound* ***24e***

Supplementary table 1

Bacteria strains used in the biological evaluation

**Organism**	**Strain number**	**Source**
Staphylococcus aureus	ATCC 9144	ATCC[1]
Listeria monocytogenes	ATCC 7644	ATCC[1]
Bacillus cereus	ATCC 11778	ATCC[1]
Escherichia coli	ATCC 8739	ATCC[1]
Pseudomonas aeruginosa	NCTC 6750	NCTC[2]
Salmonella enteritidis	ATCC 13076	ATCC[1]

1. American Type Culture Collection.
2. National Collection of Type Cultures.
